# Instruments to assess post-intensive care syndrome assessment: a scoping review and modified Delphi method study

**DOI:** 10.1186/s13054-023-04681-6

**Published:** 2023-11-07

**Authors:** Nobuto Nakanishi, Keibun Liu, Akira Kawauchi, Masatsugu Okamura, Kohei Tanaka, Sho Katayama, Yuki Mitani, Kohei Ota, Shunsuke Taito, Kenichi Fudeyasu, Yuki Masuka, Shodai Yoshihiro, Shu Utsumi, Mitsuaki Nishikimi, Mamoru Masuda, Yuki Iida, Yusuke Kawai, Junji Hatakeyama, Toru Hifumi, Takeshi Unoki, Daisuke Kawakami, Kengo Obata, Hajime Katsukawa, Hidenori Sumita, Tomoyuki Morisawa, Masahiro Takahashi, Norihiko Tsuboi, Ryo Kozu, Shunsuke Takaki, Junpei Haruna, Yoshihisa Fujinami, Nobuyuki Nosaka, Kyohei Miyamoto, Kensuke Nakamura, Yutaka Kondo, Shigeaki Inoue, Osamu Nishida

**Affiliations:** 1 Division of Disaster and Emergency Medicine, Department of Surgery Related, Kobe University Graduate School of Medicine, 7-5-2 Kusunoki, Chuo-ward, Kobe, 650-0017 Japan; 2 Critical Care Research Group, The Prince Charles Hospital, Brisbane, QLD 4032 Australia; 3 Institute for Molecular Bioscience, The University of Queensland, Brisbane, QLD 4067 Australia; 4 Non-Profit Organization ICU Collaboration Network (ICON), Tokyo, 113-0033 Japan; 5 Department of Critical Care and Emergency Medicine, Japanese Red Cross Maebashi Hospital, 389-1, Asakura-Machi, Maebashi-shi, Gunma, 371-0811 Japan; 6 Berlin Institute of Health Center for Regenerative Therapies (BCRT), Charité – Universitätsmedizin Berlin, Augustenburger Platz 1, 13353 Berlin, Germany; 7 Department of Rehabilitation Medicine, Osaka Police Hospital, 10-31 Kitayama, Tennouji, Osaka, 543-0035 Japan; 8 Department of Rehabilitation Medicine, Okayama University Hospital, 2-5-1 Shikata, Kitaku, Okayama, 700-8558 Japan; 9 Department of Emergency and Critical Care Medicine, Graduate School of Biomedical and Health Sciences, Hiroshima University, 1-2-3 Kasumi, Minami-ku, Hiroshima, 734-8551 Japan; 10 Division of Rehabilitation, Department of Clinical Practice and Support, Hiroshima University Hospital, Hiroshima, 734-8551 Japan; 11 Department of Pharmaceutical Services, Hiroshima University Hospital, Hiroshima, 734-8551 Japan; 12 Department of Physical Therapy, Toyohashi SOZO University School of Health Sciences, 20-1, Matsushita, Ushikawa, Toyohashi, 440-8511 Japan; 13 Department of Nursing, Fujita Health University Hospital, 1-98 Dengakugakubo, Kutsukake-cho, Toyoake, Aichi 470-1192 Japan; 14 Department of Emergency and Critical Care Medicine, Osaka Medical and Pharmaceutical University, 2-7, Daigaku-machi, Takatsuki, Osaka 569-8686 Japan; 15 Department of Emergency and Critical Care Medicine, St. Luke’s International Hospital, 9-1, Akashi-cho, Chuo-ku, Tokyo, 104-8560 Japan; 16 Department of Acute and Critical Care Nursing, School of Nursing, Sapporo City University, Kita 11 Nishi 13, Chuo-ku, Sapporo, 060-0011 Japan; 17 Department of Intensive Care Medicine, Iizuka Hospital, 3-83, Yoshio-machi, Iizuka, Fukuoka 820-8505 Japan; 18 Department of Rehabilitation, Japanese Red Cross Okayama Hospital, 2-1-1 Aoe, Kita-ward, Okayama, 700-8607 Japan; 19 Department of Scientific Research, Japanese Society for Early Mobilization, 1-2-12, Kudan-kita, Chiyoda-ku, Tokyo, 102-0073 Japan; 20 Clinic Sumita, 305-12, Minamiyamashinden, Ina-cho, Toyokawa, Aichi 441-0105 Japan; 21 Department of Physical Therapy, Juntendo University, 2-1-1, Hongo, Bunkyo-ku, Tokyo, 113-0033 Japan; 22 Department of Rehabilitation, Sapporo General Hospital, Kita11-Nishi13, Chuou-ku, Sapporo, Hokkaido 060-8604 Japan; 23 Department of Critical Care and Anesthesia, National Center for Child Health and Development, 2-10-1 Okura, Setagaya, Tokyo, 157-8535 Japan; 24 Department of Rehabilitation Medicine, Nagasaki University Hospital, 1-7-1 Sakamoto, Nagasaki, 852-8501 Japan; 25 Department of Physical Therapy Science, Nagasaki University Graduate School of Biomedical Sciences, 1-7-1 Sakamoto, Nagasaki, 852-8520 Japan; 26 Department of Critical Care Medicine, Yokohama City University Hospital, 3-9 Fukuura, Kanazawa-ku, Yokohama, Kanagawa 236-0004 Japan; 27 Department of Intensive Care Medicine, School of Medicine, Sapporo Medical University, South-1, West-16, Chuo-ku, Sapporo, Hokkaido 060-8543 Japan; 28 Department of Emergency Medicine, Kakogawa Central City Hospital, 439 Kakogawacho Honmachi, Kakogawa-city, Hyogo 675-8611 Japan; 29 Department of Intensive Care Medicine, Graduate School of Medical and Dental Sciences, Tokyo Medical and Dental University, 1-5-45 Yushima, Bunkyo-ku, Tokyo, 113-8510 Japan; 30 Department of Emergency and Critical Care Medicine, Wakayama Medical University, 811-1 Kimiidera, Wakayama, Wakayama, 641-8509 Japan; 31 Department of Emergency and Critical Care Medicine, Juntendo University Urayasu Hospital, Urayasu, 279-0021 Japan; 32 Department of Anesthesiology and Critical Care Medicine, Fujita Health University School of Medicine, 1-98 Dengakugakubo, Kutsukake-cho, Toyoake, Aichi 470-1192 Japan

**Keywords:** Activities of daily living, Cognitive function, Mental illness, Physical function, Post-intensive care syndrome, Quality of life

## Abstract

**Background:**

The assessment of post-intensive care syndrome (PICS) is challenging due to the numerous types of instruments. We herein attempted to identify and propose recommendations for instruments to assess PICS in intensive care unit (ICU) survivors.

**Methods:**

We conducted a scoping review to identify PICS follow-up studies at and after hospital discharge between 2014 and 2022. Assessment instruments used more than two times were included in the modified Delphi consensus process. A modified Delphi meeting was conducted three times by the PICS committee of the Japanese Society of Intensive Care Medicine, and each score was rated as not important (score: 1–3), important, but not critical (4–6), and critical (7–9). We included instruments with ≥ 70% of respondents rating critical and ≤ 15% of respondents rating not important.

**Results:**

In total, 6972 records were identified in this scoping review, and 754 studies were included in the analysis. After data extraction, 107 PICS assessment instruments were identified. The modified Delphi meeting reached 20 PICS assessment instrument recommendations: (1) in the physical domain: the 6-min walk test, MRC score, and grip strength, (2) in cognition: MoCA, MMSE, and SMQ, (3) in mental health: HADS, IES-R, and PHQ-9, (4) in the activities of daily living: the Barthel Index, IADL, and FIM, (5) in quality of life: SF-36, SF-12, EQ-5D-5L, 3L, and VAS (6), in sleep and pain: PSQI and Brief Pain Inventory, respectively, and (7) in the PICS-family domain: SF-36, HADS, and IES-R.

**Conclusion:**

Based on a scoping review and the modified Delphi method, 20 PICS assessment instruments are recommended to assess physical, cognitive, mental health, activities of daily living, quality of life, sleep, and pain in ICU survivors and their families.

**Supplementary Information:**

The online version contains supplementary material available at 10.1186/s13054-023-04681-6.

## Background

Critically ill patients have prolonged physical, cognitive, and mental issues, termed post-intensive care syndrome (PICS) [1]. In a previous study, approximately 60% of patients exhibited a PICS symptom 6 months after hospital discharge [2]. Similarly, PICS was observed in approximately 60% of patients with Coronavirus Disease 2019 (COVID-19) 6 months after hospital discharge [3]. PICS symptoms are obstacles to daily life and returning to work, and, thus, decrease quality of life (QOL) [4]. It is important to assess PICS after intensive care and follow-up the screened population [5].

The concept of PICS was proposed in 2013 by Needham et al. [6]. Following this proposal, research on PICS increased. Turnbull et al. conducted a scoping review on instruments to assess outcomes between 1970 and 2013 [7] and showed that numerous PICS assessment tools were used without properly standardized recommendations. Needham et al. performed an international modified Delphi consensus study to identify PICS assessment instruments for acute respiratory failure survivors [8]. This international modified Delphi consensus study did not reach a consensus for various instruments, and the Society of Critical Care Medicine proposed the recommendation of PICS assessment instruments [5]. Other groups also proposed recommendations for PICS assessment instruments [9–12].

Despite these recommendations, recent studies used different PICS assessment instruments [2, 13]. Some studies employed the Short-Memory Questionnaire (SMQ) to assess cognitive impairments [2, 3], which was not included in previous recommendations. A meta-analysis revealed variations in PICS assessment instruments among studies [13]. Therefore, it is still unclear how frequently the recommended PICS assessment instruments are used in research and clinical practice. Ten years have passed since the scoping review by Turnbull et al. [7], and, thus, an update to investigate how PICS assessment instruments have been used is required.

We conducted a two-step process involving a scoping review and modified Delphi method to investigate the recommendations of PICS assessment instruments. We initially performed a scoping review to identify, which PICS assessment instruments are frequently used. We then held a modified Delphi meeting on the screened instruments to create recommendations in clinical practice. Since clinical usefulness is based not only on the frequency of use, but various aspects are also important for identifying the appropriate recommendation to assess PICS, including scientific evidence, convenience, such as online or telephone assessments, time for the assessment, and cost. Therefore, in this Delphi process, we did not focus on a specific condition, but rather on various diseases, environments, and evaluators for applications under any condition.

## Methods

### Study design

**Fig. 1:**
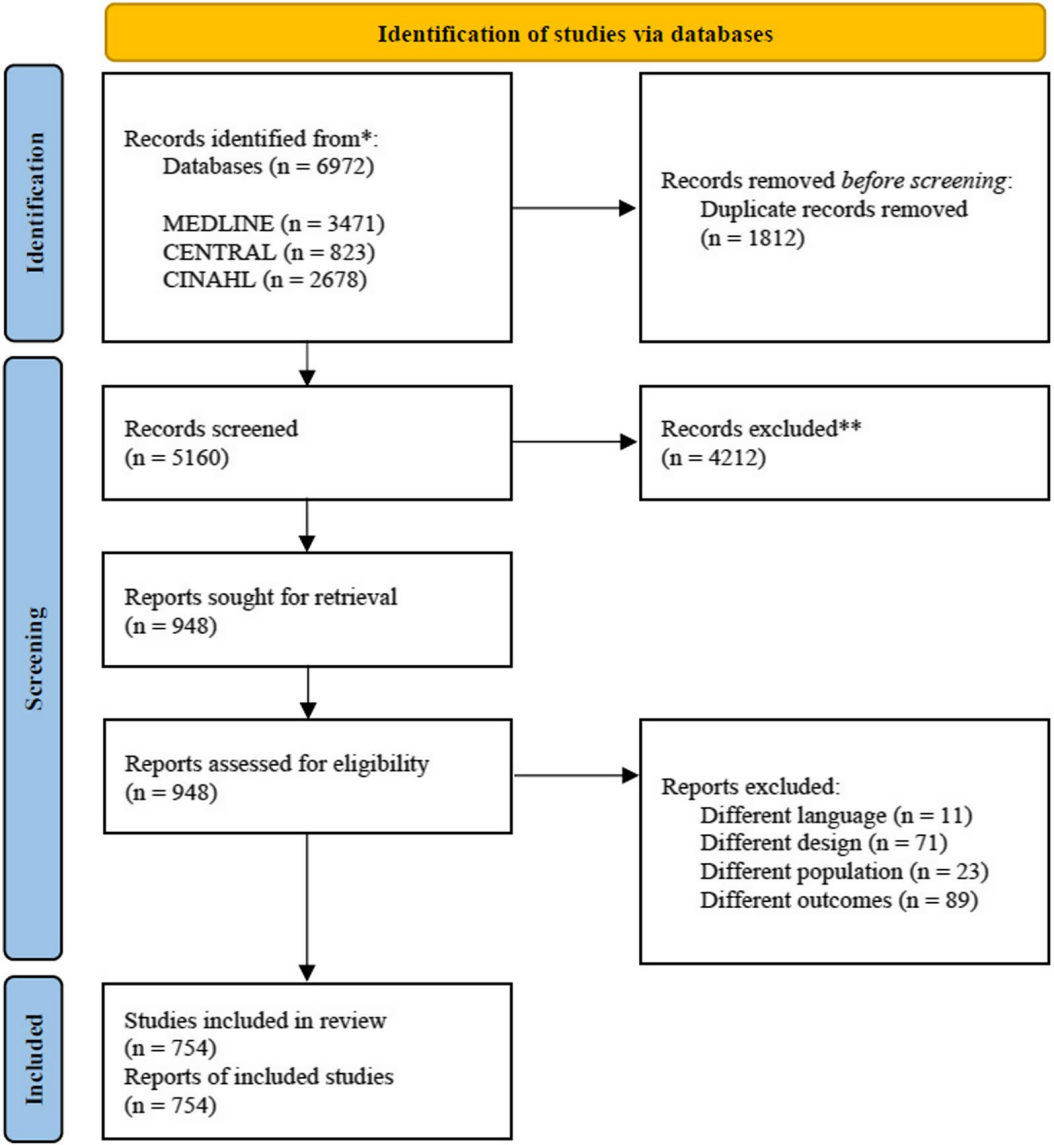
Illustration of the scoping review and modified Delphi consensus process. CENTRAL: Cochrane Central Register of Controlled Trials, MEDLINE: Medical Literature Analysis and Retrieval System Online, CINAHL: Cumulative Index to Nursing and Allied Health Literature, ADL: activities of daily living, QOL: quality of life, JSICM: the Japanese Society of Intensive Care Medicine, PICS: post-intensive care syndrome

The present study aimed to reach to a consensus on PICS assessment instruments. The study design was based on a scoping review and modified Delphi method (Fig. 1). The scoping review was conducted to identify eligible assessment methods that have frequently been reported in research. The Delphi method has been used to evaluate methods recommended by members with extensive experience on PICS. Since the scoping review and Delphi method were used, we did not obtain approval from an ethics committee. The present study was registered as a clinical trial (UMIN Clinical Trials Registry: 000049634).

### Scoping review

In this scoping review based on Arksey and O’Malley’s 5-stage framework and PRISMA [14, 15], we searched for common evaluation instruments on the physical, cognitive, mental health, QOL, ADL, other, and family domains of ICU survivors. Since a previous scoping review searched until 2013 [7], we investigated the following databases between 2014 and 2022: Cochrane Central Register of Controlled Trials (CENTRAL) in the Cochrane Library, Medical Literature Analysis and Retrieval System Online (MEDLINE) via PubMed, and Cumulative Index to Nursing and Allied Health Literature (CINAHL). The key search terms are listed in the Additional file 1. We did not ask the authors of original studies for unpublished or additional data.

### Data extraction and study selection in the scoping review

After record identification, data were exported into Endnote, and duplicates were deleted. After data were imported into Rayan from Endnote, 1st and 2nd screenings were conducted by four reviewers with reliable interrater reproducibility (κ value of 0.78 [95% CI: 0.68 to 0.87]), based on a previous study [16]. The 1st screening was conducted from the titles and abstracts of each set of retrieved data. We included studies on adult ICU survivors (≥ 18 years of age) and their family members at hospital discharge or thereafter. The study design included retrospective, observational, and randomized controlled trials regardless of any intervention. We excluded reviews, protocols, trial registries, case reports, conference abstracts, and studies in languages other than English. In the case of a disagreement between reviewers, a third reviewer adjudicated when needed. The 2nd screening was conducted on full texts. We included studies that assessed any PICS outcome at hospital discharge or thereafter. Exclusion criteria were classified into different languages, designs, populations, and outcomes. Data from eligible articles were extracted into the data collection format by reviewers. Data were separately input into categories at or after hospital discharge. Extraction was based on methods to assess the following outcomes: (1) physical function, (2) cognitive function, (3) mental health, (4) ADL, (5) QOL, (6) other, and (7) family domains.

### The Delphi consensus process

A three-round, modified Delphi consensus process was conducted to identify desirable instruments to assess PICS. Based on the scoping review, we selected outcome evaluation instruments used more than two times. Voting was conducted three times online by the 23 members of the Japanese Society of Intensive Care Medicine PICS committee and working group members, consisting of 14 physicians, 6 physiotherapists, and 3 nurses. The evaluation of these instruments was based on the Consensus-based Standards for the selection of health Measurement Instruments (COSMIN) checklist [17]. The information provided was summarized and attached in the Additional file 1. Voters rated outcome assessment instruments using the Grading of Recommendations Assessment, Development, and Evaluation (GRADE) scale as follows: not important (score: 1–3), important, but not critical (4–6), and critical (7–9) [18]. The evaluation was based on the clinical usefulness of the PICS evaluation, including scientific evidence and the published frequency assessed through the scoping review. Scoring included the usefulness of both screening and follow-ups. In the objective evaluation, we examined the assessment consensus as follows: When (evaluation timing): at and after hospital discharge, Where: hospital, outpatients’ clinic, and telephone interviews, Who (person to use the scores): not specific and includes voters or other staff, Whom (patients to be assessed): the general population and not limited to a specific disease, How: convenience, time for the assessment, and cost. Convenience for use in Japan, such as a verified Japanese version, was not taken into consideration. A consensus was defined as ≥ 70% of respondents rating critical and ≤ 15% of respondents rating not important in the 3rd round of the modified Delphi meeting. Scoring results and comments were provided to the voters in rounds 2 and 3, and voters reevaluated outcome assessment instruments.

## Results

### Literature Search

**Fig. 2:**
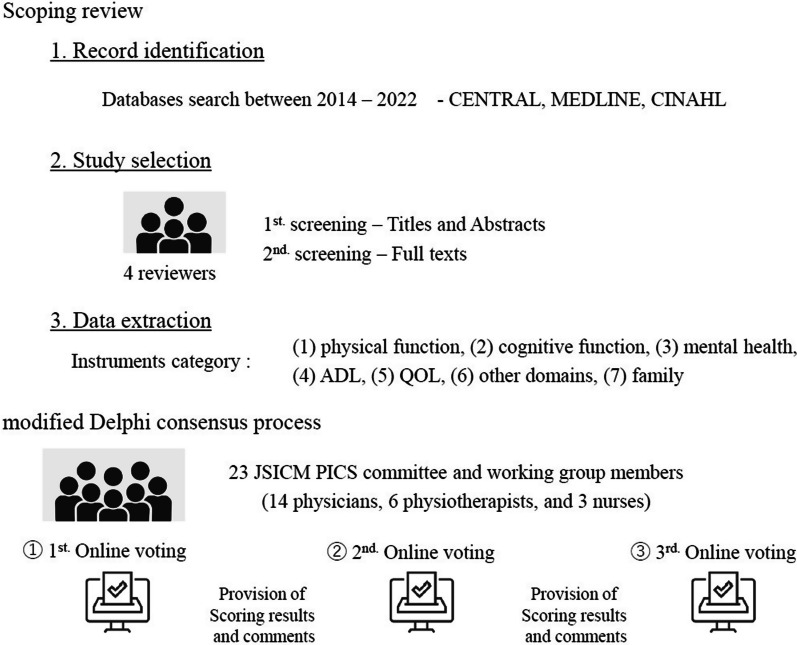
Flowchart for selecting articles in the systematic review. CENTRAL, Cochrane Central Register of Controlled Trials; MEDLINE, Medical Literature Analysis and Retrieval System Online; CINAHL, Cumulative Index to Nursing and Allied Health Literature

The PRISMA Flow Diagram in Fig. 2 shows the article selection process in the scoping review. The search strategy identified 6972 records, of which 754 were included in the analysis. Among 754 included studies, 114 (15%) were related to COVID-19. The timing of the PICS evaluation varied among studies, and multiple follow-ups were counted separately. Among 995 follow-ups, evaluations were frequently conducted after 3 months in 253 (25%), after 12 months in 212 (21%), and after 6 months in 204 (21%). Follow-ups were counted from hospital discharge in 239 (42%), ICU discharge in 211 (37%), and ICU admission in 120 (21%).

### Data extraction

All 754 records were shown in the Additional file 1: Table S1. Among 754 records, we extracted PICS assessment instruments, which are shown in Additional file 1: Tables S2 and S3. We included the following number of PICS assessment instruments from those used more than 2 times: (1) 23 items in physical function, (2) 14 in cognitive function, (3) 24 in mental health, (4) 13 in ADL, (5) 9 in QOL, (6) other: 3 in sleep, 4 in pain, 2 in fatigue, and 2 other items, and (7) 13 in family (Table 1).

**Table 1:** Extracted PICS assessment after hospital discharge

Post-Intensive Care Syndrome categories	Frequency of use		Modified Delphi consensus process				
	After hospital discharge	At hospital discharge	1st average	2nd average	3rd average	3rd ≤ 3	3rd ≥ 7
Physical function	(number)	(number)	(score)	(score)	(score)	(number)	(number)
6-min walk test	49	11	8.2	8.1	8.0	0	22
Pulmonary function tests (Spirometer, DLCO)	39	2	6.0	5.6	5.6	2	7
Medical Research Council (MRC) score	34	22	8.3	8.1	8.2	0	22
Grip strength	34	20	8.0	7.8	7.8	0	20
Clinical Frailty Scale (CFS)	16	3	7.0	6.8	6.6	0	12
Sit-to-stand test (30 s, 1 min)	15	2	6.1	6.0	5.9	0	8
Short Physical Performance Battery (SPPB)	14	4	6.5	6.2	6.1	1	11
4-m gait speed test	8	0	5.6	5.3	5.2	0	1
Manual muscle test (MMT)	6	2	6.1	5.5	5.5	1	4
MRC dyspnea scale	6	2	5.2	4.7	4.7	3	1
Saint George’s Respiratory Questionnaire (SGRQ)	6	0	4.0	3.3	3.4	12	0
Isometric Quadriceps Strength	6	1	4.9	4.6	4.5	4	0
TUG (Timed Up and Go test)	5	1	5.4	5.2	5.1	2	3
BERG balance test	4	0	4.2	3.8	3.7	11	1
Physical Functional Status (PFS)	4	0	4.5	4.2	4.1	4	0
Chelsea critical care Physical Assessment tool (CPAx)	4	4	4.5	3.7	3.6	11	0
Borg Dyspnea Scale	4	0	4.4	3.7	3.7	10	0
10-min walk test	3	0	3.9	3.4	3.2	12	0
2-min walk test	3	1	4.6	4.3	4.3	4	0
Fried Frailty Criteria	3	0	4.7	4.0	4.0	5	0
Physical Functional test (PFIT) for the ICU	1	8	4.2	3.5	3.6	11	0
Functional Status Score for the ICU (FSS-ICU)	0	4	4.2	3.7	3.7	10	1
Functional Ambulation Categories (FACs)	2	3	4.2	3.8	4.0	8	1
Cognitive function	(number)	(number)	(score)	(score)	(score)	(number)	(number)
Montreal Cognitive Assessment (MoCA)	37	11	8.1	8.3	8.3	0	23
Mini-Mental State Examination (MMSE)	36	16	8.1	8.1	8.0	0	23
Repeatable Battery for the Assessment of Neuropsychological Status (RBANS)	21	1	6.0	5.9	5.8	0	3
Trail Making Test A, B	15	1	5.4	5.3	5.3	0	1
Telephone Interview for Cognitive Status (TICS)	9	0	6.0	6.1	6.1	1	10
Short Memory Questionnaire (SMQ)	7	2	6.7	6.8	7.3	0	21
Informant Questionnaire for Cognitive Decline in the Elderly (IQCODE)	7	2	5.4	5.3	5.3	3	3
Cognitive Failures Questionnaire (CFQ)	7	0	5.0	4.9	4.8	3	1
Cognitive and Affective Mindfulness Scale (CAMS) revised version	5	0	4.3	4.1	4.1	6	1
Wechsler Adult Intelligence Scale (WAIS-IV)	4	1	4.0	3.8	3.7	10	0
Healthy Aging Brain Care monitor self-report tool (HABC monitor)	4	0	4.3	4.0	3.7	12	0
Rey Auditory-Verbal Learning Test (RAVLT)	4	1	3.4	3.1	3.1	17	0
Cambridge Neuropsychological Test Automated Battery (CANTAB)	3	0	4.4	4.0	4.0	7	0
Clinical Dementia Rating (CDR)	3	0	4.2	3.7	3.9	7	0
Mental health	(number)	(number)	(score)	(score)	(score)	(number)	(number)
Hospital Anxiety and Depression Scale (HADS)	155	31	8.8	8.8	8.9	0	23
Impact of Event Scale-Revised (IES-R)	76	22	8.3	8.5	8.7	0	23
Patient Health Questionnaire-9 (PHQ-9)	26	5	7.0	7.0	6.9	0	19
Post-traumatic Stress Scale 10 (PTSS-10)	20	0	6.6	6.5	6.5	0	13
PTSD Checklist for DSM-5 (PCL-5)	18	5	6.5	6.5	6.5	1	14
Beck Depression Inventory-II (BDI-II)	16	0	5.2	5.1	5.2	2	1
Generalized Anxiety Disorder-7 (GAD-7)	13	1	5.7	5.5	5.5	1	3
PTSD Checklist, Civilian version (PCL-C)	12	0	5.5	5.3	5.3	1	2
Impact of Event Scale (IES)	11	0	4.3	4.3	4.4	4	0
Post-Traumatic Stress Scale 14 (PTSS-14)	11	0	5.0	5.2	5.0	2	1
PTSD Checklist-Specific (PCL-S)	9	0	5.3	5.0	5.0	1	1
Impact of Event Scale-6 (IES-6)	8	2	6.2	6.0	6.2	0	10
PHQ-2	7	2	5.2	4.9	4.9	4	2
Numerical Rating Scale (NRS)-Anxiety	5	0	5.0	5.0	5.0	3	1
Depression Anxiety Stress Scale (DASS-21)	5	4	4.9	4.8	4.8	3	0
State Trait Anxiety Inventory (STAI)	5	0	4.1	4.1	4.0	6	0
Center for Epidemiological Studies-Depression Scale (CES-D)	5	0	4.2	3.9	3.7	11	0
Visual Analog Scale (VAS)	4	12	5.4	5.0	4.8	3	1
Brief COPE (Coping Orientation to Problems Experienced) Inventory	4	0	4.1	3.9	3.9	9	0
Trauma Screening Questionnaire	4	0	4.3	4.0	4.0	8	0
Major Depression Inventory (MDI)	3	0	5.0	4.5	4.5	2	0
PHQ-8	3	0	5.3	4.9	5.0	2	2
PHQ-4	3	0	5.1	4.8	4.9	3	2
Davidson Trauma Scale (DTS)	3	0	3.9	3.6	3.4	11	0
ADL	(number)	(number)	(score)	(score)	(score)	(number)	(number)
Barthel Index	47	20	8.6	8.7	8.7	0	23
Instrumental Activities of Daily Living (IADL)	25	0	7.7	7.6	7.6	0	20
Modified Rankin Scale (mRS)	19	4	6.0	6.1	6.0	1	5
Katz Activities of Daily Living	19	0	6.6	6.6	6.4	0	12
Functional Independence Measure (FIM)	12	10	7.1	7.0	6.8	0	18
World Health Organization’s Disability Assessment Schedule (WHODAS 2.0)	11	0	5.8	5.7	5.7	0	4
Glasgow Outcome Scale (GOS)	9	0	4.7	4.7	4.7	2	0
GOS-E (GOS-Extended)	9	0	4.7	4.4	4.3	1	0
Cerebral Performance Category (CPC)	8	4	4.4	3.9	4.0	5	0
Functional Activities Questionnaire (FAQ)	8	0	4.9	4.4	4.4	5	1
Functional Performance Inventory (FPI)	4	0	4.0	3.4	3.3	16	0
Zubrod score	3	0	3.9	3.6	3.5	10	0
Disability Rating Scale (DRS)	3	0	3.8	3.6	3.5	11	0
QOL	(number)	(number)	(score)	(score)	(score)	(number)	(number)
Short Form-36 Including RAND-36 (SF-36)	153	42	8.1	8.1	8.2	0	23
EuroQol-5Dimension-5Level (EQ-5D-5L)	70	15	8.5	8.6	8.5	0	23
EuroQol-5Dimension-3Level (EQ-5D-3L)	46	8	7.3	7.1	7.0	0	18
EuroQol-Visual Analog Scale (EQ-VAS)	39	4	7.5	7.5	7.4	0	21
Short Form-12 (SF-12)	26	1	6.8	6.7	7.3	0	21
World Health Organization Quality of Life (WHOQOL)	5	0	5.2	5.0	5.1	3	3
Quality-Adjusted Life Year (QALY)	4	0	4.6	4.3	4.3	6	0
Assessment of Quality of Life-4D (AQoL-4D)	4	0	4.4	4.1	4.0	5	0
15D instrument	3	0	4.3	4.0	4.0	6	0
Sleep	(number)	(number)	(score)	(score)	(score)	(number)	(number)
Pittsburgh Sleep Quality Index (PSQI)	9	0	6.9	6.7	6.7	0	17
Insomnia severity index	8	0	6.3	6.0	6.0	0	5
Actigraphy	3	0	3.9	3.7	3.7	10	0
Pain	(number)	(number)	(score)	(score)	(score)	(number)	(number)
Brief Pain Inventory	13	0	7.1	7.0	7.1	0	19
NRS	5	2	6.7	6.3	6.6	0	14
VAS	3	0	6.8	6.3	6.5	0	14
Graded Chronic Pain Scale (GCPS)	3	0	5.0	4.7	4.8	3	2
Fatigue	(number)	(number)	(score)	(score)	(score)	(number)	(number)
Fatigue Severity Scale (FSS)	3	0	5.9	5.3	5.6	0	4
Fatigue Assessment Scale (FAS)	3	0	5.8	5.3	5.6	0	5
Others	(number)	(number)	(score)	(score)	(score)	(number)	(number)
Measure of Current Status part A (MOCS-A)	4	0	4.0	3.6	3.6	12	0
General Self Efficacy (GSE) scale	3	0	4.2	3.7	3.9	7	0
Family	(number)	(number)	(score)	(score)	(score)	(number)	(number)
SF-36	47	0	7.7	7.9	8.0	0	22
HADS	34	17	8.3	8.5	8.6	0	23
IES-R	19	0	8.2	8.3	8.6	0	23
Family Satisfaction in the ICU (FS-ICU)	10	1	6.3	6.4	6.4	0	13
PCL-S	7	0	5.8	5.6	5.5	0	2
IES	7	0	4.6	4.4	4.4	6	0
PCL-C	5	0	5.2	5.2	5.1	3	1
PHQ-9	5	0	6.0	6.3	6.0	1	8
CES-D	5	0	4.4	4.1	4.2	7	0
PSQI	5	0	6.0	5.8	6.0	1	8
Quality of Death and Dying (QODD)	3	0	4.5	4.4	4.4	2	0
PHQ-8	3	0	5.0	4.6	4.7	4	1
Zarit Burden Interview-12 items (Zarit-12)	3	0	4.8	4.8	4.9	2	2

Pulmonary function includes Spirometer, DLCO (Diffusing capacity of Lung for Carbon monOxide)

### The Delphi consensus

The results of 3 rounds of the modified Delphi consensus are shown in Table 1. We ultimately included 20 PICS assessment instruments (Table 2, [19–54]): (1) 3 items in physical function: the 6-min walk test, Medical Research Council (MRC) score, and grip strength, (2) 3 items in cognitive function: the Montreal Cognitive Assessment (MoCA), Mini-Mental State Examination (MMSE), and Short Memory Questionnaire (SMQ), (3) 3 items in mental health: the Hospital Anxiety and Depression Scale (HADS), Impact of Event Scale-Revised (IES-R), and Patient Health Questionnaire-9 (PHQ-9), (4) 3 items in ADL: the Barthel Index, Instrumental Activities of Daily Living (IADL), and Functional Independence Measure (FIM), (5) 5 items in QOL: Short Form-36 (SF-36), EQ-5D-5L (EuroQol-5Dimension-5Level), EQ-5D-3L (EuroQol-5Dimension-3Level), EQ-VAS (EuroQol-Visual Analog Scale), and SF-12, (6) 1 item in sleep: the Pittsburgh Sleep Quality Index (PSQI), 1 item in pain: Brief Pain Inventory, and (7) 3 items in family: SF-36, HADS, and IES-R.

**Table 2:** Summary of assessment instruments for post-intensive care syndrome

Domain	Assessment instruments	Items	Score rage	Reliability	Validity	MICD	Features
Physical	6-min walk test	1	–	0.72–0.99 [19]^a^	0.59 [20]	10% [21]	The value depends on age, sex, body weight, and height
	MRC (Medical Research Council) score	12	0–60	0.83–0.99 [22]	0.64 [23]	–	Muscle strength by manual muscle strength at 12 points
	Grip Strength	1	–	0.87–0.92 [24]	0.76 [25]	5.7–12.5 [24]	Muscle strength by a grip dynamometer
Cognitive	Montreal Cognitive Assessment (MoCA)	8	0–30	0.92 [26]	0.87 [26]	2 [27]	Visuospatial/executive, naming, memory, attention, language, abstraction, delayed recall, orientation
	Mini-Mental State Examination ( MMSE)	11	0–30	0.56–0.93 [28]	0.43–0.99 [29]	1–3 [30]^b^	Registration, attention, calculation, recall, language, ability to follow simple commands, orientation
	Short Memory Questionnaire ( SMQ)	14	4–46	–	–	–	Short-term memory, remote memory, cognition, orientation, calculation
Mental	Hospital Anxiety and Depression Scale (HADS)	14	0–21	0.86–0.90 [31]^c^	0.88–0.93 [32]	1.5 [33]^c^	Anxiety/Depression
	Impact of Event Scale-Revised (IES-R)	22	0–4 (average)	0.86 [34]	–	4.0 [35]	PTSD
	Patient Health Questionnaire-9 (PHQ-9)	9	0–27	0.84–0.89 [36]	0.73 [36]	5 [37]	Depression
ADL	Barthel Index	10	0–100	0.89–0.97 [38]	0.57–0.88 [39]	1.85 [40]^d^	Feeding, bathing, grooming, dressing, bowels, bladder, toilet, transfer, mobility, stairs
	Instrumental Activities of Daily Living (IADL)	8	0–8	0.92 [41]	0.26–0.84 [41]	–	Telephone, shopping, preparing food, housekeeping, laundry, transportation, medication, finance
	Functional Independence Measure (FIM)	13	13–91	0.83 [42]	–0.907 [42]	44 [43]	Self-care, toilet, transfer, locomotion, communication, social
QOL	Short Form-36 ( SF-36)	36	0–100	0.63–0.81 [44]	0.24–0.61 [45]^e^	2–6 [46]	Usage fee required, physical, pain, general health, vitality, social, emotional, mental
	EQ-5D-5L, 5D-3L, VAS	5	0–1	0.52–0.93 [47]	0.38–0.75 [48]	0.06–0.08 [49]	Mobility, self-care, usual activities, pain/discomfort, anxiety/depression
	SF-12	12	0–100	0.77–0.89 [50]	0.43–0.93 [50]	–	Usage fee required, physical, pain, general health, vitality, social, emotional, mental
Sleep	Pittsburgh Sleep Quality Index (PSQI)	9	0–21	0.86 [51]^f^	0.80 [52]^g^	4.4 [53]^h^	Sleep quality, latency, duration, efficiency, disturbance, medication, daytime sleep dysfunction
Pain	Brief Pain Inventory	9, 32	1–10	0.80 [54]	–	–	General, mood, walking, work, social, sleep, enjoyment
Family	SF-36	36	0–100	–	–	–	QOL
	HADS	14	0–14	–	–	–	Anxiety/depression
	IES-R	22	0–4 (average)	–	–	–	PTSD

Reliability was shown with intraclass and interclass correlation coefficients, and validity as a correlation with the standard evaluation. Blanks indicate insufficient information for the data. MICD: minimally important clinical difference, EQ-5D-5L, 5D-3L, VAS: EuroQol-5Dimension-5Level, 5Dimension-3Level, Visual Analog Scale, PTSD: post-traumatic stress disorder, QOL: quality of life

^a^Study related to chronic respiratory disease, ^b^ study related to Alzheimer’s disease, ^c^ study related to chronic obstructive pulmonary disease, ^d^ study related to stroke, ^e^ study related to brain tumor, ^f^ study related to temporomandibular disorder, ^g^ study related to healthy workers, ^h^ study related to rotator cuff tear repair

## Discussion

We herein conducted a scoping review and used the modified Delphi method to reach a consensus on recommendations of PICS assessment instruments. In the scoping review, 107 PICS assessment instruments were identified from the order of instrument use frequency between 2014 and 2022. After the modified Delphi meeting, we reached a consensus on 20 PICS assessment instruments for the physical, cognitive, mental, ADL, QOL, sleep, pain, and family domains. Three assessment instruments were included in each physical, cognitive, mental, ADL, QOL, and PICS-F assessment.

The assessment of physical function included the 6-min walk test, MRC score, and grip strength. The 6-min walk test was included as a PICS assessment instrument because of its confirmed validity [55, 56]; it is included in the Society of Critical Care Medicine’s consensus [5]. The advantage of using the MRC score is its convenience without the need for instruments. It is a valid and reliable tool for assessing prolonged physical impairments under careful training [57]. Grip strength allows for a quick evaluation of muscle strength and only requires a grip dynamometer. It is important to note that grip strength reflects not only grip strength, but also the whole-body strength and QOL of patients [58, 59].

Cognitive function assessments included MoCA, MMSE, and SMQ. MoCA is the most widely used for cognitive assessments, with several international consensuses [5, 60]. MMSE is often used in PICS assessments [61, 62], but may require the proper selection of a follow-up population [63]. Although SMQ was not included in previous international consensuses [5, 60], there are several positive features for its use. SMQ is a valid and reliable tool that correlates with MMSE [64]. It may also be assessed through a telephone interview or questionnaire [2], which is important for the continued follow-up of critically ill patients [65].

Mental health assessments included HADS, IES-R, and PHQ-9. HADS had the highest score in the modified Delphi meeting for anxiety and depression assessments, which is consistent with previous international consensuses [5, 60]. IES-R is used to assess PTSD [66], and is valid for evaluations of acute lung injury survivors [67]. IES-R is also recommended in the Society of Critical Care Medicine’s consensus with a clear cutoff value threshold [5]. PHQ-9 is the tool recommended by the American Heart Association to evaluate depression [36, 68] and has also been used in prolonged depression symptom assessments after COVID-19 [69]. IES-6 was not included as a recommendation in this study, but it was in the Society of Critical Care Medicine’s consensus. The infrequent use of IES-6 may have contributed to this difference.

The assessment of ADL included the Barthel Index, IADL, and FIM. In this study, the Barthel index was the most widely used scale for the ADL assessment [70]. IADL evaluates more complex skills in daily life. In a previous study, only 13% of survivors of critical illness were able to drive after hospital discharge [71]. These high-level skills are important for reintegration into society, as assessed by IADL. FIM includes communication and social cognitive functions [72]. The assessment of FIM is useful for monitoring changes in the status of a patient [73].

The assessment of QOL included SF-36, EQ-5D-5L, 3L, VAS, and SF-12. Although SF-36 involves questions on many items and requires a usage fee, it is the most widely used tool to assess the QOL of patients, reflecting the reliability and validity of this score [74]. SF-12 was included as a recommendation in this study. SF-12 is brief, but not inferior to SF-36 [75, 76]. Since numerous questionnaires may be stressful for patients [77], the brief version will be useful for future clinical use. EQ-5D was included as a recommendation, similar to a previous consensus [5]. The assessment of EQ-5D had a high score in the Delphi meeting in the following order: 5 levels, visual analog scale, 3 levels.

Other domains included sleep and pain. Many ICU survivors develop sleep disturbance and approximately 40% have chronic pain after hospital discharge [78, 79]. Therefore, these assessments are important. The assessment of sleep disturbance using PSQI [80] and pain with the Brief Pain Inventory [81] were previously recommended. It is important to note that the assessment of pain is also included in other QOL scores. These domains have been attracting increasing attention as symptoms included in PICS [82]. The management of sleep and pain symptoms warrants further study [83].

Family members of ICU survivors may also develop prolonged mental illness [84]. Family members experience anxiety, depression, and PTSD, which decrease QOL [85]. In the assessment of PICS-F, we recommend the use of HADS for anxiety and depression, IES-R for PTSD, and SF-36 for QOL. Few consensuses have been reported regarding PICS-F assessment tools [86]. Assessment tools varied among previous studies [87]. Therefore, this recommendation will contribute to the mental health issues and QOL of family members as well as ICU survivors.

Among 20 PICS assessment instruments, the following instruments had the highest scores in the final round of the modified Delphi consensus process in each domain of PICS-F: the MRC score in physical function, MoCA in cognitive function, HADS in mental health, the Barthel index in ADL, EQ-5D-5L in QOL, PSQI in sleep, Brief Pain Inventory in pain, and HADS in mental health. Because we finally identified 3 PICS assessment instruments in each domain, the single recommendation based on the highest scores may be more helpful for the PICS assessment in the future research. However, it is important to be aware that each instrument has different advantages and disadvantages and difficult to recommend a single assessment tool for each PICS domain.

Our final goal of providing PICS assessment instruments is the complete reintegration of ICU survivors into society. PICS may result in financial hardship, social isolation, suicide, and unemployment. As the first step for prevention, early identification is mandatory using these PICS assessment instruments. Furthermore, we need to provide continuous assessments and necessary interventions, including rehabilitation, nutrition management, and cognitive and psychological interventions. Although further studies are needed to validate these instruments for the assessment of PICS, these recommendations will contribute to preventing and managing PICS in ICU survivors and family members for their future reintegration into society.

### Limitations

This study has several limitations. The scoping review and Delphi consensus were conducted in Japan. Although generalizability may be limited, we did not consider convenience for use of the Delphi consensus in Japan in order to maintain its validation in other countries. Furthermore, some recommendations were consistent with previous consensuses [5, 8]. Another limitation is that Delphi meeting members did not include patients, their family, or various health care providers; however, we included physiotherapists and nurses as well as physicians. Moreover, since we did not include sleep or pain in the scoping review formula, their frequency of use may have been underestimated. We also conducted the modified Delphi method on scores used more than two times. Therefore, new scores used less than three times may not have been included in the present study. Recent scores, such as PCL-5, were not included in this consensus study partly due to their infrequent use in the scoping review. In addition the classification of instruments was difficult. We classified EQ-5D-5L, EQ-5D-3L, and EQ-5D-VAS as different scores, but did not divide the brief pain inventory into a short- or full-length version. This type of classification may have affected the results of the scoping review.

## Conclusions

We conducted a scoping review and the modified Delphi method to clarify the recommendations of instruments to assess PICS. Based on the results obtained, we recommended 20 PICS assessment instruments in the physical, cognitive, mental, ADL, QOL, sleep, pain, and family (PICS-F) domains. Further studies are needed to validate these instruments for PICS assessments.

### Supplementary Information

**Additional file 1. 1**. Search strategy, **2**. Summarized materials used in Delphi meeting, **3**. The study included in scoping review, **4**. Results of the scoping review at hospital discharge, **5**. Results of the scoping review after hospital discharge. **Table S1**. Reference list of 754 included studies in scoping review. **Table S2**. Extracted PICS assessment at hospital discharge. **Table S3**. Extracted PICS assessment after hospital discharge.

## Data Availability

Data are available upon reasonable request to the corresponding author.
