# Supplementary material for: Instruments to assess post-intensive care syndrome assessment: a scoping review and modified Delphi method study
Source: Crit Care. 2023 Nov 7;27:430. doi: 10.1186/s13054-023-04681-6 (PMC10629074; doi:10.1186/s13054-023-04681-6)
Supplement: Supplementary file 1 — Additional file 1. 1. Search strategy, 2. Summarized materials used in Delphi meeting, 3. The study included in scoping review, 4. Results of the scoping review at hospital discharge, 5. Results of the scoping review after hospital discharge. Table S1. Reference list of 754 included studies in scoping review. Table S2. Extracted PICS assessment at hospital discharge. Table S3. Extracted PICS assessment after hospital discharge. [file 13054_2023_4681_MOESM1_ESM.docx]

**1. Search strategy**

**Databases**

- MEDLINE
- CENTRAL
- CINAHL

Among 6972 identified records, 5160 were included after the deletion of duplicates.

MEDLINE (via PubMed) search strategy (Searched in AM–08:10, November 27, 2022)

|  | Search formula | Results |
| --- | --- | --- |
| #1 | (intensive care[tiab] OR ICU[tiab] OR ICUs[tiab] OR "intensive care units"[MeSH Terms] OR intensive therapy[tiab] OR critical care[tiab] OR "critical care"[MeSH Terms]) AND (survivors[tiab] OR postintensive care syndrome[tiab] OR post intensive care syndrome[tiab] OR PICS[tiab] OR family[tiab]) AND (impairment[tiab] OR physical[tiab] OR cognitive[tiab] OR mental[tiab] OR posttraumatic stress disorder[tiab] OR depression[tiab] OR anxiety[tiab] OR “activities of daily living"[MeSH Terms] OR ADL[tiab] OR quality of life[tiab] OR "quality of life"[MeSH Terms] OR morbidity[tiab] OR "morbidity"[MeSH Terms]) NOT (animals[mh] NOT humans[mh]) | 5835 |
| #2 | Limits: 2014/Jan/1 – present | 3471 |

Cochrane Central Register of Controlled Trials search strategy (Searched in A–08:46, November 27, 2022)

| #1 | ("intensive care"):ti,ab,kw | 27771 |
| --- | --- | --- |
| #2 | ("ICU"):ti,ab,kw | 16086 |
| #3 | ("ICUs"):ti,ab,kw | 1744 |
| #4 | MeSH descriptor: [Intensive Care Units] explode all trees | 4145 |
| #5 | ("intensive therapy"):ti,ab,kw | 1061 |
| #6 | ("critical care"):ti,ab,kw | 4519 |
| #7 | MeSH descriptor: [Critical Care] explode all trees | 2238 |
| #8 | #1 OR #2 OR #3 OR #4 OR #5 OR #6 OR #7 | 37647 |
| #9 | ("survivors"):ti,ab,kw | 12622 |
| #10 | ("postintensive care syndrome"):ti,ab,kw | 54 |
| #11 | ("post intensive care syndrome*"):ti,ab,kw | 70 |
| #12 | ("PICS"):ti,ab,kw | 117 |
| #13 | ("family"):ti,ab,kw | 39219 |
| #14 | #9 OR #10 OR #11 OR #12 OR #13 | 51355 |
| #15 | ("impairment"):ti,ab,kw | 38293 |
| #16 | ("physical"):ti,ab,kw | 142628 |
| #17 | ("cognitive"):ti,ab,kw | 84978 |
| #18 | ("mental"):ti,ab,kw | 70589 |
| #19 | ("posttraumatic stress disorder"):ti,ab,kw | 5517 |
| #20 | (“depression”):ti,ab,kw | 90609 |
| #21 | ("anxiety"):ti,ab,kw | 64582 |
| #22 | MeSH descriptor: [Activities of Daily Living] explode all trees | 10374 |
| #23 | ("ADL"):ti,ab,kw | 4159 |
| #24 | ("quality of life"):ti,ab,kw | 135560 |
| #25 | MeSH descriptor: [Quality of Life] explode all trees | 29546 |
| #26 | ("morbidity"):ti,ab,kw | 43154 |
| #27 | MeSH descriptor: [Morbidity] explode all trees | 15984 |
| #28 | #15 OR #16 OR #17 OR #18 OR #19 OR #20 OR #21 OR #22 OR #23 OR #24 OR #25 OR #26 OR #27 | 471247 |
| #32 | #8 AND #14 AND #28 | 1112 |
| #33 | Limits: trial | 1087 |
| #34 | Limits: 2014/Jan/1 – present | 823 |

CINAHL (Searched in AM–9:17, November 27, 2022)

| #1 | ("intensive care" OR "ICU" OR "ICUs" OR MH "intensive care units" OR "intensive therapy" OR "critical care" OR MH "critical care") AND ("survivors" OR "postintensive care syndrome" OR "post intensive care syndrome" OR "PICS" OR "family") AND ("impairment" OR "physical" OR "cognitive" OR "mental" OR "posttraumatic stress disorder" OR "depression" OR "anxiety" OR MH "activities of daily living" OR "ADL" OR "quality of life" OR MH "quality of life" OR "morbidity" OR MH "morbidity") | 4315 |
| --- | --- | --- |
| #2 | Limits: 2014/Jan/1 – present | 2678 |

**2. Summarized materials used in Delphi meeting**

Physical Function Measurement Tests

- 6-Minute Walk Test
- Pulmonary Function Test, DLCO
- MRC Score
- Grip Strength
- Clinical Frailty Scale (CFS)
- Sit-to-stand
- Short Physical Performance Battery (SPPB)
- 4-m Gait Speed Test
- Manual Muscle Test (MMT)
- Medical Resource Council (MRC) Dyspnea Scale
- Saint George’s Respiratory Questionnaire (SGRQ)
- Quadriceps Muscle Strength
- Timed Up and Go Test (TUG)
- BERG Balance Test
- Physical Functional Status (PFS)
- Chelsea Critical Care Physical Assessment Tool (CPAx)
- Borg Dyspnea Scale
- 10-minute Walking Test
- 2-minute Walking Test
- Fried Frailty Criteria
- PFIT: Physical Functional Test for the ICU
- Functional Status Score for the ICU (FSS-ICU)
- Functional Ambulation Categories (FAC)

6-Minute Walk Test

1. Characteristics

The 6-minute walk test evaluates exercise tolerance by walking for 6 minutes. It was reported in 1982 by Butland et al. [1]. In 2002, guidelines were released by the American Thoracic Society (ATS), proposing a standardized method [2]. In 2014, a systematic review and technical standards were announced jointly by ATS/European Respiratory Society [3].

2. Procedure and Method

A subject walks towards marker cones set up around the measurement area. It is possible to take breaks during the walk. During the measurement, the subject is observed and encouraged verbally to walk as far as possible, aiming for the maximum walking distance.

The results of the 6-minute walk test vary depending on factors such as the subject’s age, sex, weight, and height. A short distance in this test (300–450 m) strongly correlates with a high mortality rate, and the prognosis of patients is poor if the distance is less than 300 m.

3. Other Information

There is no fee for using the 6-minute walk test. The intraclass correlation was 0.72–0.99 for chronic respiratory disease [4]. Validity was r = 0.59 [5]. The minimally important clinical difference was 10% or 35 m (95% CI, 30–42) [6].

References

[1] Butland RJ, Pang J, Gross ER, Woodcock AA. Two-, six-, and 12-minute walking tests in respiratory disease. Br Med J (Clin Res Ed). 1982;284(6329):1607-1608.

[2] ATS Committee on Proficiency Standards for Clinical Pulmonary Function Laboratories. ATS statement: guidelines for the six-minute walk test. Am J Respir Crit Care Med. 2002;166(1):111-117.

[3] Holland AE, Spruit MA, Troosters T, Puhan MA. An official European Respiratory Society/American Thoracic Society technical standard: field walking tests in chronic respiratory disease. Eur Respir J. 2014 Dec;44(6):1428-46.

[4] Singh SJ, Puhan MA, Andrianopoulos V, Hernandes NA, Mitchell KE, Hill CJ, Lee AL, Camillo CA, Troosters T, Spruit MA. An official systematic review of the European Respiratory Society/American Thoracic Society: measurement properties of field walking tests in chronic respiratory disease. European Respiratory Journal 2014;44(6):1447-78.

[5] Alison JA, Kenny P, King MT, McKinley S, Aitken LM, Leslie GD, Elliott D. Repeatability of the six-minute walk test and relation to physical function in survivors of a critical illness. Phys Ther 2012;92(12):1556-63.

[6] Puhan MA, Mador MJ, Held U, Goldstein R, Guyatt GH, Schünemann HJ. Interpretation of treatment changes in 6-minute walk distance in patients with COPD. Eur Respir J 2008;32(3):637-43.

Pulmonary Function Test, DLCO

1. Characteristics

The spirometer, one of the most common respiratory function tests, is a method to measure the function of the lungs. DLCO is a respiratory function test that assesses the diffusion capacity of the lungs.

2. Procedure and Method

The device measures the flow of air from the exhalation port. The patient places their mouth on this device, takes a deep breath, and then exhales with a constant force. By measuring the volume and speed of exhaled air, the function of the lungs may be evaluated. With the spirometer test, indicators, such as lung capacity, Forced Expiratory Volume in the 1st second (FEV1), inhalation strength, and exhalation strength, may be obtained. Evaluations of these indicators reveal the extent of pulmonary dysfunction and contribute to diagnosing the type of respiratory disease.

To measure DLCO, a mixed gas with a small amount of carbon monoxide (CO) is inhaled, followed by maximal exhalation, maximal inhalation, and breath-holding for 10 seconds. During breath-holding, gas diffuses from the alveoli and the amount of CO in the inhaled mixed gas decreases. After 10 seconds, a maximal exhalation is performed and the amount of CO in collected exhaled air is measured.

Commonly used cut-off indicators include:

• FEV1: Less than 70% of the predicted value

• Forced Vital Capacity (FVC): Less than 70% of the predicted value

• FEV1/FVC ratio: Less than 70%

• Maximum Mid-Expiratory Flow: Less than 65% of the predicted value

The standard range of DLCO values is generally considered to be 80-120% of the predicted value. If it is less than 70%, the gas exchange ability of the lungs may be impaired.

3. Other Information

There is no fee for using this test.

MRC (Medical Research Council) score

1. Characteristics

The MRC score is one of the scores used to evaluate muscle strength using the manual muscle test (MMT). The MRC proposed this scale. Muscle strength may be continuously evaluated with the MRC score not only during ICU admission, but also after ICU discharge [1].

2. Procedure and Method

The MRC score is used to evaluate the gradual decline in muscle strength. It is assessed in 6 stages from 0 to 5, where 0 indicates no visible muscle contraction and 5 indicates normal muscle strength.

In the MRC score, the upper limbs are examined for shoulder abduction, elbow flexion, and hand extension, while the lower limbs are assessed for hip flexion, knee extension, and foot dorsiflexion. They are assessed at 6 points on both the left and right sides, providing a total of 12 sites. The score ranges from 0 to a maximum of 60 points.

Six-step evaluation of the MRC Score:

0: No muscle contraction

1: Muscle contraction present, but only capable of movement under the force of gravity

2: May overcome gravity in movement, but has insufficient strength

3: May overcome gravity in movement and core with added resistance from gravity, but cannot handle any more resistance

4: May overcome gravity and cope with resistance from weight as well as slight resistance, but cannot handle more than that

5: Normal muscle strength

3. Other Information

There is no fee for using the test. The intraclass correlation coefficient was 0.83–0.99 and Pearson’s product moment correlation coefficient was 0.96 [2]. The correlation between grip strength and the MRC score was Spearman’s rank correlation coefficient of 0.643 [3].

References

[1] Turan Z, Topaloglu M, Ozyemisci Taskiran O. Medical research council-sumscore: a tool for evaluating muscle weakness in patients with post-intensive care syndrome. Crit Care. 2020 Sep 18;24(1):562.

[2] Vanpee G, Hermans G, Segers J, Gosselink R. Assessment of limb muscle strength in critically ill patients: a systematic review. Crit Care Med. 2014;42(3):701-11

[3] Ali NA, O'Brien JM, Jr., Hoffmann SP, Phillips G, Garland A, Finley JC. Acquired weakness, handgrip strength, and mortality in critically ill patients. American journal of respiratory and critical care medicine. 2008;178(3):261-8.

Grip Strength

1. Characteristics

While grip strength is primarily measured in a standing position, many patients in the ICU cannot stand for measurements. Reliable values may be obtained even in a seated position or on a bed by bending the elbow to 90 degrees during the measurement [1]. Severely ill patients admitted to the ICU often experience muscle atrophy not only in the lower limbs, but also in the upper limbs, resulting in reduced grip strength [2]. Some facilities that conduct patient follow-ups in Post-Intensive Care Syndrome (PICS) outpatient clinics measure grip strength, and previous findings showed that grip strength was related to mental function impairments and quality of life (QOL) in PICS [3].

2. Procedure and Method

A patient’s arm is extended at their side, and they are asked to grip a dynamometer. The width of the grip on the dynamometer is adjusted so that the second joint of the index finger is at an approximately 90-degree angle (almost a right angle). Grip strength is measured alternately on the left and right sides twice, and values are rounded down to the nearest kilogram. The average of the better result from each hand is taken.

Cut-off values

Criteria for sarcopenia [4]: Men: Less than 28 kg, Women: Less than 18 kg.

3. Other Information

There is no fee for using the test. Reliability showed an intraclass correlation coefficient of 0.866–0.922 [5]. Validity was r of 0.763 [6]. Internal consistency was 0.925–0.946 in critically ill patients [5]. Minimal clinically important differences were 7.8 kg (right) and 12.5 kg (left) in critically ill patients [5].

References

[1] Sousa-Santos AR, Amaral TF. Differences in handgrip strength protocols to identify sarcopenia and frailty - a systematic review. BMC geriatrics. 2017;17(1):238.

[2] Nakanishi N, Oto J, Tsutsumi R, Akimoto Y. Upper limb muscle atrophy associated with in-hospital mortality and physical function impairments in mechanically ventilated critically ill adults: a two-center prospective observational study. Journal of Intensive Care. 2020;8(1):87.

[3] Nakamura K, Kawasaki A, Suzuki N, Hosoi S. Grip strength correlates with mental health and quality of life after critical care: a retrospective study in a post-intensive care syndrome clinic. J Clin Med. 2021;Jul 8;10(14):3044.

[4] Japan Society of Sarcopenia and Frailty. Clinical practice guidelines for sarcopenia. Japanese Journal of Geriatrics. 2019;(56)3:217

[5] Baldwin CE, Paratz JD, Bersten AD. Muscle strength assessment in critically ill patients with handheld dynamometry: an investigation of reliability, minimal detectable change, and time to peak force generation. J Crit Care 2013;28(1):77-86.

[6] Nordon-Craft A, Schenkman M, Edbrooke L, Malone DJ, Moss M, Denehy L. The physical function intensive care test: implementation in survivors of critical illness. Phys Ther 2014; 94(10):1499-507.

Clinical Frailty Scale (CFS)

1. Characteristics

The CFS was proposed by Rockwood K and others in 2005 to evaluate the degree of frailty in elderly individuals. It comprehensively assesses various symptoms and disabilities related to the decline in physical function in the elderly, evaluating the degree of the impact on their physical function.

2. Procedure and Method

The CFS evaluates frailty in nine stages, from 1 to 9. A score of 1 indicates good health, while a score of 9 reflects very severe frailty. The CFS assessment is relatively simple and quick, making it useful for diagnosing elderly patients and planning their treatment [1].

3. Other Information

The copyright for the CFS belongs to Professor Rockwood. No specific permission is required for its use for personal and non-commercial purposes. However, permission may need to be obtained from Professor Rockwood for commercial purposes. The intraclass correlation was good for both inter-rater (0.87, 95% CI: 0.82–0.90) and test-retest reliabilities (0.89: 95% CI: 0.85–0.92). Validity between CFS and the Barthel Index was r of − 0.725 [2].

References

[1] Rockwood K, Song X, MacKnight C, Bergman H. A global clinical measure of fitness and frailty in elderly people. CMAJ. 2005;Aug 30;173(5):489-95.

[2] Vrettos I, Voukelatou P, Panayiotou S, Kyvetos A, Kalliakmanis A, Makrilakis K, Sfikakis PP, Niakas D. Validation of the revised 9-scale clinical frailty scale (CFS) in Greek language. BMC Geriatr 2021;21(1):393.

Sit-to-stand test

1. Characteristics

The sit-to-stand test is used to measure the action of standing up from a sitting position and then sitting back down. Due to aging or diseases that decrease muscle strength or balance, some individuals may find this motion challenging.

2. Procedure and Method

The sit-to-stand test is a simple functional test in which the patient is asked to stand up from a chair, remain standing for a few seconds, and then sit back down. Another method of measuring involves counting how many times the individual stands up and sits down within a 30-second period. A correlation has been observed between this test and the 50-foot walk test [1].

3. Other Information

There is no fee for using the test. In ICU survivors, inter- (ICC 0.92 [95% CI 0.89–0.94]) and intra-rater (ICC 0.95 [95% CI 0.93–0.96]) reliabilities were both excellent and higher scores were associated with lower muscle strength, a longer hospital stay, and greater functional impairment at hospital discharge [2].

References

[1] Gill SD, de Morton NA, Mc Burney H. An investigation of the validity of six measures of physical function in people awaiting joint replacement surgery of the hip or knee. Clinical rehabilitation. 2012;Oct;26(10):945-51.

[2] de Melo TA, Silva Guimarães F, Lapa e Silva JR. The five times sit-to-stand test: safety, validity and reliability with critical care survivors’s at ICU discharge. Archives of Physiotherapy 2022;13(1):2.

Short Physical Performance Battery (SPPB)

1. Characteristics

The SPPB was developed by the National Institute on Aging (NIA) for the purpose of evaluating lower limb function in the elderly and was introduced in 1994. In recent years, it has also been used as one of the diagnostic criteria for sarcopenia by the European Working Group on Sarcopenia in Older People (EWGSOP).

2. Procedure and Method

The total score for evaluation items is a maximum of 12 points, with a higher score indicating superior physical function. Scores of 0-6 are classified as low performance, 7-9 as standard performance, and 10-12 as high performance.

Guralnik et al. conducted a longitudinal study on the relationship between the SPPB test and physical functional impairment (limitations) [1]. In comparisons with the category showing the highest physical performance (total score of 10-12), a score of 7-9 was associated with twice the risk of a walking disability, while a score of 4-6 had five-fold the risk. Furthermore, the performance cut-off value used to classify sarcopenia is a score of 8 or lower in the SPPB test.

3. Other Information

There is no fee for using the SPPB. Test-retest reliability was high at 0.87, and validity was demonstrated by its relationship with measures of the health status and variables of functional capacity [2].

References

[1] Guralnik JM, Simonsick EM, Ferrucci L, Glynn RJ. A short physical performance battery assessing lower extremity function: association with self-reported disability and prediction of mortality and nursing home admission. J Gerontol. 1994 Mar;49(2):M85-94.

[2] Gómez JF, Curcio CL, Alvarado B, Zunzunegui MV, Guralnik J. Validity and reliability of the Short Physical Performance Battery (SPPB): a pilot study on mobility in the Colombian Andes. Colomb Med (Cali) 2013;44(3):165-71.

4-m Gait Speed Test

1. Characteristics

The 4-m gait speed test is a simple test used to measure walking speed.

2. Procedure and Method

A subject walks at their usual pace, and their walking speed is measured. Specifically, the subject is asked to walk a distance of 4 m, and the time it takes is recorded to assess the walking speed.

If more than 5 seconds is needed to walk 4 m (less than 0.8 m/s), a decrease in walking speed is indicated. This suggests a risk of frailty and the need for further clinical examinations.

3. Other Information

There is no fee for using this test. Correlations have been reported between the 4-m gait speed and 6-minute walk distance (r = 0.57; p <0.001) [1]. Multivariate analyses showed that the 4-m gait speed and 6-minute walk distance both correlated with the modified MRC score.

Reference

[1] Hirabayashi R, Takahashi Y, Nagata K, Morimoto T, Wakata K, Nakagawa A, Tachikawa R, Otsuka K, Tomii K. The validity and reliability of four-meter gait speed test for stable interstitial lung disease patients: the prospective study. J Thorac Dis 2020; 12(4):1296-304.

MMT

1. Characteristics

The MMT has been widely used in the clinical field as a convenient approach to evaluate a patient’s muscle strength since it was introduced by Lovett in 1916 [1]. MMT by Daniels et al. [2] is now widely used as an evaluation method to measure the strength and function of muscles. It is widely employed in the medical and rehabilitation fields. This test evaluates the resistance force generated when a patient contracts a specific muscle, thereby measuring the strength of the muscle. A patient typically changes their posture or moves parts of their body while contracting a specific muscle. The test administrator applies manual resistance to the patient’s muscle contraction to assess muscle strength.

2. Procedure and Method

Muscle strength is evaluated based on the amount of force the muscle may overcome against resistance. Strength is assessed using test scores or grades, typically rated on a 6-point scale from 0 to 5. Grade 0 indicates a lack of muscle strength, while Grade 5 indicates normal muscle strength.

Recording Results

It is crucial to document results appropriately and utilize them for subsequent follow-ups and treatment planning.

3. Other Information

There is no fee for using the MMT. The median percent agreement and intraclass correlation coefficient were found to be 0.96 (95% CI: 0.91 to 0.98) and 0.98 (95% CI: 0.95 to 1.00), respectively. ICC for the overall composite MMT score was 0.99 (95% CI: 0.98 to 1.00). Agreement (κ value; 95% CI) in detecting clinically significant weakness was 0.88 (0.44–1.00) [3].

References

[1] Lovett R.W, Martin EG. Certain aspects of infantile paralysis and a description of a method of muscle testing. JAMA, 1916:66 729-733

[2] Daniels L Williams M. Muscle testing: techniques of manual examination. Physical Therapy, 1947 Jan; 27: 1-39

[3] Fan E, Ciesla ND, Truong AD, Bhoopathi V, Zeger SL, Needham DM. Inter-rater reliability of manual muscle strength testing in ICU survivors and simulated patients. Intensive Care Med 2010;36(6):1038-43.

MRC Dyspnea Scale

1. Characteristics

The MRC dyspnea scale is one of the scales used to assess the severity of respiratory difficulties in respiratory diseases, such as chronic obstructive pulmonary disease (COPD). It was created based on the breathlessness scale by Fletcher [1].

2. Procedure and Method

Patients are presented with the following 5-grade evaluation scale and asked to choose that which most accurately describes their symptoms: There are also versions of the evaluation table without grade notation. An increase of two grades or more is generally considered to be a significant clinical change.

3. Other Information

Since the MRC dyspnea scale was developed by the MRC, it owns the copyright. However, the scale itself is commonly used and is freely available for use. Correlations have been reported between MRC scores and shuttle distance, St George’s Respiratory Questionnaire (SGRQ), the Chronic Respiratory Questionnaire, and Nottingham Extended Activities of Daily Living [2].

References

[1] Fletcher CM. The clinical diagnosis of pulmonary emphysema; an experimental study. Proc R Soc Med. 1952 Sep;45(9):577-84.

[2] Bestall JC, Paul EA, Garrod R, Garnham R, Jones PW, Wedzicha JA. Usefulness of the Medical Research Council (MRC) dyspnea scale as a measure of disability in patients with chronic obstructive pulmonary disease. Thorax 1999; 54(7):581-6.

SGRQ

1. Characteristics

SGRQ was proposed in 1991 by Paul Jones and colleagues [1]. It is a disease-specific questionnaire designed to measure respiratory symptoms, daily life limitations, and health-related QOL. This questionnaire is used to assess the health-related QOL of patients with respiratory diseases, such as asthma, COPD, and pulmonary fibrosis.

2. Procedure and Method

The questionnaire consists of 50 items and is divided into three components: Symptom (Symptoms), Activity (Activities), and Impact (Impacts). Additionally, it is possible to calculate a total score by summing the scores of these three components. Cut-off value: Not specified.

A decrease of 4 points is recognized as a significant difference in treatment or evaluation before and after.

3. Other Information

There is no fee for using SGRQ. It is a reliable and valid test of the QOL of patients with asthma, and scores were shown to correlate with symptom duration, lung function, and previous exacerbation history [2].

References

[1] Jones PW, Quirk FH, Baveystock CM. The St George's respiratory questionnaire. Respir Med. 1991 Sep;85 Suppl B:25-31; discussion 33-7.

[2] Kim T. Validity and Reliability of the St. George's Respiratory Questionnaire in Asthmatic Patients. Journal of Allergy and Clinical Immunology 2008; 121(2):S80.

Quadriceps Muscle Strength

1. Characteristics

Quadriceps muscle strength is an indicator that is used to measure the strength of the quadriceps femoris muscle. It is widely employed to evaluate lower limb strength and function, particularly impairments in daily activities due to muscle weakness, and also to assess rehabilitation.

2. Procedure and Method

A patient sits down and positions their knee by bending at a 90-degree angle. A strap is fixed to one of the patient’s ankles, and by pulling the strap, resistance is applied to the lower limb muscles. The patient is asked to exert their maximum force, and the peak value of this force is measured. The measurement is conducted three times, and the highest value is adopted.

Cut-off: Not specified.

3. Other Information

It requires a device for measurements.

Timed Up and Go Test (TUG)

1. Characteristics

The TUG was developed in 1986 by Mathias et al. [1] and was later modified in 1991 by Podsiadlo et al. [2] with the addition of a time element, making it a quantitative evaluation. The test has high intra-rater and inter-rater reliabilities. It has been reported to strongly correlate with lower limb strength, balance, walking ability, and activities of daily living (ADL).

2. Procedure and Method

The TUG is a functional test that evaluates how quickly and safely a subject stands up from a chair, walks a specified distance, and then sits down again. This test is often used for frailty checks based on physical functions, such as age, balance, walking speed, and muscle strength.

Cut-off values: Shumway-Cook [3] set the cut-off value at 13.5 seconds when comparing those who had experienced falls with those who had not. Bischoff [4] set the cut-off value at 12 seconds when comparing community-dwelling older adults with facility residents.

3. Other Information

There is no fee for using the TUG. ICC was good in elderly community-dwelling individuals and nursing home residents, and significant differences were reported between older adults [5].

References

[1] Mathias S, Nayak US, Isaacs B. Balance in elderly patients: the "get-up and go" test. Arch Phys Med Rehabil.1986 Jun;67(6):387-9.

[2] Podsiadlo D, Richardson S. The timed "Up & Go": a test of basic functional mobility for frail elderly persons. J Am Geriatr Soc.1991 Feb;39(2):142-8.

[3] A Shumway-Cook, S Brauer, M Woollacott. Predicting the probability for falls in community-dwelling older adults using the timed up & go test. Phys Ther. 2000 Sep;80(9):896-903.

[4] Bischoff HA, Stähelin HB, Monsch AU, Iversen MD. Identifying a cut-off point for normal mobility: a comparison of the timed 'up and go' test in community-dwelling and institutionalised elderly women. Age Ageing.2003 May;32(3):315-20.

[5] Rodrigues F, Teixeira JE, Forte P. The Reliability of the Timed Up and Go Test among Portuguese elderly. healthcare 2023;11(7):928.

BERG Balance Test

1. Characteristics

The BERG balance test was introduced in 1989 by Berg [1]. It is an evaluation battery designed to quantify the balance ability of older adults and patients who have had a stroke.

2. Procedure and Method

This test involves performing 14 predetermined tasks. Each task is scored from 0 to 4 based on an individual’s ability to complete the task, with a maximum score of 56. A score of 0 indicates an inability to perform the task, while 4 indicates that the task was fully performed. The total score ranges between 0 and 56, with higher scores indicating superior balance function.

The following cut-off scores were reported for older adults by Berg et al. in 1992 [2]: a score of 56 indicates functional balance, while a score less than 45 suggests a high risk of falls.

3. Other Information

The BERG balance test is copyrighted by its developer, Dr. Berg. Unauthorized duplication, modification, or commercial use is prohibited.

Usuda et al. [3] reported that within an elderly population, if a patient’s initial score falls within 45-56, a change of 4 points; within 35-44, a change of 5 points; within 25-34, a change of 7 points; or within 0-24, a change of 5 points, this reflects a genuine change with 95% confidence.

References

[1] Katherine B S, Wood-Dauphine J.I., Williams D Gayton. Measuring balance in the elderly : preliminary development of an instrument Physiotherapy Canada 41: 04-311, 1989.

[2] Berg KO, Wood-Dauphinee SL, Williams JI, Maki B. Measuring balance in the elderly: validation of an instrument. Canadian journal of public health= Revue canadienne de sante publique. 1992 Jul 1;83:S7-11.

[3] Usuda S, Araya K, Umehara K, Endo M, Shimizu T, Endo F. Construct validity of functional balance scale in stroke inpatients. J Phys Therapy Sci. 1998;10:53–6.

Physical Functional Status (PFS)

1. Characteristics

The PFS is one of the simple assessment scales of a patient’s physical function and allows for continuous evaluations of physical capability.

2. Procedure and Method

Respondents answer each question, indicating, for example, the distances they travel and the number of repetitions they perform. Responses may be in a Yes/No format or involve multiple-choice options depending on the question. Respondents are instructed to select the answer that most closely matches their abilities. Since the PFS is a self-reported measure of physical function, it relies on the subjective judgment of the respondent.

3. Other Information

There is no fee for using the PFS. It has been confirmed as a reliable scale with construct validity [1].

Reference

[1] Jette AM, Davies AR, Cleary PD, Calkins DR, Rubenstein LV, Fink A, Kosecoff J, Young RT, Brook RH, Delbanco TL. The Functional Status Questionnaire: reliability and validity when used in primary care. J Gen Intern Med 1986;1(3):143-9.

Chelsea Critical Care Physical Assessment Tool (CPAx)

1. Characteristics

The CPAx was created in 2013 by Corner et al. [1] as a scoring system to assess the physical function of critically ill patients. It evaluates a patient’s muscle strength, function, and mobility. The findings obtained may be beneficial for predicting a patient’s functional recovery and prognosis after discharge.

2. Procedure and Method

There are 10 items, each rated from level 0 to 5, resulting in a score range from 0 to 50. The 10 evaluation items include (ⅰ) respiratory function, (ⅱ) cough, (ⅲ) movements on the bed, (ⅳ) sitting at the edge of the bed, (ⅴ) maintaining an edge-sitting position, (ⅵ) standing, (ⅶ) maintaining standing, (ⅷ) moving to a chair, (ⅸ) foot tapping, and (ⅹ) grip strength. The assessment typically takes approximately 10 to 15 minutes.

If the CPAx score is 18 or higher at ICU discharge, the sensitivity for predicting home return within 90 days is 80% and specificity is 70% [2].

3. Other Information

There is no fee for using CPAx. It was shown to be a valid, reliable, and feasible tool for evaluating the physical functional state of COVID-19 patients following their discharge from the ICU [3].

References

[1] Corner EJ, Wood H, Englebretsen C, Thomas A. The Chelsea critical care physical assessment tool (CPAx): validation of an innovative new tool to measure physical morbidity in the general adult critical care population; an observational proof-of-concept pilot study. Physiotherapy 2013;99(1):33-41.

[2] Eggmann S, Verra ML, Stefanicki V, Kindler A. Predictive validity of the Chelsea critical care physical Assessment tool (CPAx) in critically ill, mechanically ventilated adults: a prospective clinimetric study. Disabil Rehabil. 2023 Jan;45(1):111-116.

[3] Giray E, Turan Z, Öke D, Topaloğlu M, Baygul A, Curci C, de Sire A, Taskiran OO. Validity, inter-rater reliability, and feasibility of the Chelsea Physical Assessment Tool for assessing physical function in post-acute COVID-19 patients: A cross-sectional study. J Back Musculoskelet Rehabil 2023;36(3):527-39.

Borg Dyspnea Scale

1. Characteristics

The Borg Dyspnea Scale is a subjective measure used to gauge the degree of breathlessness associated with physical activity or exertion. It was developed in the 1960s by a Swedish physician, Gunnar Borg. The Borg Scale is applied to patients with dyspnea during physical activity. Since patients perform a self-assessment, it is essential to be aware that the Borg Scale is subjective. Furthermore, it is not suitable for assessing a patient’s overall respiratory state because it only evaluates breathlessness during physical activities.

2. Procedure and Method

The Borg Scale utilizes numbers from 0 to 10, where 0 indicates no perceived breathlessness and 10 signifies the most severe shortness of breath. Patients self-assess the level of dyspnea they experience and select a numerical evaluation accordingly.

3. Other Information

This scale was developed by the Swedish physiologist Gunnar Borg and is copyrighted. However, it is now widely used and has been employed in many studies; therefore, it is generally available free of charge. It is generally permissible to use it for research purposes. The minimum important difference for the Borg fatigue scale was shown to be approximately 1 unit [1].

Reference

[1] Khair RM, Nwaneri C, Damico RL, Kolb T, Hassoun PM, Mathai SC. The Minimal Important Difference in Borg Dyspnea Score in Pulmonary Arterial Hypertension. Ann Am Thorac Soc 2016; 13(6):842-9.

10-minute Walking Test

1. Characteristics

The 10-minute walking test was designed to evaluate how far an individual walks, typically along a corridor, within a span of 10 minutes.

2. Procedure and Method

The test is conducted using a hospital corridor or a similar area. A participant walks for 10 minutes, and the distance covered is then measured. During the test, an examiner accompanies the participant, providing encouragement and urging them to put in their best walking effort. Concurrently, a pulse oximeter is used to measure SpO_2_ every minute.

3. Other Information

There is no fee for using the test. The intra-rater and inter-rater reliabilities of the 10-minute walking test were shown to be good (ICC between 0.76 until 0.9) and excellent (ICC >0.9), respectively [1]. The minimal detectable change for intra-rater reliability was 0.188 m/s, and it had moderate construct validity (r >0.5) in adolescents and young adults with Down syndrome.

Reference

[1] Sánchez-González JL, Llamas-Ramos I, Llamas-Ramos R, Molina-Rueda F, Carratalá-Tejada M, Cuesta-Gómez A. Reliability and Validity of the 10-Meter Walk Test (10MWT) in adolescents and young adults with down syndrome. Children 2023;10(4):655.

2-minute Walking Test

1. Characteristics

The 2-minute walking test serves as a standard assessment to evaluate an individual’s exercise tolerance, particularly when they are unable to perform the 6- or 12-minute walking test [1].

2. Procedure and Method

A participant is asked to walk as far as possible within a span of 2 minutes. The test is typically conducted either indoors or on an outdoor course, allowing the participant to walk at their own pace.

3. Other Information

There is no fee for using the test. Previous studies showed that the 2MWT was consistently reproducible [2-3].

Furthermore, it yielded findings that were consistent with those of the Berg Balance test, TUG, and the 6-minute walking test when administered concurrently to the same patient [4].

References

[1] Butland RJ, Pang J, Gross ER, Woodcock AA, Geddes DM. Two-, six-, and 12-minute walking tests in respiratory disease. Br Med J (Clin Res Ed). 1982. 29; 284(6329): 1607-1608.

[2] Selman JP, de Camargo AA, Santos J, Lanza FC. Reference equation for the 2-minute walk test in adults and the elderly. Respir Care. 2014 Apr;59(4):525-30.

[3] Bohannon RW, Wang YC, Gershon RC. Two-minute walk test performance by adults 18 to 85 years: normative values, reliability, and responsiveness. Arch Phys Med Rehab. 2015; 96:472-7.

[4] Connelly DM, Thomas BK, Cliffe SJ, Perry WM. Clinical utility of the 2-minute walk test for older adults living in long-term care. Physiother Can. 2009 Spring; 61(2): 78–87.

Fried Frailty Criteria

1. Characteristics

Fried and colleagues identified five factors that define frailty: weight loss, decreased muscle strength, fatigue, reduced walking speed, and low physical activity. According to their phenotype model [1], individuals with one or two of these criteria are considered to be “prefrail” (a stage before frailty), while those with three or more criteria are “frail”.

The criteria for grip strength and walking speed have been adjusted to fit the characteristics of the Japanese population [2]. In a cohort study of approximately 4,300 individuals [2], the prevalence of frailty was 6.9%. Furthermore, over a span of two years, a higher number of elderly individuals categorized as frail required care, thereby validating its predictive accuracy.

2. Procedure and Method

An individual is considered to be “frail” if they meet three or more of the criteria.

3. Other Information

There is no fee for using these criteria. Inter- and intra-rater compliance were found to be good [3].

References

[1] Fried LP, Tangen CM, Walston J, Newman AB. Frailty in older adults: evidence for a phenotype. J Gerontol A Biol Sci Med Sci.2001 Mar;56(3):M146-56.

[2] Makizako H, Shimada H, Doi T, Tsutsumimoto K. Impact of physical frailty on disability in community-dwelling older adults: a prospective cohort study. BMJ Open. 2015 Sep 2;5(9):e008462.

[3] Varan HD, Deniz O, Çöteli S, Doğrul RT, Kızılarslanoğlu MC, Göker B. Validity and reliability of Fried frailty phenotype in Turkish population. Turk J Med Sci 2022;52(2):524-7.

Physical Functional Test for the ICU (PFIT)

1. Characteristics

The PFIT is used to evaluate the physical function and mobility of patients in the ICU. It investigates whether a patient has the capability to perform ADL, such as sitting, standing, walking, and transferring from the bed.

2. Procedure and Method

If the test is performed multiple times, the highest score is adopted.

3. Other Information

There is no fee for using the PFIT. It showed moderate convergent validity with the TUG (r = -0.60), six-minute walk test (r = 0.41), and MRC summed score (rho = 0.49) [1]. A higher admission PFIT score was predictive of a MRC score ≥48, an increased likelihood of discharge home, a reduced likelihood of discharge to inpatient rehabilitation, and a reduced acute care hospital length of stay.

Reference

[1] Denehy L, de Morton NA, Skinner EH, Edbrooke L, Haines K, Warrillow S, Berney S. A physical function test for use in the intensive care unit: validity, responsiveness, and predictive utility of the physical function ICU test (scored). Phys Ther 2013;93(12):1636-45.

Functional Status Score for the ICU (FSS-ICU)

1. Characteristics

The FSS-ICU is an assessment measure of basic functional capabilities specifically designed for ICU patients.

2. Procedure and Method

The FSS-ICU evaluates physical function based on five criteria: (1) rolling over in bed, (2) sitting up, (3) maintaining a sitting position on the edge of the bed, (4) standing up, and (5) walking. Each item is scored out of 7, with a total score of 35. The FSS-ICU is useful for predicting whether patients may be directly discharged to their homes from acute care hospitals. The cut-off value for this prediction has been identified as 16 points [1]. A score of 22 or more on the FSS-ICU at the time of discharge predicted home discharge with sensitivity of 84.2% and specificity of 79.7% [2].

3. Other Information

There is no fee for using the FSS-ICU. Its validity was confirmed by significantly higher FSS-ICU scores among patients without ICU-acquired weakness and with hospital discharge to home. The minimal important difference was 2.0 to 5.0 [3].

References

[1] Shun A, Shinya M, Hitoshi Y, Rie T, Hironobu K. Cutoff values of functional status score for the ICU(FSS-ICU) to predict patient discharge in critically ill ICU patients. J Jpn Soc Intensive Care Med 2021;28:99-104.

[2] Tymkew H, Norris T, Arroyo C, Schallom M. The use of physical therapy ICU assessments to predict discharge home. Crit Care Med. 2020 Sep;48(9):1312-1318.

[3] Huang M, Chan KS, Zanni JM, Parry SM, Neto SGB, Neto JAA, Silva VZMd, Kho ME, Needham DM. Functional Status Score for the Intensive Care Unit (FSS-ICU): an international clinimetric analysis of validity, responsiveness, and minimal important difference. Crit Care Med. 2016 Dec;44(12):e1155-e1164.

Functional Ambulation Categories (FAC)

1. Characteristics

FAC were developed at Massachusetts General Hospital as a clinical evaluation metric for walking ability based on the amount of assistance required. It was initially introduced by Holden et al. in 1984 [1].

2. Procedure and Method

A walking path of approximately 15 meters or a set of stairs is used, and walking ability is classified into six categories based on an observational analysis. The use of assistive devices is not taken into account. However, some studies using FAC may not allow the use of walkers or wheeled walking aids.

3. Other Information

There is no fee for using FAC. A previous study that targeted stroke patients with hemiplegia 1-2 months post-onset showed excellent intra-rater reliability (kappa coefficient 0.950) and inter-rater reliability (kappa coefficient 0.905) [2]. This study also introduced key questions for appropriately assessing FAC. Good concurrent validity was demonstrated between FAC and the Rivermead Mobility Index (Spearman’s rho = 0.686–0.825), the 6-minute walk distance (rho = 0.931–0.949), and walking speed (rho = 0.902–0.952).

References

[1] Holden MK, Gill KM, Magliozzi MR, Nathan J. Clinical gait assessment in the neurologically impaired. Reliability and meaningfulness. Phys Ther. 1984;64(1):35-40.

[2] Mehrholz J, Wagner K, Rutte K, Meissner D, Pohl M: Predictive validity and responsiveness of the functional ambulation category in hemiparetic patients after stroke. Arch Phys Med Rehabil 2007;88(10):1314-9.

Cognitive Function Measurement Tests

- Montreal Cognitive Assessment (MoCA)
- Mini-Mental State Examination (MMSE)
- RBANS
- Trail Making Test
- TICS
- Short Memory Questionnaire (SMQ)
- Informant Questionnaire for Cognitive Decline in the Elderly(IQCODE)
- Cognitive Failures Questionnaire (CFQ)
- Cognitive and Affective Mindfulness Scale (CAMS)
- Wechsler Adult Intelligence Scale - Fourth Edition (WAIS-IV)
- Healthy Aging Brain Care (HABC) Monitor
- Rey Auditory-Verbal Learning Test (RAVLT)
- Cambridge Neuropsychological Test Automated Battery (CANTAB)
- Clinical Dementia Rating (CDR)

Montreal Cognitive Assessment (MoCA)

1. Characteristics

The MoCA was proposed by Nasreddine et al. in 2005 as an index for evaluating multi-domain cognitive functions [1]. It has been translated into many languages and is widely used worldwide, including the Japanese version, MoCA-J [1, 2]. The scoring methods for MoCA and MoCA-J are nearly identical, and both evaluate cognitive impairments in approximately 10 minutes.

2. Procedure and Method

Question items include visuospatial/executive functions, naming, digit span, attention, calculation, recall, verbal fluency, abstraction, delayed recall, and orientation. The test uses a questionnaire, and an additional point is added for respondents with 12 or fewer educational years. The score ranges from a minimum of 0 to a maximum of 30, with scores of 26 or higher being regarded as normal. A score of 25 or lower is suggestive of mild cognitive impairment (MCI). A cut-off value of 25 is associated with excellent screening sensitivity for MCI, with sensitivity of 80-100% and specificity of 50-87% [1, 3]. The American Association of Critical Care Medicine also strongly recommends the use of MoCA, classifying scores of 18-25 as MCI, 10-17 as moderate cognitive impairment, and scores lower than 10 as severe cognitive impairment [2]. T-MoCA stands for Telephone-MoCA and omits the visuospatial/executive and naming sections. It is performed over the phone and is scored out of 22 points. This T-MoCA score may be converted to MoCA using a conversion table, and the sensitivity and specificity of T-MoCA are 72 and 59%, respectively [4].

3. Other Information

There is no fee for using the MoCA. It was shown to have high test-retest reliability (ICC = 0.92), and a strong correlation was observed between MoCA and the Mini-Mental State Examination (MMSE) (r = 0.87) [5]. Internal consistency was Cronbach’s α value of 0.83 on standardized items.

References

[1] Nasreddine ZS, Phillips NA, Bédirian V, Charbonneau S. The Montreal Cognitive Assessment, MoCA: A brief screening tool for mild cognitive impairment. J Am Geriatr Soc. 2005;53(4):695-9.

[2] Mikkelsen ME, Still M, Anderson BJ, Bienvenu OJ. Society of Critical Care Medicine's international consensus conference on prediction and identification of long-term impairments after critical illness. Crit Care Med. 2020;48(11):1670-9.

[3] Fage BA, Chan CC, Gill SS, Noel-Storr AH. Mini-cog for the diagnosis of Alzheimer's disease dementia and other dementias within a community setting. Cochrane Database Syst Rev. 2015(2):Cd010860.

[4] Katz MJ, Wang C, Nester CO, Derby CA. T-MoCA: A valid phone screen for cognitive impairment in diverse community samples. Alzheimers Dement (Amst). 2021;13(1):e12144.

[5] Nasreddine ZS, Phillips NA, Bédirian V, Charbonneau S, Whitehead V, Collin I, Cummings JL, Chertkow H. The Montreal Cognitive Assessment, MoCA: a brief screening tool for mild cognitive impairment. J Am Geriatr Soc 2005;53(4):695-9.

MMSE

1. Characteristics

The MMSE was developed in 1975 by the Folsteins and is the most internationally used brief cognitive function test. It was originally used to screen psychiatric patients with cognitive impairment. Due to its simplicity, it is widely employed not only for psychiatric patients, but also for other patients.

2. Procedure and Method

Using 11 subtests — time & space orientation, immediate & delayed memory, arithmetic, naming, repetition, verbal command actions, reading, writing, and figure copying — the MMSE provides a comprehensive evaluation of cognitive function from a 30-point test. A score of 23 or lower suggests dementia (sensitivity 81% and specificity 89%) [1-2]. A score of 27 or lower is suggestive of MCI with sensitivity of 45-60% and specificity of 65-90% [3-5]. Questions begin from the first, and breaks may be taken during the test. There is a 10-second limit per question, and if exceeded, the examiner needs to move on to the next question. Adjustments to questions or the provision of hints is not allowed. Items required for the test include a scoring sheet, writing tools, a clock or key, and blank paper.

3. Other Information

Due to copyright issues, the official Japanese version, MMSE-J, needs to be purchased.

User’s guide: 1100 Japanese Yen

Recording sheet (for 50 individuals): 6600 Japanese Yen

Test and retest reliabilities have been demonstrated [6].

References

[1] Folstein MF, Folstein SE, McHugh PR. "Mini-mental state". A practical method for grading the cognitive state of patients for the clinician. J Psychiatr Res. 1975 Nov;12(3):189-98.

[2] Tsoi KK, Chan JY, Hirai HW, Wong SY. Cognitive tests to detect dementia: a systematic review and meta-analysis. JAMA Intern Med. 2015 Sep;175(9):1450-8.

[3] Tariq SH, Tumosa N, Chibnall JT, Perry MH 3rd. Comparison of the Saint Louis university mental status examination and the mini-mental state examination for detecting dementia and mild neurocognitive disorder--a pilot study. Am J Geriatr Psychiatry. 2006 Nov;14(11):900-10.

[4] Saxton J, Morrow L, Eschman A, Archer G. Computer assessment of mild cognitive impairment. Postgrad Med. 2009 Mar;121(2):177-85.

[5] Kaufer DI, Williams CS, Braaten AJ, Gill K. Cognitive screening for dementia and mild cognitive impairment in assisted living: comparison of 3 tests. J Am Med Dir Assoc. 2008 Oct;9(8):586-93.

[6] Thomas J. Lewis PhD, Clement L. Trempe MD. The End of Alzheimer's (Second Edition), 2017

Repeatable Battery for the Assessment of Neuropsychological Status (RBANS)

1. Characteristics:

The RBANS was developed by Randolph in 1998 to screen neuropsychological deficits in the elderly and younger patients. One of its defining features is the ability to assess various cognitive elements in a short period of time. The key features of RBANS are as follows:

The entire examination may be conducted in approximately 30 minutes. The difficulty level of the test is designed for healthy adults up to patients with moderate dementia. It allows for evaluations based on neuropsychological domains, and index scores may be used to create profiles. It has two equivalent forms (Form A and Form B), which ensures that learning effects are avoided, and it may be reused to evaluate treatment progress. Furthermore, the test equipment is compact and easily portable, allowing for bedside implementation.

2. Procedure and Methods:

The RBANS is designed to evaluate immediate memory, delayed memory, visuospatial/constructional capabilities, language, and attention. These five cognitive domains are assessed through 12 subtests, and the entire test may be administered in approximately 30 minutes.

3. Other Information:

There is no fee for using the RBANS. Minimal clinically important differences for the total score, language, immediate memory, delayed memory, visuospatial/constructional, and attention indexes were 8, 9, 10, 10, 6, and 4 points, respectively, in older adults [1].

Reference

[1] Phillips R, Qi G, Collinson SL, Ling A, Feng L, Cheung YB, Ng TP. The minimum clinically important difference in the Repeatable Battery for the Assessment of Neuropsychological Status. Clin Neuropsychol 2015; 29(7):905-23.

Trail Making Test (TMT)

1. Characteristics:

The TMT is a scale that holistically measures a wide range of cognitive functions, such as attention, working memory, spatial exploration, processing speed, perseverance, and impulsivity. In recent years, its positioning as one of the neuropsychological evaluation methods related to aptitude for automobile driving has been increasing. It may be widely used as an evaluation method for higher brain function disorders due to traumatic brain injuries, MCI, and relatively mild dementia as well as relatively pure executive function disorders, such as those represented by prefrontal cortex injuries [1].

2. Procedure and Methods:

Part A uses a dedicated sheet of paper on which numbers 1-25 are irregularly arranged. Participants are asked to connect the numbers from 1 to 25 as quickly and accurately as possible, and the time taken is measured. The score is represented by this time. TMT Part B involves drawing 24 circles on a sheet of paper, but not all circles contain numbers. Half of them contain numbers 1 through 12, and the other half contain letters A through L. In part B, the task is to alternate between numbers and letters in ascending order, namely, the “trail”.

➀Hand over the worksheet of TMT Part A with circles and numbers written on it.

➁Explain the instructions and demonstrate how Part A will be performed on a sample page.

➂Start timing immediately when the test begins.

➃If a mistake is made, inform the participant and ask them to correct it and continue.

➄Record the time when the participant finishes.

➅Repeat for TMT Part B.

➆If Part A and Part B are not completed within 5 minutes, the test may be terminated.

The cut-off value varies depending on the study.

3. Other information:

The standardized TMT-J is available for sale by the New Medical Publishing Company for 5,500 Yen. The interrater reliability on both parts is high (r PartA = 0.94; r PartB = 0.90) [2].

References

[1] Ashendorf L. Jefferson AL, O’Connor MK, Chaisson C. Trail making test errors in normal aging, mild cognitive impairment, and dementia. Arch Clin Neuropsychol. 2008 Mar;23(2):129–37.

[2] Fals-Stewart W. An Interrater Reliability Study of the Trail Making Test (Parts A and B). Perceptual and Motor Skills 1992;74(1):39-42.

Telephone Interview for Cognitive Status (TICS)

1. Characteristics:

TICS was developed in 1988 by Brandt and others based on the MMSE. It is a cognitive screening test intended to be conducted over the phone.

2. Procedure and Methods:

The test involves the following:

Orientation to name (full name of the examinee: 2 points).

Time orientation (year, month, day, day of the week, season: 5 points).

Place orientation (location of the examinee: postal code, prefecture, city, street, address: 5 points).

Counting backward from 20 to 1 (2 points).

Immediate recall of 10 words (10 points).

Subtraction series of 7 (5 points).

Language (answering 4 described words: 4 points).

Repetition of sentences (2 short sentences: 2 points).

Recent memory (name of the Prime Minister: 2 points).

Practical task (tapping the phone receiver: 2 points).

Antonyms (2 types: 2 points).

These components culminate in a total of 41 points.

3. Other information

The TICS Introductory Kit (English version) costs $150. Time orientation and the subtraction series of 7 are identical to those in the MMSE. The orientation to place and sentence repetition partially overlap with the MMSE. TICS correlates well with the MMSE, exhibits excellent reproducibility, and has sufficient sensitivity and specificity to detect cognitive impairment. Pearson’s correlation coefficient between TICS and R-CAMCOG (a modification of the cognitive part of the Cambridge Examination for Mental Disorders of the Elderly) was 0.833 in post-stroke subjects [1].

Reference

[1] Barber M, Stott DJ. Validity of the Telephone Interview for Cognitive Status (TICS) in post-stroke subjects. Int J Geriatr Psychiatry 2004; 19(1):75-9.

Short Memory Questionnaire (SMQ)

1. Characteristics:

The SMQ was developed in 1993 by Koss and others. It was designed to indirectly evaluate a patient’s cognitive impairment through interviews with caregivers. The SMQ is anticipated to be useful for early consultations and diagnoses. A strong correlation has been observed between SMQ and MMSE [1].

2. Procedure and Methods:

The questionnaire consists of 14 items related to recent events and executive functions. Responses range from four categories, of “cannot do” to “can always do”. Two questions (“Do you remember the birthdays of family members?” and “Can you promptly say the word you intend to say?”) are subtracted from the total score. The total score ranges from 4 to 46 points. A score lower than 40 suggests a potential cognitive impairment. In a domestic epidemiological study using the SMQ, in which a decline from the time of admission or scores lower than 40 were defined as cognitive impairment, the frequency of cognitive dysfunction was 37.5% six months after discharge [2].

3. Others:

There is no fee for using the SMQ. Internal consistency was Cronbach’s α value of 0.85 in Alzheimer’s disease [3].

References

[1] 牧 徳彦．日本語版Short-Memory Questionnaire—アルツハイマー病患者の記憶障害評価法の有用性の検討. 脳と神経 50：415-418, 1998.

[2] Kawakami D, Fujitani S, Morimoto T, Dote H. Prevalence of post-intensive care syndrome among Japanese intensive care unit patients: a prospective, multicenter, observational J-PICS study. Crit Care. 2021 Feb 16;25(1):69.

[3] Koss E, Patterson MB, Ownby R, Stuckey JC, Whitehouse PJ. Memory evaluation in Alzheimer's disease. Caregivers' appraisals and objective testing. Arch Neurol 1993;50(1):92-7.

Informant Questionnaire for Cognitive Decline in the Elderly (IQCODE)

1. Characteristics:

The IQCODE was developed in 1989 by Jorm as a cognitive function screening test. It is useful under conditions where a subject cannot be directly assessed or when a test is needed that is not affected by the educational level of an individual [1].

2. Procedure and Methods:

The IQCODE assesses a wide range of cognitive and functional areas, including recent memory, remote memory, time and place orientation, financial awareness, learning, and executive function. It may be used to differentiate between MCI and dementia. A review of previous studies confirmed this ability with sensitivity of 69 to 100% and specificity of 65 and 96%.

3. Other information:

There is no fee for using the IQCODE. It was shown to have high internal reliability in a general population sample (alpha = 0.95) and reasonably high test-retest reliability over one year in a demented sample (r = 0.75), and the total IQCODE score discriminated well between a general population and demented population [2].

References

[1] Anthony F Jorm. The informant questionnaire on cognitive decline in the elderly (IQCODE). Int Psychogeriatr. 2004 Sep;16(3):275-93.

[2] Jorm AF, Jacomb PA: The Informant Questionnaire on Cognitive Decline in the Elderly (IQCODE): socio-demographic correlates, reliability, validity and some norms. Psychol Med 1989; 19(4):1015-22.

Cognitive Failures Questionnaire (CFQ)

1. Characteristics:

The self-rated CFQ was devised in 1982. It is a 25-item self-report questionnaire that assesses impairments in perception, memory, and motor function in the completion of everyday tasks over the past six months.

2. Procedure and Methods:

The scoring for the CFQ ranges between 0 and 100. A score of 100 is given if all items are answered as “very often”, while a score of 0 is given if all items are answered as “not at all”.

3. Other information:

There is no fee for using the CFQ. In the Hungarian translation, test-retest reliability was an intraclass correlation coefficient of 0.900, and internal consistency was Cronbach’s α value of 0.920 [1].

Reference

[1] Volosin M, Hallgató E, Csábi E. Validation of the Hungarian version of the Cognitive Failures Questionnaire (CFQ). Heliyon 2023; 9(1):e12910.

Cognitive and Affective Mindfulness Scale (CAMS)

1. Characteristics:

The CAMS is a self-reported measure that comprises several items designed to capture the multi-dimensional nature of mindfulness. In contrast to other scales, the CAMS is not tied to a particular therapeutic intervention, allowing its use for a broader range of populations. This scale attempts to offer a concise measure of mindfulness suitable for those with and without experience of meditation.

2. Procedure and Methods:

Participants evaluate 12 items based on how often they apply to them. These items are rated on a 4-point Likert scale, ranging from 1 (rarely/not at all) to 4 (almost always). The revised version of CAMS was found to have good internal reliability (Cronbach’s α value of 0.75 to 0.87 and a corrected item-total correlation of 0.44 to 0.81) [1].

3. Other Information

There is no fee for using the CAMS.

Reference

[1] Huang F, Chen WT, Shiu CS, Lin SH, Tun MS, Nwe TW, Nu Oo YT, Oo HN. Adaptation and validation of the Cognitive and Affective Mindfulness Scale-Revised (CAMS-R) in people living with HIV in Myanmar. Mindfulness (N Y) 2022;13(1):188-97.

Wechsler Adult Intelligence Scale - Fourth Edition (WAIS-IV)

1. Characteristics

The WAIS-IV, with its origins in the Wechsler-Bellevue Intelligence Test released in 1993, is an intelligence test with a history spanning more than 70 years. It is used in many countries worldwide and is one of the most commonly used tests for assessing intelligence during childhood and adulthood.

2. Procedure and Method

The WAIS-IV is a comprehensive clinical test administered individually, designed to measure intelligence in individuals ranging between 16 and 90 years and 11 months. The test produces four composite scores (VCI, PRI, WMI, and PSI) that represent specific cognitive domains as well as a composite score (FSIQ) that represents overall intelligence.

3. Other Information:

The Japanese version of the WAIS-IV is available from Nihon Bunka Kagakusha for 154,000 yen. Test-retest coefficients ranged between 0.74 (Visual Puzzles subtest) and 0.90 (Information subtest), and WAIS had excellent inter-rater reliability of 0.98 to 0.99 [1].

Reference

[1] Grimes KM, Zanjani A. Zakzanis KK. Memory impairment and the mediating role of task difficulty in patients with schizophrenia. Psychiatry Clin Neurosci, 2017;71(9):600-11.

Healthy Aging Brain Care (HABC) Monitor

1. Characteristics

The HABC Monitor was developed in 2008 with two parallel versions. The caregiver-report version evaluates based on observations and perceptions of a patient’s caregivers, while the self-report version gathers information directly from the patients themselves.

2. Procedure and Method

Both versions of the tool consist of 27 items that measure three domains of patient symptoms: cognitive, functional, and psychological. The caregiver-report version of the HABC Monitor is a reliable, valid, and clinically practical multidimensional tool designed to measure and monitor the severity of a patient’s symptoms through caregiver reports.

3. Other Information:

There is no Japanese version. The HABC Monitor showed excellent internal consistency (Cronbach’s α value of 0.88-0.93). Scores on the cognitive subscale correlated with the MMSE (Spearman’s ρ = -0.33) [1].

Reference

[1] Wang S, Jawed Y, Perkins A, Gao S, Seyffert S, Khan S, Boustani M, Khan B: Healthy Aging Brain Care Monitor, caregiver version: screening for Post-Intensive Care Syndrome. Am J Crit Care 2022; 31(2):137-44.

Rey Auditory-Verbal Learning Test (RAVLT)

1. Characteristics

The RAVLT is an auditory memory test designed to assess immediate memory and short-term memory capacity as well as retention and retrieval capabilities.

2. Procedure and Method

Participants are read a list of 15 words (List A) and are then asked to recall as many words as possible in any order (immediate recall). A different list of 15 words (List B) is then read to participants and they are asked to recall them in the same manner. Participants are subsequently asked to recall words from List A that they still remember (delayed recall).

3. Other Information:

There is no fee to use the test. All test-retest correlation coefficients achieved significance, ranging between 0.36 and 0.68, and Cronbach’s α value was 0.80. RAVL, the sum of A1-A5 and A7, did not correlate with TMT, but modestly correlated with BVRT (ranging between 0.37 and 0.44) [1].

Reference

Magalhães SS, Malloy-Diniz L, Hamdan A. Validity convergent and reliability test-retest of the Rey auditory verbal learning test. Clinical Neuropsychiatry 2012; 9:129-37.

Cambridge Neuropsychological Test Automated Battery (CANTAB)

1. Characteristics

CANTAB is a globally utilized neuropsychological assessment that evaluates not only memory, but also various cognitive functions, such as working memory, motor functions, attention, spatial cognition, understanding, and executive functions. An application for smartphones and tablets is available, which eliminates the need for direct testing (it is only available in English).

2. Procedure and Method

The test is a simple point-of-care assessment lasting approximately 10 minutes. The platform includes three tests focusing on memory, mood, and the ability to perform routine daily activities. Age, sex, and educational level are also considered in the test. The findings obtained are presented in a one-page doctor’s report, enabling users to alleviate concerns and possibly refer patients to specialists.

3. Other Information:

There is a fee to use the application. The Spatial Working Memory test, Attention Switching Task, and Rapid Visual Processing test are the only tests with scores of adequate test-retest reliabilities [1]. Regarding all outcome measures, Pearson’s and Spearman’s correlation coefficients ranged between 0.39 and 0.79, and the measurement error surrounding difference scores was large.

Reference

[1] Karlsen RH, Karr JE, Saksvik SB, Lundervold AJ, Hjemdal O, Olsen A, Iverson GL, Skandsen T. Examining 3-month test-retest reliability and reliable change using the Cambridge Neuropsychological Test Automated Battery. Appl Neuropsychol Adult 2022;29(2):146-54.

Clinical Dementia Rating (CDR)

1. Characteristics

The CDR is a scale that assesses the severity of dementia. It was initially introduced by Hughes and colleagues in 1982 and has been widely adopted internationally. In contrast to other assessments that score cognitive functions based on tests, the CDR evaluates patients based on their everyday activities, such as hobbies, social activities, and housekeeping. It is not used for diagnostic purposes.

2. Procedure and Method

There are five stages, ranging from healthy to severe dementia. The CDR assesses six domains: memory, orientation, judgment and problem-solving, community affairs, home and hobbies, and personal care. These evaluations are based on interviews with caregivers. In contrast to other scales, the CDR does not give a composite score; it provides independent judgments on each symptom of dementia.

3. Other Information:

There is no fee for using the CDR. CDR scores showed good internal consistency (Cronbach’s α value of 0.83-0.84), inter-rater and test-retest reliabilities, and good agreement (κ 0.79) with the clinical assessment status of MCI and dementia [1].

Reference

[1] Nyunt MS, Chong MS, Lim WS, Lee TS, Yap P, Ng TP. Reliability and validity of the clinical dementia rating for community-living elderly subjects without an informant. Dement Geriatr Cogn Dis Extra 2013;3(1):407-16.

Mental Function Measurement Tests

- Hospital Anxiety and Depression Scale (HADS)
- Impact of Event Scale-Revised (IES-R)
- Patient Health Questionnaire-9 (PHQ-9)
- Post-Traumatic Stress Scale 10 (PTSS-10)
- Post-Traumatic Stress Disorder Checklist for DSM-5 (PCL-5)
- Beck Depression Inventory-II (BDI-II)
- Generalized Anxiety Disorder-7 (GAD-7)
- PCL- Civilian version (PCL-C)
- IES-15 items (IES-15)
- PTSS-14
- PCL-Specific (PCL-S)
- IES-6
- PHQ-2
- Numerical Rating Scale (NRS)
- DASS (Depression Anxiety Stress Scale)-21
- State Trait Anxiety Inventory (STAI)
- Centre for Epidemiological Studies-Depression Scale (CES-D)
- Visual Analogue Scale-Anxiety (VAS-A)
- Brief Coping Orientation to Problems Experienced (COPE) Inventory
- Trauma Screening Questionnaire (TSQ)
- Major Depression Inventory (MDI)
- PHQ-8
- PHQ-4
- Davidson Trauma Scale (DTS)

HADS (Hospital Anxiety Depression Scale)

1. Characteristics

The HADS is an evaluation scale for symptoms of anxiety and depression developed by Zigmond and others in 1983 [1]. It consists of 14 questions, divided into two sub-scores: an anxiety sub-score (7 items) and depression sub-score (7 items). It was created to assess mental symptoms associated with physical diseases, thereby excluding as many items affected by physical conditions as possible. Each item is rated on a 4-point scale of 0 to 3, and the total for each of the anxiety and depression sub-scores ranges between 0 and 21. Higher scores indicate more severe symptoms of anxiety or depression.

2. Procedure and Method

The scale is administered via a questionnaire, which typically takes approximately 5 minutes to complete. Various cut-offs are used for the anxiety and depression sub-scores. Previous studies, particularly in PICS research, define scores of 8 or higher as indicative of significant symptoms of depression or anxiety [2]. However, a few PICS studies define scores of 7 or higher as indicative of significant symptoms [3]. Meta-analyses suggested that for the depression sub-score, the appropriate cut-off for screening was 7 or 8, with sensitivity of 0.82 and specificity of 0.78 for the 7-point cut-off, sensitivity of 0.74 and specificity of 0.84 for the 8-point cut-off, and sensitivity of 0.44 and specificity of 0.95 for the 11-point cut-off [4]. The 8-point cut-off is commonly used for the anxiety sub-score, with overall sensitivity of 0.699 and specificity of 0.787 [5]. The total score combining both sub-scores may be used to detect mental symptoms, with sensitivity of 0.728 and specificity of 0.657 [6].

3. Other Information:

There is no fee for using the HADS. Its validity and reliability have been examined in numerous studies on various populations [7].

References

[1] Zigmond AS, Snaith RP. The hospital anxiety and depression scale. Acta Psychiatr. Scand. 1983; 67: 361–70.

[2] Geense WW, Zegers M, Peters MAA, Ewalds E. New physical, mental, and cognitive problems 1 year after icu admission: a prospective multicenter study. Am J Respir Crit Care Med. 2021 Jun 15;203(12):1512-1521.

[3] Azoulay E, Resche-Rigon M, Megarbane B, Reuter D. Association of COVID-19 Acute respiratory distress syndrome with symptoms of posttraumatic stress disorder in family members after ICU discharge. JAMA. 2022 Mar 15;327(11):1042-1050.

[4] Wu Y, Levis B, Sun Y, He C. Accuracy of the hospital anxiety and depression scale depression subscale (HADS-D) to screen for major depression: systematic review and individual participant data meta-analysis. BMJ. 2021 May 10;373:n972.

[5] Mitchell AJ, Meader N, Symonds P. Diagnostic validity of the hospital anxiety and depression Scale (HADS) in cancer and palliative settings: a meta-analysis. J Affect Disord. 2010 Nov;126(3):335-48.

[6] Mikkelsen ME, Still M, Anderson BJ, Bienvenu OJ. Society of Critical Care Medicine's international consensus conference on prediction and identification of long-term impairments after critical illness. Crit Care Med. 2020

[7] Bjelland I, Dahl AA, Haug TT, Neckelmann D. The validity of the Hospital Anxiety and Depression Scale. An updated literature review. J Psychosom Res 2002;52(2):69-77.

Impact of Event Scale-Revised (IES-R)

1. Characteristics

The IES-R is an assessment scale created by Weiss and colleagues in 1977. It consists of 22 questions, with each item being scored from 0 to 4 points [1]. The reliability and validity of the Japanese version of the IES-R was verified in the Japanese population, and a total score of 25 points or more is considered to be the cut-off for screening post-traumatic stress disorder (PTSD) symptoms among Japanese individuals [2]. It follows the criteria of DSM-IV and has not been revised to match DSM-5.

2. Procedure and Method

The scale is administered using a questionnaire method and typically takes approximately 5 minutes to complete. It assesses intrusive, avoidance, and hyperarousal symptoms. The scoring method involves summing the total points or taking an average score. In a previous study that targeted patients with acute lung injury, when a cut-off of an average score of 1.6 (corresponding to a total score of 35.2 points) was used, sensitivity ranged between 80 and 100% and specificity between 85 and 91% [3]. The 2019 consensus conference of the American Society of Critical Care Medicine weakly recommends using the IES-R with a cut-off average score of 1.6 points for assessing PTSD symptoms [4].

3. Other Information:

There is no fee associated with the use of the IES-R. It has repeatedly been used in the context of PICS [5]. Test-retest reliability was r of 0.86 [6]. IES-R is an important measure for evaluating PTSD in patients with acute respiratory failure, and a psychiatrist’s PTSD diagnosis is considered the gold standard, underscoring its validity in this context [7].

References

[1] Weiss DS, Marmar CR. The impact of event scale – revised. In: Wilson JP, Keane TM (eds). Assessing Psychological Trauma and PTSD. vol. 19. New York: Guilford Press, 1997;399–411.

[2] Asukai N, Kato H, Kawamura N, Kim Y. Reliability and validity of the Japanese-language version of the impact of event scale-revised (IES-R-J): four studies of different traumatic events. J Nerv Ment Dis. 2002 Mar;190(3):175-82.

[3] Bienvenu OJ, Williams JB, Yang A, Hopkins RO. Posttraumatic stress disorder in survivors of acute lung injury: evaluating the Impact of Event Scale-Revised. Chest. 2013 Jul;144(1):24-31.

[4] Mikkelsen ME, Still M, Anderson BJ, Bienvenu OJ. Society of Critical Care Medicine's international consensus conference on prediction and identification of long-term impairments after critical illness. Crit Care Med. 2020 Nov;48(11):1670-1679.

[5] Hosey MM, Bienvenu OJ, Dinglas VD, Turnbull AE. The IES-R remains a core outcome measure for PTSD in critical illness survivorship research. Crit Care. 2019 Nov 19;23(1):362.

[6] Asukai N, Kato H, Kawamura N, Kim Y, Yamamoto K, Kishimoto J, Miyake Y, Nishizono-Maher A. Reliability and validity of the Japanese-language version of the impact of event scale-revised (IES-R-J): four studies of different traumatic events. J Nerv Ment Dis 2002; 190(3):175-82.

[7] Bienvenu OJ, Williams JB, Yang A, Hopkins RO. Posttraumatic stress disorder in survivors of acute lung injury: evaluating the Impact of Event Scale-Revised. Chest 2013;144:24–31.

Patient Health Questionnaire-9 (PHQ-9)

1. Characteristics

The PHQ was developed in the United States as a screening tool for mental illnesses for primary care physicians [1]. PHQ-9 is a self-administered questionnaire extracted from the PHQ, focusing specifically on 9 questions related to major depressive disorder. A distinctive feature of PHQ-9 is its condensation of various symptoms into a set of 9 questions [2].

2. Procedure and Method

The scale is administered using a questionnaire and typically takes less than 5 minutes to complete. It consists of 9 items, each scored from 0 to 3, resulting in a total possible score range of 0 to 27. Higher scores indicate more severe depressive symptoms. Apart from being a general screening tool for depression, it may also be used to assess depression related to physical illnesses. The American Heart Association also recommends its use [3]. A score of 10 or more has been reported to have sensitivity of 88% and specificity of 88% in diagnosing major depressive disorder, suggesting that an evaluation by a specialist is desirable [2]. Its validity has been confirmed, and a cut-off score of 10 or higher has sensitivity of 90.5% and specificity of 76.6% [4].

3. Other Information:

There are no fees associated with using the PHQ. Some studies related to PICS also utilized PHQ-9 [5]. Its internal reliability was shown to be 0.89, while its test-retest reliability within 48 hours was 0.84. Its correlations with Medical Outcomes Study Short-Form General Health Survey (SF-20) were 0.73 in mental health, 0.55 in general health perceptions, 0.52 in social functioning, 0.43 in role functioning, 0.37 in physical functioning, 0.33 in bodily pain [6].

References

[1] Spitzer RL, Kroenke K, Williams JB. Validation and utility of a self-report version of PRIME-MD: the PHQ primary care study. Primary care evaluation of mental disorders. patient health questionnaire. JAMA. 1999 Nov 10;282(18):1737-44.

[2] Kroenke K, Spitzer RL, Williams JB. The PHQ-9: validity of a brief depression severity measure. J Gen Intern Med. 2001 Sep;16(9):606-13.

[3] Carney RM, Freedland KE. Depression and coronary heart disease. Nat Rev Cardiol. 2017 Mar;14(3):145-155.

[4] Muramatsu K, Miyaoka H, Kamijima K, Muramatsu Y. Performance of the Japanese version of the patient health questionnaire-9 (J-PHQ-9) for depression in primary care. Gen Hosp Psychiatry. 2018 May-Jun;52:64-69.

[5] Evans RA, McAuley H, Harrison EM, Shikotra A. Physical, cognitive, and mental health impacts of COVID-19 after hospitalisation (PHOSP-COVID): a UK multicentre, prospective cohort study. Lancet Respir Med. 2021 Nov;9(11):1275-1287.

[6] Kroenke K, Spitzer RL, Williams JB. The PHQ-9: validity of a brief depression severity measure. J Gen Intern Med 2001;16(9):606-13.

Post-Traumatic Stress Scale 10 (PTSS-10)

1. Characteristics

PTSS-10 is a questionnaire-based assessment scale for PTSD symptoms developed based on DSM-III. Originally designed for PTSD screening in victims of natural disasters and torture [1], its applicability to evaluating post-ICU symptoms was reported by Stoll and others in 1999 [2].

2. Procedure and Method

The scale is administered via a questionnaire method, and it typically takes less than 5 minutes to complete. Comprising 10 items, each item is rated on a 7-point scale of 1 (not at all) to 7 (always present). The total score ranges between 10 and 70. A score of 35 or more suggests the presence of PTSD. A previous study reported sensitivity of 77.0% and specificity of 97.5% for the diagnosis of PTSD using this scale [1].

3. Other Information:

There is no fee for using the PTSS-10. Higher values on PTSS-10 were associated with decreased QOL in chronic critically ill patients [3]. Internal consistency was Cronbach’s α value of 0.83, while validity was ρ of 0.77 [4].

References

[1] Weisaeth L. Torture of a Norwegian ship's crew. The torture, stress reactions and psychiatric after-effects. Acta Psychiatr Scand Suppl. 1989;355:63-72.

[2] Stoll C, Kapfhammer HP, Rothenhäusler HB, Haller M. Sensitivity and specificity of a screening test to document traumatic experiences and to diagnose post-traumatic stress disorder in ARDS patients after intensive care treatment. Intensive Care Med 1999;25:697-704.

[3] Wintermann G-B, Petrowski K, Weidner K, Strauß B, Rosendahl J. Impact of post-traumatic stress symptoms on the health-related quality of life in a cohort study with chronically critically ill patients and their partners: age matters. Crit Care 2019;23(1):39.

[4] Rosendahl J, Kisyova H, Gawlytta R, Scherag A. Comparative validation of three screening instruments for posttraumatic stress disorder after intensive care. J Crit Care 2019;53:149-54.

Post-Traumatic Stress Disorder Checklist for DSM-5 (PCL-5)

1. Characteristics

The PCL-5 is a self-administered assessment scale for PTSD symptoms that was introduced in 2015 [1]. It aligns with the diagnostic criteria for DSM-5. It distinctly assesses all the categories present in DSM-5, which include re-experiencing symptoms (5 items), avoidance symptoms (2 items), negative changes in cognition and mood (7 items), and marked changes in arousal and reactivity (6 items). There are versions that assess symptoms in the past month as well as in the past week.

2. Procedure and Method

The scale is administered via a questionnaire, and it typically takes approximately 10 minutes to complete. Comprising 20 items, participants rate how much they have been bothered by the symptoms in the past week, scoring each on a scale of 0 to 4, leading to a total score of 0-80. Higher scores indicate stronger PTSD symptoms. Regarding cut-off scores, using 37 points or more yielded sensitivity of 66% and specificity of 97%. Using 31 points or more resulted in sensitivity of 77% and specificity of 96%. Twenty-eight points or higher had sensitivity of 78% and specificity of 95% [1]. In the evaluation of PTSD symptoms in hospitalized Covid-19 patients, a range of cut-offs, such as 31 points [3] and 38 points or higher [2], was used.

3. Other Information:

There is no fee for using the scale. The Japanese version of PCL-5 strongly correlated with other PTSD symptom assessment scales, such as IES-R [3]. To access the Japanese version of PCL-5, the authors of 5 specific references may be contacted or publications that have cited it may be referred to [4,5]. Internal consistency was Cronbach’s α value of  0.92 and validity was ρ of 0.90 [6].

References

[1] Blevins CA, Weathers FW, Davis MT, Witte TK. The posttraumatic stress disorder checklist for DSM-5 (PCL-5): development and initial psychometric evaluation. J Trauma Stress. 2015;28(6):489-498.

[2] Writing Committee for the COMEBAC Study Group; Morin L, Savale L, Pham T, Colle R. Four-month clinical status of a cohort of patients after hospitalization for COVID-19. JAMA. 2021 Apr 20;325(15):1525-1534.

[3] Evans RA, McAuley H, Harrison EM, Shikotra A. Physical, cognitive, and mental health impacts of COVID-19 after hospitalisation (PHOSP-COVID): a UK multicentre, prospective cohort study. Lancet Respir Med. 2021 Nov;9(11):1275-1287.

[4] Ito M, Takebayashi Y, Suzuki Y, Horikoshi M. Posttraumatic stress disorder checklist for DSM-5: Psychometric properties in a Japanese population. J Affect Disord. 2019 Mar 15;247:11-19.

[5] パトリシア A. リーシック・キャンディス A. マンソン・キャスリーン M. チャード（著）伊藤正哉・堀越勝（監修）（2019）認知処理療法　治療者のための包括手引き, 創元社.

[6] Rosendahl J, Kisyova H, Gawlytta R, Scherag A. Comparative validation of three screening instruments for posttraumatic stress disorder after intensive care. J Crit Care 2019; 53:149-54.

Beck Depression Inventory-II (BDI-II)

1. Characteristics

BDI-II is an assessment scale developed by Beck and colleagues to measure the severity of depressive symptoms. It evaluates not only cognitive distortions, such as despair, irritability, feelings of guilt, and feeling punished, but also physical symptoms, such as fatigue, weight loss, and decreased libido. BDI-II is the 1996 revised version of BDI, which was introduced in 1961 to align with the DSM-4 criteria [1, 2].

2. Procedure and Method

The scale is administered via a questionnaire. Its completion typically takes approximately 5-10 minutes. It consists of 21 items, and each item is scored on a scale of 0 to 3, resulting in a total score of 0 to 63. A score of 14 or higher suggests mild depression, 20 or higher indicates moderate depression, and 29 or higher suggests severe depression. However, the user manual recommends adjusting the cut-off score based on the target patient group and specific purpose. BDI-II has also been adopted in the BRAIN-ICU scale [3].

3. Other information:

A fee is required to use BDI-II. The user manual costs ¥4,400, and questionnaire sheets for 50 people cost ¥13,200. BDI has also shown high construct validity with medical symptoms with a coefficient alpha rating of 0.92 for outpatients and 0.93 for student samples. BDI-II correlated with the Hamilton Depression Rating Scale (r = 0.71) [4].

References

[1] Beck AT, Steer RA, Ball R, Ranieri W. Comparison of Beck depression inventories -IA and -II in psychiatric outpatients. J Pers Assess. 1996 Dec;67(3):588-97.

[2] Beck AT, Steer RA, Brown G. BDI-II depression inventory manual. New York, NY: Harcourt Brace, 1996.

[3] Jackson JC, Pandharipande PP, Girard TD, Brummel NE. Depression, post-traumatic stress disorder, and functional disability in survivors of critical illness in the BRAIN-ICU study: a longitudinal cohort study. Lancet Respir Med. 2014;2:369–79.

[4] Christodoulaki A, Baralou V, Konstantakopoulos G, Touloumi G. Validation of the Patient Health Questionnaire-4 (PHQ-4) to screen for depression and anxiety in the Greek general population. J Psychosom Res. 2022;160:110970.

Generalized Anxiety Disorder-7 (GAD-7)

1. Characteristics

GAD-7 is an assessment scale that was developed in 2006 to screen anxiety disorders. It is specifically targeted towards GAD, allowing both for its screening and an evaluation of the severity of its symptoms [1]. GAD-7 was developed to include all symptom criteria of DSM-IV. This scale was derived from the PHQ to assess anxiety symptoms.

2. Procedure and Method

The scale is administered via a questionnaire. Completion typically takes less than 5 minutes. It consists of 7 items, and each item is scored on a scale of 0 to 3, resulting in a total score of 0 to 28. A higher score indicates more severe symptoms of GAD. Cut-off scores of 8 or 10 are used to suggest the presence of an anxiety disorder. A score of 8 or higher (sensitivity 92%, specificity 76%) is utilized for screening purposes to maximize sensitivity. However, a score of 10 or higher was reported to offer balanced sensitivity (89%) and specificity (82%) [2]. The effectiveness of GAD-7 as a screening tool for anxiety disorders in primary care settings has also been demonstrated [2].

3. Other Information:

No fee is required to use GAD-7. The reliability coefficient, Cronbach’s α value, for the overall GAD-7 scale was 0.895 [3].

References

[1] Spitzer RL, Kroenke K, Williams JB, Löwe B. A brief measure for assessing generalized anxiety disorder: the GAD-7. Arch Intern Med. 2006 May 22;166(10):1092-7.

[2] Kroenke K, Spitzer RL, Williams JB, Monahan PO. Anxiety disorders in primary care: prevalence, impairment, comorbidity, and detection. Ann Intern Med. 2007 Mar 6;146(5):317-25.

[3] Dhira TA, Rahman MA, Sarker AR, Mehareen J. Validity and reliability of the Generalized Anxiety Disorder-7 (GAD-7) among university students of Bangladesh. PLoS One 2021;16(12):e0261590.

PTSD Checklist, Civilian version (PCL-C)

1. Characteristics

The PCL-C is a 17-item scale developed as a shortened version of PCL-5, designed to evaluate various mental traumas experienced by civilians [1]. It consists of 5 items for re-experiencing symptoms, 7 for emotional numbing or avoidance symptoms, and 5 for hyperarousal symptoms.

2. Procedure and Method

The scale is administered using a questionnaire. Completion generally takes approximately 5-10 minutes. It consists of 17 items, and each item is scored on a scale of 1 to 5, resulting in a total score of 17 to 85. A score of 28 or 30 and higher suggests the presence of PTSD symptoms [1]. To enhance specificity, some studies used a higher cut-off of 45 (sensitivity 60%, specificity 99%) [2]. The PICS study reported that PTSD was considered to be present when the score was 45 or higher. In this study, the prevalence of PTSD three months after discharge from the ICU was 22% [3].

3. Other Information:

No fee is required to use the PCL-C. It showed good internal consistency and retest reliability as well as favorable patterns of convergent and discriminant validity [4].

References

[1] Weathers, F.W.; Litz, B.T.; Herman, D.S.; Huska, J.A.; Keane, T.M. The PTSD Checklist (PCL): reliability, validity, and diagnostic utility. In Proceedings of the Annual Convention of the International Society for Traumatic Stress Studies, San Antonio, TX, USA, 1993.

[2] Andrykowski MA, Cordova MJ, Studts JL, Miller TW. Posttraumatic stress disorder after treatment for breast cancer: prevalence of diagnosis and use of the PTSD Checklist - Civilian Version (PCL-C) as a screening instrument. J Consult Clin Psychol. 1998;66:586–90.

[3] Hatch R, Young D, Barber V, Griffiths J. Anxiety, depression and post traumatic stress disorder after critical illness: a UK-wide prospective cohort study. Crit Care. 2018 Nov 23;22(1):310.

[4] Conybeare D, Behar E, Solomon A, Newman MG, Borkovec TD. The PTSD Checklist—Civilian Version: Reliability, Validity, and Factor Structure in a Nonclinical Sample. J Clin Psychol 2012;68(6):699-713.

IES- 15 items (IES-15)

1. Characteristics

Introduced in 1979, the IES is a scale for evaluating PTSD symptoms and served as a precursor to the IES-R [1]. It consists of 15 items to assess intrusion and avoidance symptoms. The IES-R was subsequently developed by adding 7 items for hyperarousal symptoms. Similar to the IES-R, the IES aligns with the DSM-IV criteria and does not include elements such as cognitive and mood changes, which are covered in DSM-5. IES is one of the earlier scales created to assess PTSD.

2. Procedure and Method

The IES is a questionnaire that typically takes approximately 5 minutes to complete. Scoring methods involve summing up the total score (from 0 to 60) or calculating the average score (from 0 to 4). While some studies have used it as a criterion for PTSD symptoms following trauma [2], other scales are more commonly used.

3. Other Information:

There are no fees associated with using the IES. It was shown to be reliable and valid to assess PTSD in postburn reactions [3].

References

[1] Horowitz M, Wilner N, Alvarez W. Impact of event scale: a measure of subjective stress. Psychosom Med. 1979 May;41(3):209-18.

[2] Rzeszutek M, Lis-Turlejska M, Pięta M, Szumiał S. The polish adaptation of the disclosure of trauma questionnaire (DTQ). Psychiatr Pol. 2021 Dec 31;55(6):1293-1304. English, Polish.

[3] Echevarria-Guanilo ME, Dantas RA, Farina JA, Jr., Alonso J, Rajmil L, Rossi LA. Reliability and validity of the Impact of Event Scale (IES): version for Brazilian burn victims. J Clin Nurs 2011;20(11-12):1588-97.

PTSS-14

1. Characteristics

PTSS-14 is a questionnaire-based scale for evaluating PTSD symptoms [1]. It was modified from PTSS-10, which conformed to DSM-III, by adding several items, making it compliant with DSM-IV.

2. Procedure and Method

PTSS-14 is conducted using a questionnaire format. It typically takes approximately 5 minutes to complete. The scale has 14 items, and respondents rate each item on a scale of 1 (not at all) to 7 (always). The total score ranges from 14 to 98. A score of 45 or higher on PTSS-14 two months after leaving the ICU suggests the presence of PTSD. A previous study reported that the sensitivity of this diagnosis was 86% and its specificity was 97% [1].

3. Other Information:

There are no fees associated with the use of PTSS-14. In comparisons with the IES-R, the sensitivity of PTSS-14 was lower, whereas its specificity was higher. PTSS-14 is also considered to be more convenient because it has fewer items than the IES-R. Therefore, in an RCT on acute respiratory distress syndrome (ARDS), PTSS-14 was considered to be more sensitive at detecting differences in the prevalence of PTSD symptoms than the IES-R and was adopted instead of IES-R [2]. However, in contrast to the IES-R, PTSS-14 contains only 2 questions related to the primary symptom of PTSD, which is the re-experiencing symptom (intrusion symptom), and includes questions not related to the main symptoms (such as emotional fluctuations and muscle tension) [3]. Internal consistency was Cronbach’s α value of  0.88 and validity was ρ of 0.82 [4].

References

[1] Twigg E, Humphris G, Jones C, Bramwell R. Use of a screening questionnaire for post-traumatic stress disorder (PTSD) on a sample of UK ICU patients. Acta Anaesthesiol Scand 2008;52:202–208.

[2] Sjoding MW, Schoenfeld DA, Brown SM, Hough CL. Power calculations to select instruments for clinical trial secondary endpoints. a case study of instrument selection for post-traumatic stress symptoms in subjects with acute respiratory distress syndrome. Ann Am Thorac Soc. 2017 Jan;14(1):110-117.

[3] Parker AM, Nikayin S, Bienvenu OJ, Needham DM. Validity of the posttraumatic stress symptoms-14 instrument in acute respiratory failure survivors. Ann Am Thorac Soc. 2017 Jun;14(6):1047-1048.

[4] Rosendahl J, Kisyova H, Gawlytta R, Scherag A. Comparative validation of three screening instruments for posttraumatic stress disorder after intensive care. J Crit Care 2019; 53:149-54.

PCL-Specific (PCL-Specific)

1. Characteristics

The PCL-S is a 17-item evaluation scale created as a short version of the PCL-5, alongside the PCL-C [1]. It consists of 5 items for re-experiencing symptoms, 7 for emotional numbing and avoidance symptoms, and 5 for hyperarousal symptoms. Among questionnaire items, 8 items touch on a traumatic event. In these items, the PCL-C phrases it as “a stressful event in the past”, whereas the PCL-S prompts the respondent to specify the traumatic event and then refers to it as “that stressful event”.

2. Procedure and Method

The PCL-S is conducted using a questionnaire format. It typically takes approximately 5-10 minutes to complete. The scale has 17 items, and respondents rate each item on a scale of 1 to 5. The total score ranges from 17 to 85.

3. Other Information:

There is no fee for using the PCL-S. It generally has a diagnostic performance similar to that of the PCL-C, with scores of 28 or higher suggesting the presence of PTSD symptoms [1]. The concordance rate between diagnoses from PCL-S and PCL-5 was reported to be high at 91.3% [2].

References

[1] Wilkins KC, Lang AJ, Norman SB. Synthesis of the psychometric properties of the PTSD checklist (PCL) military, civilian, and specific versions. Depression and anxiety. 2011;28(7):596–606.

[2] Bovin MJ, Mahoney CT, Klein AB, Keane TM. Comparing the prevalence of probable DSM-IV and DSM-5 posttraumatic stress disorder in a sample of U.S. military veterans using the PTSD Checklist. Assessment. 2022 Nov 16:10731911221133483.

IES-6

1. Characteristics

The IES-6 is an evaluation scale for PTSD symptoms and is a short version of the 22-item IES-R, consisting of 6 items [1]. Each of the three symptoms included in DSM-IV – intrusion symptoms, avoidance symptoms, and hyperarousal symptoms – are evaluated by two items. Similar to the IES-R, it does not comply with DSM-5.

2. Procedure and Method

The IES-6 is conducted using a questionnaire format. Completion generally takes less than 5 minutes. In comparisons with the IES-R, the time required is shortened by 73%. Each of the 6 items is rated on a scale from 0 (not at all) to 4 (very much). Scoring methods use either the total score of the entire scale (0-24) or the average score (0-4). Previous studies reported that its sensitivity for diagnosing PTSD from trauma, natural disasters, and assaults ranged between 55 and 96%, with specificity of 74-99% [1].

3. Other Information:

There is no fee for using the IES-6. The IES-6, which has a high concordance rate with the IES-R, is a valid evaluation scale for screening PTSD in ARDS patients. A previous study suggested a cut-off of 1.75 [2]. A consensus conference in 2019 weakly recommended using the IES-R or IES-6 with a cut-off average score of 1.75 points to evaluate PTSD symptoms [3].

References

[1] Thoresen S, Tambs K, Hussain A, Heir T. Brief measure of posttraumatic stress reactions: Impact of event scale-6. soc psychiatry psychiatr epidemiol. 2010;45:405–12.

[2] Hosey MM, Leoutsakos JS, Li X, Dinglas VD. Screening for posttraumatic stress disorder in ARDS survivors: validation of the impact of event scale-6 (IES-6). Crit Care. 2019 Aug 7;23(1):276.

[3] Mikkelsen ME, Still M, Anderson BJ, Bienvenu OJ. Society of Critical Care Medicine's international consensus conference on prediction and identification of long-term impairments after critical illness. Crit Care Med. 2020 Nov;48(11):1670-1679.

PHQ-2

1. Characteristics

The PHQ was developed in the US as a screening tool for mental illnesses for primary care doctors [1]. The self-administered questionnaire, PHQ-9, is extracted from the PHQ, consisting of 9 questions related to major depression. To further save time, a scale focusing solely on two items (depressed mood and loss of pleasure) was developed as PHQ-2.

2. Procedure and Method

PHQ-2 is conducted using a questionnaire format. Completion generally takes approximately 1 minute. It consists of 2 items, with each being scored from 0-3 points, resulting in a total score of 0-6 points. A higher score indicates more severe depressive symptoms. Previous studies demonstrated that when diagnosing major depression using a PHQ-2 score cut-off of 2 points, sensitivity was 91% and specificity was 67%. With a cut-off of 3 points, sensitivity was 72% and specificity was 85% [2]. Combining PHQ-2 with PHQ-9 improved diagnostic performance. When the PHQ-2 score was 2 points or higher and PHQ-9 was conducted, resulting in a score of 10 or higher, sensitivity was 82% and specificity was 85%. However, diagnostic performance did not significantly differ between using only PHQ-9 (with a cut-off of 10 points for PHQ-9) and combining PHQ-2 with PHQ-9 [2].

3. Other Information:

Since Pfizer provides some language versions with free public access, there is no fee for using it in some countries. PHQ-2 showed good reliability (intraclass correlation coefficient = 0.92), and QOL, reflected by subscale scores for four WHO-QOL domains, was significantly lower in patients with increasing PHQ-2 scores, showing good construct validity [3].

References

[1] うつと不安のメンタルヘルスアセスメント（村松公美子著）

[2] Levis B, Sun Y, He C, Wu Y. Accuracy of the PHQ-2 alone and in combination with the PHQ-9 for screening to detect major depression: systematic review and meta-analysis. JAMA. 2020 Jun 9;323(22):2290-2300.

[3] Gelaye B, Wilson I, Berhane HY, Deyessa N, Bahretibeb Y, Wondimagegn D, Shibre Kelkile T, Berhane Y, Fann JR, Williams MA. Diagnostic validity of the Patient Health Questionnaire-2 (PHQ-2) among Ethiopian adults. Compr Psychiatry 2016; 70:216-21.

NRS (Numerical Rating Scale)

1. Characteristics

The NRS is primarily used to assess symptoms of anxiety, allowing patients to rate their anxiety on a scale of 0 (none at all) to 10 (the most intense anxiety imaginable).

2. Procedure and Method

The NRS is administered via a questionnaire. Completion typically takes less than 1 minute. While the NRS is a very simple measure of anxiety symptoms, a previous study indicated that its scores correlated with those of HADS-A [1]. In assessments of symptoms of anxiety in postpartum women, a cut-off score of 4 or higher suggested the presence or absence of anxiety as measured by HADS-A. The sensitivity of this cut-off was 70-80%, with specificity of 84-85% [2]. However, an optimal cut-off score has not been definitively established.

3. Other Information:

There is no fee for using the NRS. It was shown to correlate with State Trait Anxiety Inventory (STAI) (rho = 0.778, rho = 0.807) in a postpartum population [2].

References

[1] Prokopowicz A, Stańczykiewicz B, Uchmanowicz I. Anxiety and psychological flexibility in women after childbirth in the rooming-in unit during the COVID-19 pandemic. J Midwifery Womens Health. 2023 Jan;68(1):107-116.

[2] Prokopowicz A, Stanczykiewicz B, Uchmanowicz I. Validation of the numerical anxiety rating scale in postpartum females: a prospective observational study. Ginekol Pol. 2022;93(9):686-694.

Depression Anxiety Stress Scale (DASS)-21

1. Characteristics

DASS-21 is an evaluation scale for the symptoms of anxiety, depression, and stress [1]. It is a shortened version of the DASS, which has 42 items. DASS-21 is a validated measure for assessing the symptoms of both depression and anxiety. It evaluates the combined symptoms of depression and anxiety or evaluates these symptoms separately [2].

2. Procedure and Method

The scale consists of 21 items. Each item is rated on a 4-point scale from 0-3, with total scores ranging between 0 and 63 points. The questionnaire contains 7 items each for anxiety, depression, and stress, with scores for each symptom ranging between 0 and 21 points. A higher score indicates more severe symptoms. To diagnose depression using DASS-21, a cut-off score of 36 or higher for the total score showed sensitivity of 80.8% and specificity of 75.4% [3]. Regarding the depression subscore, a cut-off score of 12 or higher had sensitivity of 91% and specificity of 46% [4]. Other cut-off scores have been reported [5].

3. Other Information:

There is no fee for using DASS-21. A previous study verified the validity of DASS-21 among female university students [6].

References

[1] Lovibond PF, Lovibond SH. The structure of negative emotional states: comparison of the depression anxiety stress scales (DASS) with the Beck depression and anxiety inventories. Behav Res Ther. 1995 Mar;33(3):335-43.

[2] Antony, M. M., Bieling, P. J., Cox, B. J., Enns, M. W., Swinson, R. P. Psychometric properties of the 42-item and 21-item versions of the depression anxiety stress scales in clinical groups and a community sample. Psychological Assessment. 1998 10(2), 176-181.

[3] Nieuwenhuijsen K, de Boer AG, Verbeek JH, Blonk RW,. The depression anxiety stress scales (DASS): detecting anxiety disorder and depression in employees absent from work because of mental health problems. Occup Environ Med. 2003 Jun;60 Suppl 1(Suppl 1):i77-82.

[4] Tran TD, Tran T, Fisher J. Validation of the depression anxiety stress scales (DASS) 21 as a screening instrument for depression and anxiety in a rural community-based cohort of northern Vietnamese women. BMC Psychiatry. 2013 Jan 12;13:24.

[5] Guest R, Tran Y, Gopinath B, Cameron ID. Prevalence and psychometric screening for the detection of major depressive disorder and post-traumatic stress disorder in adults injured in a motor vehicle crash who are engaged in compensation. BMC Psychol. 2018 Feb 21;6(1):4.

[6] 女子大生を対象とした日本語版 The Depression Anxiety Stress Scales-21(DASS-21)の信頼性と妥当性の検討. 日本健康医学会雑誌 2022; 31: 380-389.

STAI

1. Characteristics

The STAI was developed in 1983 as an evaluation scale for anxiety symptoms [1]. Its validity for diagnosing anxiety symptoms and distinguishing them from depressive symptoms has been confirmed in clinical settings [2]. A major feature of the STAI is its ability to separately evaluate trait anxiety and state anxiety.

2. Procedure and Method

The STAI is a questionnaire that typically takes 10-15 minutes to complete. It consists of two subscales that evaluate trait anxiety and state anxiety, with each subscale containing 20 items. (Trait anxiety relates to an individual’s predisposition or likelihood to become anxious, with items such as “I tend to worry too much about insignificant things.”. State anxiety reflects how anxious a person feels at the moment, with items such as “I am feeling worried.”). Each item is scored from 1-4, resulting in a total score of 20-80 for each subscale. A higher score indicates stronger anxiety symptoms. Regarding state anxiety, a cut-off score of 39-40 was previously shown to detect clinically significant anxiety symptoms [3].

3. Other Information:

In Japan, the STAI is sold at 10,000 yen for 50 copies. Validity was good, and the internal consistency of the scale was excellent with Cronbach’s α value of 0.93 in gynecological settings [4]. Test-retest reliability was also good with an intra-class correlation coefficient of 0.80.

References

[1] Spielberger, C. D., Gorsuch, R. L., Lushene, R., Vagg, P. R. Manual for the State-Trait Anxiety Inventory. Palo Alto, CA: Consulting Psychologists Press; 1983.

[2] Spielberger, C. D. State-Trait Anxiety Inventory: Bibliography. Palo Alto, CA: Consulting Psychologists Press;1989.

[3] Knight RG, Waal-Manning HJ, Spears GF. Some norms and reliability data for the state-trait anxiety inventory and the Zung self-rating depression scale. Br J Clin Psychol 1983; 22 Pt 4: 245–9.

[4] Gustafson LW, Gabel P, Hammer A, Lauridsen HH, Petersen LK, Andersen B, Bor P, Larsen MB. Validity and reliability of State-Trait Anxiety Inventory in Danish women aged 45 years and older with abnormal cervical screening results. BMC Medical Research Methodology 2020;20(1):89.

Centre for Epidemiological Studies-Depression Scale (CES-D)

1. Characteristics

The CES-D was developed in 1977 to measure the severity of depressive symptoms [1]. Its validity as an indicator for evaluating depressive symptoms has been confirmed [2]. Although its initial purpose was to assess depressive symptoms in epidemiological research, its applicability as a screening tool for depression in primary care clinical settings and research was subsequently confirmed [1].

2. Procedure and Method

The CES-D is a questionnaire-based method that typically takes approximately 5-10 minutes to complete. It consists of 20 items that evaluate depressive mood and somatic symptoms, with each item being scored between 0-3 points. The total score ranges between 0 and 60 points. A score of 16 or higher is suggestive of depression [3]. When using a cut-off score of 16, sensitivity was 95% and specificity was 29% [1]. However, when a cut-off score of 20 was used, sensitivity was 93% and specificity was 92% [4].

3. Other Information:

In Japan, the CES-D is sold at 5,500 yen for 50 copies. There is also a version for children. Scores vary based on race. High internal consistency (Cronbach’s α value of 0.90) has been demonstrated [5].

References

[1] Radloff LS. The CES-D Scale: A self-report depression scale for research in the general population. Applied psychological measurement. 1977;1(3):385-401.

[2] Hann D, Winter K, Jacobsen P. Measurement of depressive symptoms in cancer patients: evaluation of the center for epidemiological studies depression scale (CES-D). J Psychosom Res. 1999;46(5):437-43.

[4] Lewinsohn PM, Seeley JR, Roberts RE, Allen NB. Center for epidemiological studies-depression scale (CES-D) as a screening instrument for depression among community-residing older adults. Psychology and Aging. 1997;12:277-87.

[5] Cosco TD, Prina M, Stubbs B, Wu YT. Reliability and validity of the center for epidemiologic studies depression scale in a population-based cohort of middle-aged U.S. Adults. J Nurs Meas 2017;5(3):476-85.

Visual Analogue Scale-Anxiety (VAS-A)

1. Characteristics

The VAS is a method that quantifies the intensity of various symptoms experienced by a patient and their subjective health status. A 10-cm straight line is typically used with the number 0 written on the left side and 100 on the right side. Patients mark on the line according to the intensity of the subjective symptoms they feel, which may be expressed in a range of 0-10 points. Psychological distress or anxiety symptoms, among other mental symptoms, may also be assessed using the VAS.

2. Procedure and Method

In an evaluation of the intensity of psychological distress, patients are instructed that “0 is no psychological distress” and “100 is the worst psychological distress ever experienced”, and they mark the line accordingly. The method is very simple, and generally takes less than 1 minute to fill in. Although a cut-off of 4 points is sometimes used, comprehensive studies to establish cut-offs for each symptom have not been thoroughly conducted. When evaluating intense psychological distress in cancer patients using a variation of VAS called the “distress thermometer”, and considering a total score of 15 or higher on the HADS as the gold standard, a VAS score of 4 or higher showed sensitivity of 87% and specificity of 73% [1].

3. Other Information:

There is no fee for using the VAS. It was previously employed to assess mental symptoms in a study on COVID-19 PICS [2]. The VAS has also been applied to assessments of mental symptoms (PICS-F) in family members [3]. Its validity was confirmed in screening for depression in cancer patients [4].

References

[1] Thalén-Lindström A, Larsson G, Hellbom M, Glimelius B. Validation of the distress thermometer in a Swedish population of oncology patients; accuracy of changes during six months. Eur J Oncol Nurs. 2013 Oct;17(5):625-31.

[2] Hatakeyama J, Inoue S, Liu K, Yamakawa K. Prevalence and risk factor analysis of post-intensive care syndrome in patients with covid-19 requiring mechanical ventilation: a multicenter prospective observational study. J Clin Med. 2022 Sep 28;11(19):5758.

[3] Suh J, Na S, Jung S, Kim KH, Choo S. Family caregivers' responses to a visitation restriction policy at a Korean surgical intensive care unit before and during the coronavirus disease 2019 pandemic. Heart Lung. 2023 Jan-Feb;57:59-64.

[4] Akizuki N, Yamawaki S, Akechi T, Nakano T. Development of an impact thermometer for use in combination with the distress thermometer as a brief screening tool for adjustment disorders and/or major depression in cancer patients. J Pain Symptom Manage. 2005 Jan;29(1):91-9.

Brief COPE (Coping Orientation to Problems Experienced) Inventory

1. Characteristics

The Brief COPE Inventory is a scale that was developed to measure how well someone is coping with a stressful event. It was created in 1997 as a shortened version of the COPE Inventory, which was developed in 1989 and comprises 60 items [1].

2. Procedure and Method

This is a questionnaire that takes approximately 10-15 minutes to complete. It consists of 28 items related to coping methods, and respondents are asked to rate each item on a scale of 1 (not coping at all) to 4 (coping a lot). Items assess coping strategies, such as “I’ve been turning to work or other activities to distract myself”, “I’ve been concentrating my efforts on doing something about the situation I’m in”, and “I’ve been telling myself this is not real”. Based on these responses, the degree to which each of the 14 coping strategies (e.g., self-distraction, active coping, and denial) is being used may be assessed [2].

3. Other Information:

There is no fee for using the Brief COPE Inventory. It showed good reliability and validity in women with breast cancer [3].

References

[1] Carver CS, Scheier MF, Weintraub JK. Assessing coping strategies: a theoretically based approach. J Pers Soc Psychol. 1989 Feb;56(2):267-83.

[2] Carver CS. You want to measure coping but your protocol's too long: consider the brief COPE. Int J Behav Med. 1997;4(1):92-100.

[3] Yusoff N, Low WY, Yip CH. Reliability and validity of the Brief COPE Scale (English version) among women with breast cancer undergoing treatment of adjuvant chemotherapy: a Malaysian study. Med J Malaysia 2010;65(1):41-4.

Trauma Screening Questionnaire (TSQ)

1. Characteristics

The TSQ is a scale that was developed to screen for PTSD [1]. It was created based on the PSS-SR (PTSD Symptom Scale-Self Report) scale [2]. The TSQ is consistent with the criteria set in DSM-IV.

2. Procedure and Method

It is a questionnaire method that takes approximately 5 minutes to complete. It consists of 10 items related to PTSD symptoms (5 of which are re-experiencing symptoms, while the other 5 are arousal symptoms). Respondents are asked if they experienced these symptoms more than twice in the past week. When using an interview-based diagnosis of PTSD as the gold standard, the presence of six or more symptoms indicates PTSD with a sensitivity of 76-86% and specificity of 93-97% [1]. The TSQ is designed to be used one month or more after experiencing a traumatic event, but not earlier. It is a screening tool, and individuals with a positive result are strongly advised to seek a diagnosis from a specialist. Further studies are needed to clarify how well the TSQ reflects the severity of PTSD symptoms.

3. Other Information:

There is no fee for using the TSQ. It is a valid screening tool for PTSD in patients with a psychotic disorder [3].

References

[1] Brewin CR, Rose S, Andrews B, Green J. Brief screening instrument for post-traumatic stress disorder. Br J Psychiatry. 2002 Aug;181:158-62.

[2] Foa, E.B., Riggs, D.S., Dancu, C.V., Barbara O. R. Reliability and validity of a brief instrument for assessing post-traumatic stress disorder. Journal of Traumatic Stress. 1993; 6, 459-473.

[3] de Bont PA, van den Berg DP, van der Vleugel BM, de Roos C, de Jongh A, van der Gaag M, van Minnen A. Predictive validity of the Trauma Screening Questionnaire in detecting post-traumatic stress disorder in patients with psychotic disorders. Br J Psychiatry 2015;206(5):408-16.

Major Depression Inventory (MDI)

1. Characteristics

The MDI is a scale that was developed by the WHO to diagnose and express the severity of major depression [1]. In addition to being usable as an aid for a clinical diagnosis in line with both DSM-IV and ICD-10, one of its distinguishing features is that it also assesses the intensity of symptoms [2].

2. Procedure and Method

It is a questionnaire method that takes approximately 5 minutes to complete. The inventory consists of 12 items, with respondents asked to rate the extent to which they have experienced each symptom over the past two weeks on a 6-point scale ranging from “not at all” to “always”. While there are 12 items, there are also combined questions, such as “Have you felt very restless?” with “Have you felt subdued or slowed down?” and “Have you been troubled by a decreased appetite?” with “Have you been troubled by an increased appetite?”, making it effectively 10 distinct questions. Therefore, scores range between 0 and 50, with higher scores indicating more severe depressive symptoms. A score of 21 or higher indicates mild depression, 26 or more moderate depression, and 31 or more severe depression.

3. Other Information:

There is no fee for using the MDI. The Chinese version of the MDI showed high reliability (Cronbach’s α value of 0.909, split-half reliability of 0.866) [3]. The MDI moderately correlated with GAD-7 (r = 0.425) and WHO-5 scores (r = −0.365).

References

[1] Bech P, Rasmussen N-A, Olsen LR, Noerholm V. The sensitivity and specificity of the major depression inventory, using the present state examination as the index of diagnostic validity. J Affect Disord 2001;66:159-64

[2] Olsen LR, Jensen DV, Noerholm V, Martiny K. The internal and external validity of the major depression inventory in measuring severity of depressive states. Psychol Med. 2003 Feb;33(2):351-6.

[3] Chen Y, Fang X, Shuai X, Fritzsche K, Leonhart R, Hoschar S, Li L, Ladwig K-H, Ma W, Wu H. Psychometric evaluation of the Major Depression Inventory (MDI) as a depression severity scale in Chinese patients with coronary artery disease. Findings From the MEDEA FAR-EAST Study. Frontiers in Psychiatry 2019;10.

PHQ-8

1. Characteristics

The PHQ was developed in the United States as a screening tool for mental disorders for primary care physicians [1]. PHQ-9 is a self-administered questionnaire extracted from the PHQ, consisting of nine questions related to major depression. PHQ-8 is derived from PHQ-9 by excluding item 9, which asks “Would you be better off dead or have you thought of hurting yourself in some way?”. In large-scale epidemiological studies, the use of PHQ-8 is sometimes preferred because of the difficulties associated with providing back-up support for “Yes” responses to item 9 and a “Yes” response not necessarily equating to an assessment of suicidal intent.

2. Procedure and Method

A questionnaire method is used that takes less than 5 minutes to complete. It consists of 8 items, with each ranging between 0-3 points, with a total possible score of 0-24. Higher scores indicate more severe depressive symptoms. PHQ-9 and PHQ-8 scores are roughly equivalent, and using the same cut-off (10 points or more) for PHQ-8 was shown to slightly reduce sensitivity and specificity, but not to a problematic extent [2].

3. Other Information:

Since Pfizer provides free public access, there is no fee for using PHQ-8 in some countries. Internal consistency reliability (Cronbach’s α value) was 0.82 with good validity and reliability in its use to measure depressive symptoms in patients with heart failure [3].

References

[1] うつと不安のメンタルヘルスアセスメント（村松公美子著）

[2] Wu Y, Levis B, Riehm KE, Saadat N. Equivalency of the diagnostic accuracy of the PHQ-8 and PHQ-9: a systematic review and individual participant data meta-analysis. Psychol Med. 2020 Jun;50(8):1368-80.

[3] Bjelland I, Dahl AA, Haug TT, Neckelmann D. The validity of the Hospital Anxiety and Depression Scale. An updated literature review. J Psychosom Res 2002;52(2):69-77.

PHQ-4

1. Characteristics

The PHQ was developed in the United States as a screening tool for mental disorders for primary care physicians [1]. PHQ-4 was designed for the ultra-convenient screening of both depression and anxiety disorders in primary care settings. It combines two items from the depression scale (PHQ-2) and two items from the generalized anxiety disorder scale (GAD-2) to create a 4-item scale.

2. Procedure and Method

A questionnaire method is used. It takes approximately 1-2 minutes to complete. The scale consists of 4 items, each scoring between 0-3 points, with a total possible score of 0-12. Higher scores indicate stronger mental symptoms (anxiety or depressive symptoms). The usefulness of the PHQ-4 for detecting any mental symptoms in primary care settings has been demonstrated [2]. In the GAD-2 part, scores of 2 or higher detect anxiety symptoms with sensitivity of 77% and specificity of 82%. In the PHQ-2 part, depression is detected with sensitivity of 87% and specificity of 85% [3].

3. Other Information:

Since Pfizer provides free public access, there is no fee for using PHQ-8 in some countries. PHQ-4 is a valid and reliable measure that may be applied to screen for depression and anxiety in the general population [4].

References

[1] うつと不安のメンタルヘルスアセスメント（村松公美子著）

[2] Kroenke K, Spitzer RL, Williams JB, Löwe B. An ultra-brief screening scale for anxiety and depression: the PHQ-4. Psychosomatics. 2009 Nov-Dec;50(6):613-21.

[3] Christodoulaki A, Baralou V, Konstantakopoulos G, Touloumi G. Validation of the patient health questionnaire-4 (PHQ-4) to screen for depression and anxiety in the Greek general population. J Psychosom Res. 2022 Sep;160:110970.

[4] Christodoulaki A, Baralou V, Konstantakopoulos G, Touloumi G. Validation of the Patient Health Questionnaire-4 (PHQ-4) to screen for depression and anxiety in the Greek general population. Journal of Psychosomatic Research 2022;160:110970.

Davidson Trauma Scale (DTS)

1. Characteristics

The DTS was developed in line with DSM-IV to assess the symptoms of PTSD [1]. It was designed with the intention to be used for PTSD screening and evaluating the severity of symptoms. It is used to establish whether symptoms align with the DSM-IV criteria for PTSD. A distinguishing feature of this scale is that it not only provides insights into the frequency and severity of symptoms, but also pinpoints which symptom category (re-experiencing symptoms, avoidance symptoms, and arousal symptoms) is most pronounced.

2. Procedure and Method

The scale is administered using a questionnaire method. It takes approximately 5-10 minutes to complete. It consists of 17 symptoms, with participants rating their frequency (from 0 for “not at all” to 4 for “every day”) and severity (from 0 for “not bothersome at all” to 4 for “extremely bothersome”) on a 5-point scale. Scores for frequency and symptom severity range between 0 and 68, resulting in a total score range of 0-136. The scale allows scores specific to re-experiencing, avoidance, and arousal symptoms to be defined by DSM-IV, providing insights into the severity of each type of symptom.

3. Other Information:

The DTS is sold in packs of 25 for USD $85. The manual costs $100. DTS showed good internal consistency, concurrent validity, and convergent and divergent validities for the diagnosis of PTSD in military veterans [2].

References

[1] Davidson, J. R. T., Book, S. W., Colket, J. T. Assessment of a new self-rating scale for post-traumatic stress disorder. Psychological Medicine. 1997; 27, 153-160.

[2] McDonald SD, Beckham JC, Morey RA, Calhoun PS. The validity and diagnostic efficiency of the Davidson Trauma Scale in military veterans who have served since September 11th, 2001. J Anxiety Disord 2009;23(2):247-55.

QOL, ADL, and Other Measurement Tests

- Barthel Index (BI)
- IADL (Instrumental Activities of Daily Living)
- mRS (modified Rankin Scale)
- Katz Activities of Daily Living
- Functional Independence Measure (FIM)
- WHODAS 2.0 (12-item World Health Organization Disability Assessment Schedule)
- Glasgow Outcome Scale (GOS)
- GOS-Extended (GOS-E)
- Cerebral Performance Category (CPC)
- Functional Activities Questionnaire (FAQ)
- Functional Performance Inventory (FPI)
- Zubrod score
- Disability Rating Scale (DRS)
- Short Form-36 Including RAND-36 (SF-36)
- EuroQol-5Dimension-5Level (EQ-5D-5L)
- EuroQol-5Dimension-3Level (EQ-5D-3L)
- EuroQol-Visual Analogue Scale (EQ-5D-VAS)
- SF-12
- World Health Organization Quality of Life- brief questionnaire (WHOQOL)
- Quality-Adjusted Life Year (QALY)
- Assessment of Quality of Life-4D (AQoL-4D)
- 15D instrument
- Pittsburgh Sleep Quality Index (PSQI)
- Insomnia Severity Index (ISI)
- Actigraphy
- Brief Pain Inventory
- Numerical Rating Scale (NRS)
- Visual Analogue Scale (VAS)
- Graded Chronic Pain Scale (GCPS)
- Fatigue Severity Scale (FSS)
- Fatigue Assessment Scale (FAS)
- Measure of Current Status part A (MOCS-A)
- General Self-Efficacy (GSE) Scale
- Family Satisfaction in the ICU (FS-ICU)
- Quality of Death and Dying (QODD)
- Zarit Burden Interview-12 items (Zarit-12)

Barthel Index (BI)

1. Characteristics

The BI is a quantitative tool designed to evaluate ADL in individuals with physical disabilities. It is widely used in rehabilitation programs for patients with conditions such as stroke, neurological disorders, and orthopedic diseases [1].

2. Procedure and Method

The evaluation involves reports from medical staff or family members, direct observations, or interviews with the patients themselves. The index evaluates ten items: eating, grooming, mobility, toileting, bathing, ascending and descending stairs, moving with the assistance of a wheelchair or walker, dressing, urinary management, and bowel management. Each item is scored differently based on the level of independence or the need for assistance, with the highest possible score being 100 points. The scores for each item are summed to calculate the total score. A higher score indicates a greater level of independence for the patient.

3 Other Information

No fees are required for using the BI. It has high reliability across different healthcare professions and is useful for developing care and rehabilitation plans for patients [2].

References

[1] Granger CV, Dewis LS, Peters NC, Sherwood CC. Stroke rehabilitation: analysis of repeated Barthel index measures. Arch Phys Med Rehabil. 1979 Jan;60(1):14-7.

[2] Collin C, Wade DT, Davies S, Horne V. The Barthel ADL Index: a reliability study. Int Disabil Stud. 1988;10(2):61-3.

Instrumental Activities of Daily Living (IADL)

1. Characteristics

IADL, also referred to as the Lawton Instrumental Activities of Daily Living Scale, serves as a metric for evaluating the independence of elderly or disabled individuals in their daily lives [1]. IADL encompasses more intricate and social daily living tasks, which are distinct from basic ADL. IADL typically consists of eight items: telephone usage, shopping, cooking, household chores, laundry, transportation, medication management, and financial management.

2. Procedure and Method

Evaluators conduct interviews with patients, asking questions about each IADL item. These inquiries revolve around the extent to which a patient independently performs each task. Based on the patient’s responses, evaluators assign scores to each item. Scores are typically assessed on a three-tier scale corresponding to the level of independence: independent, partially independent, and dependent. The scores of individual items are summed to obtain the patient’s overall IADL score. This score serves as an indicator of the patient’s independence in daily life. Using the IADL score, caregivers and professionals may confirm the patient’s care needs, set rehabilitation objectives, and devise appropriate support and treatment plans.

3 Other Information

No fees are required for using IADL. Its reliability and validity in older adults were r = 0.928 and r = 0.793, respectively [2].

References

[1] Lawton MP, Brody EM. Assessment of older people: self-maintaining and instrumental activities of daily living. Gerontologist. 1969 Autumn;9(3):179-86.

[2] Zhang Y, Liu P, Pan Y, Li Y, Zhang L, Li Y, Ma L. Reliability and validity of the function impairment screening tool in Chinese older adults. Front Med (Lausanne) 2021;8:720607.

modified Rankin Scale (mRS)

1. Characteristics

The mRS is primarily a measure that assesses functional impairments and recovery in stroke patients [1]. The mRS is a 6-level scale ranging from 0 (no symptoms) to 5 (severe disability requiring assistance), with a commonly added 7th level, 6, indicating death.

2. Procedure and Method

Information related to a patient’s ADL and cognitive function is collected, which may include reports from medical staff or family, direct observations, or interviews with the patient themselves. Based on this information, the patient’s functional impairment is classified on a 6-level scale from 0 (no symptoms) to 5 (severe disability requiring assistance). Death is scored as 6. Using the categories of the mRS, the patient’s functional outcomes and the effectiveness of rehabilitation are evaluated.

3 Other Information

No fees are required for using the mRS. This scale is widely employed to gauge the outcomes of stroke treatments and the efficacy of rehabilitation, and is considered to have high reliability and validity [2-3].

References

[1] van Swieten JC, Koudstaal PJ, Visser MC, Schouten HJ. Interobserver agreement for the assessment of handicap in stroke patients. Stroke. 1988 May;19(5):604-7.

[2] Wilson JT, Hareendran A, Hendry A, Potter J. Reliability of the modified rankin scale across multiple raters: benefits of a structured interview. Stroke. 2005 Apr;36(4):777-81.

[3] Quinn TJ, Lees KR, Hardemark HG, Dawson J. Initial experience of a digital training resource for modified rankin scale assessment in clinical trials. Stroke. 2007 Aug;38(8):2257-61.

Katz Activities of Daily Living (ADL)

1. Characteristics

Katz ADL is an index that assesses the ability of the elderly or those with disabilities to perform basic ADL [1]. Katz ADL focuses on six basic daily activities that a patient may independently perform [2]. These activities are bathing, dressing, using the toilet, transferring (such as moving from the bed to a chair), controlling urinary and fecal incontinence, and eating.

2. Procedure and Method

The evaluator interviews the patient and asks questions related to each item of Katz ADL. Questions assess the extent to which the patient independently performs each task. Based on the patient’s responses, the evaluator assigns scores to each item. Scores are typically evaluated in two levels based on the degree of independence: “independent” and “dependent”. The scores for each item are totaled to obtain the overall Katz ADL score for the patient. This score is used as an indicator of the patient’s independence in daily living. The Katz ADL score provides important information on the care needs of and rehabilitation goals for a patient, according to which appropriate support and treatment plans are developed.

3 Other Information

No fees are required for using Katz ADL. Test-retest and inter-rater reliabilities were excellent (ICC 0.999 [95% CI 0.999-1.000]). Regarding convergent validity, strong associations were observed between Katz ADL, BI, and SF-36 PF (rs=0.988, p<0.001 and rs=0.674, p<0.001).

References

[1] Katz S, Ford AB, Moskowitz RW, Jackson BA. Studies of illness in the aged: The index of ADL: A standardized measure of biological and psychosocial function. JAMA 1963;185(12):914-9.

[2] LaPlante MP. The classic measure of disability in activities of daily living is biased by age but an expanded IADL/ADL measure is not. J Gerontol B Psychol Sci Soc Sci. 2010;65(6):720-32.

[3] Arik G, Varan HD, Yavuz BB, Karabulut E, Kara O, Kilic MK, Kizilarslanoglu MC, Sumer F, Kuyumcu ME, Yesil Y. Validation of Katz index of independence in activities of daily living in Turkish older adults. Arch Gerontol Geriatr 2015;61(3):344-50.

Functional Independence Measure (FIM)

1. Characteristics

The FIM is a comprehensive tool that is used to assess ADL and cognitive functions in individuals with physical disabilities [1]. Comprising 18 items, the FIM covers aspects such as ADL, mobility, communication, and social cognitive functions. It is particularly useful for gauging the effectiveness of rehabilitation due to its reliability and quick and easy assessment process [2]. Its consistency across various medical professionals makes it invaluable for devising patient care and rehabilitation plans [3].

2. Procedure and Method

Information is collected on a patient’s ADL and cognitive functions using reports from medical staff or family members, direct observations, and patient interviews. The 18 items evaluated are as follows: eating, grooming, mobility, toileting, bathing, dressing, urinary management, bowel management, transferring (bed/wheelchair/toilet), stair climbing, comprehension, expression, social interaction, problem-solving, memory, and remote functioning. Each item is rated on a scale of 1 to 7 based on the level of independence or need for assistance. The scores for each item are summed to derive the total score. A higher score indicates greater independence.

3 Other Information

There is no fee for using the FIM. Interrater reliability was moderate with an intraclass correlation coefficient of 0.83 [4]. Construct validity to the Expanded Disability Status Scale was r = -0.907. Cronbach’s α value was 0.94. The minimally important clinical difference was 44 in chronically ventilated patients with rehabilitation [5].

References

[1] Ottenbacher KJ, Hsu Y, Granger CV, Fiedler RC. The reliability of the functional independence measure: a quantitative review. Arch Phys Med Rehabil. 1996 Dec;77(12):1226-32.

[2] Stineman MG, Shea JA, Jette A, Tassoni CJ. The functional independence measure: tests of scaling assumptions, structure, and reliability across 20 diverse impairment categories. Arch Phys Med Rehabil. 1996 Nov;77(11):1101-8.

[3] Linacre JM, Heinemann AW, Wright BD, Granger CV. The structure and stability of the functional independence measure. Arch Phys Med Rehabil. 1994 Feb;75(2):127-32.

[4] Brosseau L, Wolfson C. The inter-rater reliability and construct validity of the Functional Independence Measure for multiple sclerosis subjects. Clinical Rehabilitation 1994; 8:107-15.

[5] Chen S, Su CL, Wu YT, Wang LY, Wu CP, Wu HD, Chiang LL. Physical training is beneficial to functional status and survival in patients with prolonged mechanical ventilation. J Formos Med Assoc 2011; 110(9):572-9.

12-item World Health Organization Disability Assessment Schedule

(WHODAS 2.0)

1. Characteristics

The WHODAS 2.0 is a disability assessment schedule developed by the World Health Organization (WHO) [1]. It is used to measure the extent of impairments related to physical functions, physical activities, and social participation. This assessment tool is versatile and may be applied across various cultural and disease contexts.

2. Procedure and Method

The assessor interviews a patient about each item, with a focus on the patient’s activities and functional status in the past 30 days. The patient rates the extent of difficulties experienced for each item on a 5-point scale (1 = no difficulty at all, 5 = extremely difficult). Scores from each item are summed to derive the patient’s overall disability score. The assessment covers the following six domains: cognitive function (understanding & decision-making), mobility (movement & lifting), self-care (ADL), interpersonal relationships (social interactions), life activities (domestic & leisure activities), and social participation (fulfillment of social roles).

3 Other Information

There is no fee for using the WHODAS 2.0. Cronbach’s α value for the scale was 0.94, suggesting good internal consistency reliability [2]. There is evidence for convergent validity because WHODAS moderately correlated with other general measures of health-related QOL.

References

[1] Üstün TB, Kostanjsek N, Chatterji S, Rehm J. Measuring health and disability: Manual for WHO disability assessment schedule WHODAS 2.0. World Health Organization. 2010.

[2] Carlozzi NE, Kratz AL, Downing NR, Goodnight S, Miner JA, Migliore N, Paulsen JS. Validity of the 12-item World Health Organization Disability Assessment Schedule 2.0 (WHODAS 2.0) in individuals with Huntington disease (HD). Qual Life Res 2015;4(8):1963-71.

Glasgow Outcome Scale (GOS)

1. Characteristics

The GOS is used to assess the prognosis of patients with brain injuries [1]. It is employed to measure the long-term functional outcomes of patients with traumatic brain injuries and stroke. It is a concise and comprehensive evaluation that is widely used both clinically and in research settings.

2. Procedure and Method

Based on a patient’s medical records, interviews, and observations, the assessor evaluates the patient’s current functional status. The assessor classifies the patient’s functional status into one of the five GOS categories and then selects the most appropriate category. GOS categories are used to assess the patient’s prognosis, set rehabilitation goals, and evaluate the efficacy of treatments.

3 Other Information

There is no fee for using the GOS. κ values for test-retest and interrater reliabilities were 0.92 and 0.85, respectively [2].

References

[1] Jennett, B., Bond, M. Assessment of outcome after severe brain damage: A practical scale. Lancet. 1975; 305(7905), 480-484.

[2] Pettigrew LE, Wilson JT, Teasdale GM. Reliability of ratings on the Glasgow Outcome Scales from in-person and telephone structured interviews. J Head Trauma Rehabil 2003;18(3):252-8.

GOS-Extended (GOS-E)

1. Characteristics

The GOS-E is an extended scale designed to evaluate the functional outcomes of patients with brain injuries [1]. It allows for a more detailed and comprehensive assessment than the GOS, providing important information on the prognosis and progress of rehabilitation in brain-injured patients.

2. Procedure and Method

Based on a patient’s medical records, interviews, and observations, the assessor assesses the patient’s current functional status. The assessor then classifies the patient’s functional status into one of the eight GOS-E categories and selects the most appropriate category. These GOS-E categories are employed to evaluate the patient’s prognosis, set rehabilitation goals, and assess the efficacy of treatments.

3 Other Information

There is no fee for using the GOS-E. κ values for test-retest and interrater reliabilities were 0.92 and 0.84, respectively [2].

References

[1] Wilson, J. T., Pettigrew, L. E., Teasdale, G. M. Structured interviews for the Glasgow Outcome Scale and the extended Glasgow Outcome Scale: Guidelines for their use. Journal of Neurotrauma. 1998;15(8),573-585.

[2] Pettigrew LE, Wilson JT, Teasdale GM. Reliability of ratings on the Glasgow Outcome Scales from in-person and telephone structured interviews. J Head Trauma Rehabil 2003;18(3):252-8.

Cerebral Performance Category (CPC)

1. Characteristics

The CPC is a simple classification system that was mainly designed to assess the recovery of brain functions. It is widely used, particularly when evaluating the neurological outcomes of patients after cardiac arrest [1]. The CPC consists of five categories. This scale is helpful for assessing functional recovery and the outcome of rehabilitation [2].

2. Procedure and Method

Information is collected on a patient’s neurological condition using reports from medical staff, direct observations, and interviews or surveys with the patient themselves. Based on the information obtained, the patient’s brain function is classified into one of five categories (CPC 1–5). CPC categories are then used to evaluate the neurological outcome and efficacy of the patient’s rehabilitation. The CPC may be assessed quickly and simply, making it extremely useful for measuring the neurological outcome and effects of rehabilitation in patients after cardiac arrest [3]. It also has high reliability among different medical practitioners, assisting in the formulation of patient care and rehabilitation plans [4].

3 Other Information

There is no fee for using the CPC. In a cohort of survivors of out-of-hospital ventricular fibrillation cardiac arrest, the inter- and intra-reviewer agreement of the CPC to classify favorable versus unfavorable neurological statuses at hospital discharge varied [5].

References

[1] Brain Resuscitation Clinical Trial I Study Group. Randomized clinical study of thiopental loading in comatose survivors of cardiac arrest. New England Journal of Medicine. 1986;314(7),397-403.

[2] Jennett B, Bond M. Assessment of outcome after severe brain damage: a practical scale. Lancet. 1975; 1(7905),480-484.

[3] Laver S, Farrow C, Turner D, Nolan J. Mode of death after admission to an intensive care unit following cardiac arrest. Intensive Care Medicine. 2004;30(11),2126-2128.

[4] Raina KD, Callaway C, Rittenberger JC, Holm MB. Neurological and functional status following cardiac arrest: method and tool utility. Resuscitation. 2008;79(2):249-56.

[5] Ajam K, Gold LS, Beck SS, Damon S, Phelps R, Rea TD. Reliability of the Cerebral Performance Category to classify neurological status among survivors of ventricular fibrillation arrest: a cohort study. Scand J Trauma Resusc Emerg Med 2011;19(1):38.

Functional Activities Questionnaire (FAQ)

1. Characteristics

The FAQ is a scale that is used to evaluate the functional daily activities of elderly individuals with dementia or MCI [1]. It consists of 10 items and assesses whether the elderly have the ability to perform ADL independently. The FAQ provides information on the impact of cognitive decline on ADL and assists in planning appropriate care and treatment.

2. Procedure and Method

The evaluator asks a patient’s family or caregivers about the extent to which the patient independently performs daily living tasks. The FAQ includes daily living tasks, such as shopping, cooking, and money management.

The family or caregivers rate each item on a 4-point scale based on the difficulty experienced by the patient (0 = normal activity, 1 = has difficulty, but performs independently, 2 = needs assistance, 3 = hardly does it).

The scores for each item are totaled to calculate the patient’s overall functional score. A higher score indicates a more severe functional decline. Using the functional score, the patient’s care needs and rehabilitation goals are assessed, and an appropriate support or treatment plan is formulated.

3 Other Information

There is no fee for using the FAQ. There is strong evidence for its reliability (0.52–0.95) and it strongly correlated with neurocognitive measures (rs: −0.30 to −0.59) [2].

References

[1] Pfeffer RI, Kurosaki TT, Harrah CH Jr, Chance JM, Filos S. Measurement of functional activities in older adults in the community. J Gerontol. 1982 May;37(3):323-9.

[2] González DA, Gonzales MM, Resch ZJ, Sullivan AC, Soble JR. Comprehensive evaluation of the Functional Activities Questionnaire (FAQ) and its reliability and validity assessment 2022;29(4):748-63.

Functional Performance Inventory (FPI)

1. Characteristics

The FPI is a scale that was designed to assess the functional performance of patients with chronic diseases. It consists of 37 items spanning 6 domains, evaluating a patient’s daily activities, work, household roles, social activities, self-management, and cognitive functions. This scale is useful for understanding the functional performance of chronic disease patients, evaluating treatment efficacy, and setting rehabilitation goals [1].

2. Procedure and Method

The evaluator asks a patient about their functional performance in the past 4 weeks. Questions relate to six domains: daily activities, work, roles within the household, social activities, self-management, and cognitive functions. Patients rate each item on a 5-point scale based on the difficulty they experience (1 = very difficult, 5 = not difficult at all). The scores for each item are totaled to calculate a patient’s overall functional score. A higher score indicates better functional performance.

3 Other Information

There is no fee for using the FPI. Construct validity was supported by correlations with the Medical Outcomes Study Short Form-36, Physical Functioning (r = 0.69), the Physical Activity Scale for the Elderly (r = 0.62), and American Thoracic Society-Division of Lung Disease Breathlessness scale (r = −0.62) [2].

References

[1] Whiteneck GG, Charlifue SW, Gerhart KA, Overholser JD, Richardson GN. Quantifying handicap: A new measure of long-term rehabilitation outcomes. Archives of Physical Medicine and Rehabilitation. 1992; 73(6), 519-526.

[2] Larson JL, Kapella MC, Wirtz S, Covey MK, Berry J. Reliability and validity of the functional performance inventory in patients with moderate to severe chronic obstructive pulmonary disease. J Nurs Meas 1998;6(1):55-73.

Zubrod score

1. Characteristics

The Zubrod score, also known as the Eastern Cooperative Oncology Group Performance Status, is a scale that is used to evaluate the general functional status and performance of cancer patients. It is composed of five levels and holistically evaluates a patient’s activity level, self-care ability, and severity of symptoms. The Zubrod score is useful for devising treatment plans, prognostic judgments, evaluating treatment effects, and assessing eligibility for clinical trials [1].

2. Procedure and Method

The evaluator collects information on the patient’s general functional status, activity level, self-care ability, and severity of symptoms. The evaluator then classifies the patient’s condition on a 5-level scale. Using the Zubrod score, treatment plans are devised, prognostic judgments are reached, treatment effects are evaluated, and eligibility for clinical trials is assessed.

3 Other Information

There is no fee for its use. It provides vital information about a patient’s prognosis and treatment adaptability and is widely used in clinical research [2]. This score is considered to be an effective method for evaluating the impact of a patient’s health condition on treatment outcomes and survival duration [3].

References

[1] Zubrod CG, Schneiderman M, Frei E, Brindley C. Appraisal of methods for the study of chemotherapy of cancer in man: comparative therapeutic trial of nitrogen mustard and triethylene thiophosphoramide. Journal of Chronic Diseases. 1960;11(1),7-33.

[2] Buccheri G, Ferrigno D, Tamburini M. Karnofsky and ECOG performance status scoring in lung cancer: a prospective, longitudinal study of 536 patients from a single institution. European Journal of Cancer. 1996; 32(7),1135-1141.

[3] Oken MM, Creech RH, Tormey DC, Horton J, Davis TE. Toxicity and response criteria of the Eastern Cooperative Oncology Group. American Journal of Clinical Oncology. 1982;5(6),649-655.

Disability Rating Scale (DRS)

1. Characteristics

The DRS is used to evaluate the extent of disability and the recovery process in patients with brain injuries [1]. This scale consists of eight items that holistically evaluate levels of consciousness, cognitive function, dependency, and adaptability. The DRS is beneficial for devising rehabilitation plans, evaluating treatment effects, reaching prognostic judgments, and collecting clinical data for research.

2. Procedure and Method

The evaluator collects information on a patient’s level of consciousness, cognitive function, dependency, and adaptability. The evaluator then assesses the extent of the patient’s impairment for each item. The evaluation scale varies by item; however, a higher score typically indicates a more severe disability. The scores for each item are totaled to calculate the patient’s overall disability score. A higher score indicates a more severe disability.

3 Other Information

There is no fee for using the DRS. It has good reliability and validity [2]. The inter-rater reliability of the DRS was previously reported to range between 0.93 and 0.98, while its validity ranged between 0.67 and 0.80 for the GOS and was 0.85 for the GOS-E.

References

[1] Rappaport M, Hall KM, Hopkins K, Belleza T. Disability rating scale for severe head trauma: coma to community. Arch Phys Med Rehabil. 1982;63(3):118-23.

[2] Deepika A, Devi BI, Shukla D. Predictive validity of disability rating scale in determining functional outcome in patients with severe traumatic brain injury. Neurol India 2017;65(1):83-6.

SF-36 (Short Form-36) Including RAND-36

1. Characteristics

SF-36 is a generic scale that is used to evaluate the general health status and QOL. RAND-36 is a version of SF-36 developed by RAND (Research ANd Development) [1]. SF-36 comprises eight health conceptual domains, with each evaluating information related to a patient’s physical health, mental health, role function, social function, emotional function, mental health, pain, and general health. SF-36 is widely used for patients with various diseases and in different age groups and facilitates evaluations of treatment effects, epidemiological surveys, and outcome evaluations in clinical trials.

2. Procedure and Method

The evaluator asks a patient questions about their health status in the past four weeks. Questions relate to eight health conceptual domains. Patients evaluate how much of a problem they felt for each item. The evaluation scale varies by item, but typically uses a 3- to 6-point scale. The scores of each item are aggregated, and a score is calculated for each domain. The score for each domain ranges between 0 and 100, with a higher score indicating a better health status.

3 Other Information

There is a fee for using SF-36. Depending on the frequency of use, it costs approximately 100,000 yen. High validity and reliability have been confirmed in research on various populations [2, 3].

References

[1] Ware JE Jr, Sherbourne CD. The MOS 36-item short-form health survey (SF-36). I. Conceptual framework and item selection. Med Care. 1992 Jun;30(6):473-83.

[2] Chrispin PS, Scotton H, Rogers J, Lloyd D, Ridley SA. Short Form 36 in the intensive care unit: assessment of acceptability, reliability and validity of the questionnaire. Anaesthesia 1997;52(1):15-23.

[3] Orwelius L, Fredrikson M, Kristenson M, Walther S, Sjöberg F. Health-related quality of life scores after intensive care are almost equal to those of the normal population: a multicenter observational study. Crit Care 2013;17(5):R236.

EuroQol-5Dimension-5Level (EQ-5D-5L)

1. Characteristics

EQ-5D-5L is a shortened self-reported questionnaire that was developed to evaluate the health status and QOL of patients [1]. It covers five health domains (mobility, self-care, usual activities, pain/discomfort, and anxiety/depression) with five levels of evaluation set for each domain.

2. Procedure and Method

The EQ-5D-5L questionnaire is distributed to patients, who then answer questions on the five health domains. The evaluation level for each domain ranges between 1 (no problems) and 5 (severe problems).　Responses to the questionnaire are collected and the score for each of the five health domains is calculated. This creates a profile of a patient’s health status. Regarding the EQ-5D-5L utility index, the weighting coefficient calculated in each country is applied to convert the patient’s health status into a single numerical value. The patient’s health status and QOL are evaluated and the effects of interventions or treatments are measured.

3 Other Information

There is no fee for using EQ-5D-5L. This tool is widely applied to measure the health status of patients with various diseases and health conditions [2]. The discriminative ability, test-retest reliability, and construct validity of EQ-5D-5L are good [3].

References

[1] Herdman M, Gudex C, Lloyd A, Janssen M. Development and preliminary testing of the new five-level version of EQ-5D (EQ-5D-5L). Qual Life Res. 2011 Dec;20(10):1727-36.

[2] Janssen MF, Pickard AS, Golicki D, Gudex C. Measurement properties of the EQ-5D-5L compared to the EQ-5D-3L across eight patient groups: a multi-country study. Qual Life Res. 2013 Sep;22(7):1717-27.

[3] Dams J, Rimane E, Steil R, Renneberg B, Rosner R, König H-H. Reliability, validity and responsiveness of the EQ-5D-5L in assessing and valuing health status in adolescents and young adults with posttraumatic stress disorder: a Randomized Controlled Trail. Psychiatr Q 2021;92(2):459-71.

EuroQol-5Dimension-3Level (EQ-5D-3L)

1. Characteristics

EQ-5D-3L is a shortened self-report questionnaire that was developed to evaluate the health status and QOL of patients [1]. It covers five health domains (mobility, self-care, usual activities, pain/discomfort, and anxiety/depression) with three levels of evaluation set for each domain. This tool is widely applied to measure the health status of patients with various diseases and health conditions [2].

2. Procedure and Method

The EQ-5D-3L questionnaire is distributed to patients, who then answer questions on the five health domains. The evaluation level for each domain ranges between 1 (no problems) and 3 (severe problems).

Responses to the questionnaire are collected and the score for each of the five health domains is calculated. This creates a profile for a patient’s health status. The patient’s health status and QOL are evaluated and the effects of interventions or treatments are measured.

3 Other Information

There is no fee for using EQ-5D-3L. Its reliability was ICC of 0.52 to 0.83 [3] and validity was 0.55 [4]. Internal consistency was Cronbach’s α value of 0.87 [4].

References

[1] EuroQol Group. EuroQol--a new facility for the measurement of health-related quality of life. Health Policy. 1990;16(3):199-208.

[2] Brooks R. EuroQol: the current state of play. Health Policy. 1996;37(1):53-72.

[3] Buchholz I, Janssen MF, Kohlmann T, Feng YS: A Systematic Review of Studies Comparing the Measurement Properties of the Three-Level and Five-Level Versions of the EQ-5D. Pharmacoeconomics 2018; 36(6):645-61.

[4] Zare F, Ameri H, Madadizadeh F, Aghaei MR. Validity and reliability of the EQ-5D-3L (a generic preference-based instrument used for calculating quality-adjusted life -years) for patients with type 2 diabetes in Iran. Diabetes Metab Syndr 2021;15(1):319-24.

EuroQol-Visual Analogue Scale (EQ-5D-VAS)

1. Characteristics

EQ-5D-VAS is a VAS that was developed to evaluate the health status and QOL of patients [1]. It is a part of the health-related QOL evaluation tool developed by the EuroQol Group, similar to EQ-5D-3L and EQ-5D-5L. It allows patients to subjectively evaluate their current health status.

2. Procedure and Method

A piece of paper on which the EQ-5D-VAS scale is drawn is given to a patient. This scale depicts a range from 0 (worst health status) to 100 (best health status). The patient marks a point on the scale that corresponds to their current health status. The numerical value of the point marked by the patient is recorded and used to evaluate the patient’s health status and QOL.

3 Other Information

There is no fee for using EQ-5D-VAS. Its reliability was ICC 0.92 and validity with SF-36 was 0.42–0.70 [2]

References

[1] EuroQol Group. EuroQol--a new facility for the measurement of health-related quality of life. Health Policy. 1990 Dec;16(3):199-208.

[2] Khoudri I, Belayachi J, Dendane T, Abidi K, Madani N, Zekraoui A, Zeggwagh AA, Abouqal R. Measuring quality of life after intensive care using the Arabic version for Morocco of the EuroQol 5 Dimensions. BMC Res Notes 2012;5:56.

SF-12

1. Characteristics

SF-12 is a condensed scale that was designed to evaluate the general health status and QOL of patients and is derived from SF-36 [1]. This scale consists of 12 questions and evaluates information related to a patient’s physical health, mental health, role functioning, social functioning, emotional wellbeing, mental health, pain, and general health.

2. Procedure and Method

The evaluator asks patients questions about their health status in the past 4 weeks. Questions are divided into items related to physical and mental health. Patients evaluate the extent to which they felt issues for each item. Although the evaluation scale varies depending on the item, a scale of 3-6 levels is typically used. Scores for each item are aggregated, and two summary scores, one for physical health and another for mental health, are calculated. Each summary score is represented within a range of 0-100, with a higher score indicating a better health status. These scores are used to evaluate the patient’s health status and QOL and also develop appropriate support or treatment plans.

3 Other Information

There is a fee for using SF-12. It is slightly more affordable than SF-36. The test-retest reliability of SF-12 summary measures was 0.890 in the United States and 0.864 in the United Kingdom [1]. Internal consistency based on Cronbach’s α value was 0.84 for PCS items and 0.81 for MCS items [2].

References

[1] Ware J Jr, Kosinski M, Keller SD. A 12-Item short-form health survey: construction of scales and preliminary tests of reliability and validity. Med Care. 1996 Mar;34(3):220-33.

[2] Lim LL, Fisher JD. Use of the 12-item short-form (SF-12) Health Survey in an Australian heart and stroke population. Qual Life Res 1999;8(1-2):1-8.

World Health Organization Quality of Life-brief questionnaire

(WHOQOL)

1. Characteristics

The WHOQOL is an internationally recognized tool for measuring QOL, with WHOQOL-brief being its shortened version [1]. This scale consists of 24 items and evaluates information related to a patient’s QOL in four domains (physical health, psychological health, social relationships, and environment). WHOQOL-brief is widely used for patients with various diseases and from different cultural backgrounds. It is useful for evaluating treatment effects, epidemiological surveys, and clinical trial outcome assessments.

2. Procedure and Method

The evaluator asks a patient questions about their health status and QOL in the past 2 weeks. Questions relate to the four domains (physical health, psychological health, social relationships, and environment). Patients evaluate the extent to which they felt issues for each item. A 5-level Likert scale is used for the evaluation. Scores for each item are aggregated and scores for each domain are calculated. Scores for each domain are represented in a range of 4-20, with a higher score indicating better QOL. Using these scores, the patient’s QOL is evaluated and an appropriate support or treatment plan is developed.

3 Other Information

There is no fee for using the WHOQOL. In healthy student volunteers, Cronbach’s α value was 0.896 [2]. Internal reliability was higher than 0.70. Test–retest reliability demonstrated the good stability of the scale.

References

[1] Development of the World Health Organization WHOQOL-BREF quality of life assessment. The WHOQOL Group. Psychol Med. 1998;28(3):551-8.

[2] Ilić I, Šipetić S, Grujičić J, Mačužić I, Kocić S, Ilić M. Psychometric properties of the World Health Organization's Quality of Life (WHOQOL-BREF) questionnaire in medical students. Medicina (Kaunas) 2019;55(12).

Quality-Adjusted Life Year (QALY)

1. Characteristics

QALY is an indicator that is used to evaluate the effects of medical interventions and treatment programs. It measures a patient’s quality of life combined with its duration [1]. QALY represents how much time a patient spends in a healthy state and is useful for the effective allocation of medical resources and a cost-effectiveness analysis between different treatment methods.

2. Procedure and Method

Information is collected on a patient’s health status and their QOL is evaluated. This may involve using measurement tools such as EQ-5D-5L or SF-36. QOL is converted into an index value (utility value) ranging between 0 and 1. 0 represents death, while 1 represents a perfect state of health. The period (in years) that the patient maintains a good health status is evaluated. The utility value is multiplied by the duration to calculate QALY. For example, if the utility value is 0.8 and a good health status is maintained for 5 years, QALY is 0.8 × 5 = 4.

3 Other Information

There is no fee for using QALY. It has limitations for producing reliable and valid measurements across disease categories and does not consider a number of contextual factors, including program specificity, palliative care, or mental status [2]. Furthermore, QALY does not cover the nuances needed within and across disease categories and patients.

References

[1] Weinstein MC, Torrance G, McGuire A. QALYs: the basics. Value Health. 2009;12 Suppl 1:S5-9.

[2] Pettitt D, Raza S, Naughton B, Roscoe A, Ramakrishnan A, Ali A, Davies B, Dopson S, Hollander G, Smith J. The limitations of QALY: a literature review. Journal of Stem Cell Research and Therapy 2016;6(4).

Assessment of Quality of Life-4D (AQoL-4D)

1. Characteristics

AQoL-4D is a multi-dimensional tool that was developed by Australian researchers to measure a patient’s QOL [1]. This scale consists of 12 questions and assesses information related to QOL in four domains: physical health, mental health, social relationships, and independence. AQoL-4D is used across a range of diseases and age groups.

2. Procedure and Method

The evaluator asks a patient questions about their health and QOL in the past week. Questions relate to the four domains: physical health, mental health, social relationships, and independence. Patients assess how much of a problem they perceive for each item. A 5-point Likert scale is used for the evaluation. Scores from each item are aggregated, and a score for each domain is calculated. The score for each domain is represented on a scale between 0 and 1, with higher scores indicating better QOL.

3 Other Information

There is no fee for using AQoL-4D. Construct validity was an r-count of between 0.347 and 0.595, and reliability was Cronbach’s α value of 0.669 [2].

References

[1] Hawthorne G, Richardson J, Osborne R. The assessment of quality of life (AQoL) instrument: a psychometric measure of health-related quality of life. Quality of Life Research. 2006;8(3), 209-224.

[2] Achmad GNV, Husna RM, Priyandani Y, Zairina E. Translation, validation, and reliability of the Indonesian version. Assessment of Quality of Life-4 Dimensions (AQoL-4D). Pharmacy Education 2023;23(2):9-13.

15D instrument

1. Characteristics

The 15D instrument is a multi-dimensional generic tool that is used to assess a patient’s health status and QOL [1]. This scale evaluates information related to a patient’s health status across 15 dimensions: mobility, vision, hearing, breathing, sleep, eating, elimination, usual activities, mental function, distress, depressive symptoms, self-esteem, interpersonal relationships, sexual activity, and QOL evaluations, in the past, present, and future.

2. Procedure and Method

The evaluator asks a patient questions about their health and QOL in the past week. Questions relate to the 15 dimensions listed above.　Patients assess how much of a problem they perceive for each item. A 5-point Likert scale is used for the evaluation. Scores from each item are aggregated, and a score for each dimension is calculated. The score for each domain is represented on a scale from 0 to 1, with higher scores indicating better QOL.

3 Other Information

There is no fee for using the 15D instrument. It was previously shown to be highly sensitive for detecting changes in a patient’s health status, and its internal consistency and test-retest reliability have been confirmed [2-3]. The minimally important clinical difference for a change is ± 0.015 [3].

References

[1] Sintonen H. The 15D-measure of health-related quality of life: Reliability, validity and sensitivity of its health state descriptive system. National Centre for Health Program Evaluation. Working Paper 41, Melbourne; 1994.

[2] Sintonen H. The 15D instrument of health-related quality of life: properties and applications. Ann Med. 2001;33(5):328-36.

[3] Alanne, S., Roine, R. P., Räsänen, P., Vainiola, T., Sintonen, H. Estimating the minimum important change in the 15D scores. Quality of Life Research. 2015;24(3),599-606.

Pittsburgh Sleep Quality Index (PSQI)

1. Characteristics

The PSQI is a self-reported questionnaire developed to assess the quality of sleep in adults [1]. It consists of 7 components: sleep quality, sleep duration, sleep efficiency, sleep disturbances, use of sleep medication, daytime dysfunction, and overall sleep quality. Each component is scored on a scale of 0-3, and scores are then summed. A higher score indicates poorer sleep quality.

2. Procedure and Method

Subjects are asked about their sleep in the past month. Questions cover subjective sleep quality, sleep duration, sleep efficiency, sleep disturbances, use of sleep medication, daytime dysfunction, and overall sleep quality. Based on subjects’ answers, each component is scored on a scale of 0 to 3. The scores of the seven components are summed to calculate the overall score. An overall score of 5 or higher suggests a lower quality of sleep.

3 Other Information

There is no fee for using the PSQI. Its reliability and internal consistency were previously shown to be high. Consistent findings between initial evaluations and re-evaluations were confirmed, showing a strong correlation [2-3]. Test-retest reliability was 0.86 (0.57–0.91) [4]. The correlation of PSQI and Insomnia Severity Index is r=0.797 [5]. The minimum clinically important difference was 4.4 [6].

References

[1] Buysse DJ, Reynolds III CF, Monk THJ. The Pittsburgh sleep quality index: A new instrument for psychiatric practice and research. Psychiatry Research.1989;28(2),193-213.

[2] Carpenter JS, Andrykowski MA. Psychometric evaluation of the Pittsburgh sleep quality index. Journal of Psychosomatic Research. 1998;45(1),5-13.

[3] Backhaus J, Junghanns K, Broocks A, Riemann D, Hohagen F. Test–retest reliability and validity of the Pittsburgh sleep quality index in primary insomnia. Journal of Psychosomatic Research. 2002;53(3),737-40.

[4] Rener-Sitar K, John MT, Bandyopadhyay D, Howell MJ, Schiffman EL. Exploration of dimensionality and psychometric properties of the Pittsburgh Sleep Quality Index in cases with temporomandibular disorders. Health Qual Life Outcomes 2014;12:10.

[5] Gomes A, Marques D, Meiavia A, Cunha F, Clemente V. Psychometric properties and accuracy of the European Portuguese version of the Pittsburgh Sleep Quality Index in clinical and non-clinical samples. Sleep and Biological Rhythms 2018;16.

[6] Longo UG, Berton A, De Salvatore S, Piergentili I, Casciani E, Faldetta A, De Marinis MG, Denaro V. Minimal clinically important difference and patient acceptable symptom state for the Pittsburgh Sleep Quality Index in patients who underwent rotator cuff tear repair. Int J Environ Res Public Health 2021;18(16).

Insomnia Severity Index (ISI)

1. Characteristics

The ISI is a self-reported questionnaire that was designed to assess the severity of insomnia [1]. It consists of 7 items that evaluate the impact of insomnia (satisfaction with sleep, difficulty falling asleep, difficulty staying asleep, and early morning awakenings), the degree of distress caused by sleep problems, the impact on daily functions, and concerns about insomnia. Each item is scored on a scale of 0-4, and scores are then summed. A higher score indicates more severe insomnia.

2. Procedure and Method

Subjects answer questions related to the 7 items. Each item is scored on a scale of 0 to 4, and scores are summed to calculate the total score. The overall score ranges between 0 and 28, with higher scores indicating more severe insomnia.

3 Other Information

There is no fee for using the ISI. The internal consistency, test-retest reliability, and validity of evaluating treatment responses by the ISI have been verified [2]. In research, the ISI has demonstrated high reliability and validity, confirming its suitability for assessing treatment effects.

References

[1] Bastien CH, Vallières A, Morin CM. Validation of the insomnia severity index as an outcome measure for insomnia research. Sleep Medicine. 2001;2(4),297-307.

[2] Morin CM, Belleville G, Bélanger L, Ivers H. The Insomnia severity index: psychometric indicators to detect insomnia cases and evaluate treatment response. Sleep. 2011;34(5),601-608.

Actigraphy

1. Characteristics

Actigraphy is a non-invasive method that is employed to assess patterns of sleep and activity using a device called an actigraph to record movement [1]. The actigraph is typically attached to a subject’s wrist or ankle and collects continuous motion data over a specific period (generally one to two weeks). Data are analyzed to help assess patterns of sleep and wakefulness, sleep quality, and daytime activity levels. Actigraphy is used under various clinical and research conditions. In comparisons with polysomnography, it is less invasive and more cost-effective, and is also useful for evaluating sleep disorders, detecting sleep-related diseases, and monitoring the effects of sleep treatments [2].

2. Procedure and Method

Subjects are fit with an actigraphy on their wrist or ankle. While the usual wear time is between one to two weeks, it may vary depending on the research or clinical purpose. The actigraphy continuously collects motion data, which is later analyzed using specialized software. Various algorithms are employed in the analysis, evaluating patterns of sleep/wake, sleep quality, and daytime activity levels. The data gathered are typically converted into indicators, such as sleep latency, total sleep time, number of awakenings, time spent awake, and sleep efficiency. These indicators are then used to evaluate a subject’s sleep patterns and activity levels.

3 Other Information

There is no fee for using actigraphy. It has reasonable validity and reliability in normal individuals with relatively good sleep patterns; however, its validity in populations with poor sleep or other sleep-related disorders is questionable [3].

References

[1] Ancoli-Israel, S., Cole, R., Alessi, C., Chambers, M. The role of actigraphy in the study of sleep and circadian rhythms. Sleep.2003;26(3), 342-392.

[2] Sadeh, A., Acebo, C. The role of actigraphy in sleep medicine. Sleep Medicine Reviews. 2002;6(2),113-124.

[3] Sadeh A. The role and validity of actigraphy in sleep medicine: an update. Sleep Med Rev 2011;15(4):259-67.

Brief Pain Inventory (BPI)

1. Characteristics

The BPI is a self-assessment questionnaire tool that was designed to evaluate chronic pain [1]. It was developed to assess the intensity of pain and its impact on daily life. The BPI consists of two main sections. It evaluates the severity of pain and then evaluates its impact on functionality. It is widely used internationally as a tool for evaluating chronic pain, and its reliability and validity have been recognized. A previous study demonstrated its applicability to outcome evaluations in patients with non-cancerous pain [2].

2. Procedure and Method

Patients are provided with the questionnaire and asked to self-assess the intensity of their pain and its impact on functionality. The intensity of pain is evaluated from perspectives such as the most painful moment, the least painful moment, and current pain. Patients evaluate the impact of pain on their daily life functions, including aspects such as general activities, work, sleep, mood, enjoyment, and relationships. Each item is rated on a scale of 0 (no impact) to 10 (complete impact). Evaluators then calculate scores for pain intensity and its impact on functionality based on the information provided by patients. These scores are useful for evaluating the effectiveness of treatments and planning pain management.

3 Other Information

There is no fee for using the BPI. Reliability was r = 0.8 for pain intensity and r = 0.8 for pain interference in patients with malignant pain [3]. Internal consistency was 0.81 < α < 0.89 for pain severity and 0.88 < α < 0.95 for pain interference [4]. The score showed a reduction of 1.9-4.0 points in patients with reduced pain [5].

References

[1] Cleeland CS, Ryan KM. Pain assessment: global use of the brief pain inventory. Ann Acad Med Singap. 1994;23(2):129-38.

[2] Keller S, Bann CM, Dodd SL, Schein J. Validity of the brief pain inventory for use in documenting the outcomes of patients with noncancer pain. Clin J Pain. 2004;20(5):309-18.

[3] Pelayo-Alvarez M, Perez-Hoyos S, Agra-Varela Y. Reliability and concurrent validity of the Palliative Outcome Scale, the Rotterdam Symptom Checklist, and the Brief Pain Inventory. J Palliat Med 2013;16(8):867-74.

[4] Poquet N, Lin C. The Brief Pain Inventory (BPI). J Physiother 2016;62(1):52.

[5] Wong K, Zeng L, Zhang L, Bedard G, Wong E, Tsao M, Barnes E, Danjoux C, Sahgal A, Holden L. Minimal clinically important differences in the brief pain inventory in patients with bone metastases. Support Care Cancer 2013;21(7):1893-9.

Numerical Rating Scale (NRS)

1. Characteristics

The NRS is a simple evaluation method that quantifies the intensity of pain or discomfort [1]. It is typically rated on a scale of 0 (no pain) to 10 (worst imaginable pain). Its simplicity and ease of understanding make it widely applicable across various age groups and settings, and it is extensively used in clinical settings.

2. Procedure and Method

The evaluator instructs a patient to rate the intensity of their current pain on a scale of 0 (no pain) to 10 (worst imaginable pain). The patient then assesses their pain based on this scale and reports it to the evaluator.

3 Other Information

There is no fee for using the NRS. It has higher reliability and validity than other pain assessment scales, and it is considered to be useful for evaluating pain in adults [2].

References

[1] Ferreira-Valente MA, Pais-Ribeiro JL, Jensen MP. Validity of four pain intensity rating scales. Pain. 2011;152(10),2399-2404.

[2] Hawker GA, Mian S, Kendzerska T, French M. Measures of adult pain: Visual analog scale for pain (VAS Pain), numeric rating scale for pain (NRS Pain), McGill pain questionnaire (MPQ), short-form McGill pain questionnaire (SF-MPQ), chronic pain grade scale (CPGS), short form-36 bodily pain scale (SF-36 BPS), and measure of intermittent and constant osteoarthritis pain (ICOAP). Arthritis Care & Research. 2011;63(S11), S240-S252.

VAS

1. Characteristics

The VAS is a visual scale that evaluates the intensity of pain or discomfort [1]. It is typically represented by a 10-cm straight line, with one end indicating “no pain” (0 points) and the other denoting “worst imaginable pain” (10 points). Patients place a mark on the line corresponding to their perceived level of pain. Beyond a pain evaluation, the VAS is also employed to assess other symptoms and conditions, such as chronic fatigue and depressive symptoms.

2. Procedure and Method

The evaluator instructs a patient to rate the intensity of their current pain on the straight line. The patient then places a mark on the line corresponding to their perceived pain intensity. The evaluator measures the distance from the mark to one end of the line, quantifying pain intensity on a scale of 0 (no pain) to 10 (worst imaginable pain).

3 Other Information

There is no fee for using the VAS. It is considered useful for assessing pain in adults and has been validated as a ratio scale for evaluating both chronic and experimental pain. Since the VAS is a subjective evaluation, consistency may vary between patients and evaluators. Therefore, the VAS is recommended for use in combination with other pain assessment scales, such as the NRS or MPQ, under some conditions [2].

References

[1] Hawker GA, Mian S, Kendzerska T, French M. Measures of adult pain: Visual analog scale for pain (VAS Pain), numeric rating scale for pain (NRS Pain), McGill pain questionnaire (MPQ), short-form McGill pain questionnaire (SF-MPQ), chronic pain grade scale (CPGS), short form-36 bodily pain scale (SF-36 BPS), and measure of intermittent and constant osteoarthritis pain (ICOAP). Arthritis Care & Research. 2011;63(S11), S240-S252.

[2] Price DD, McGrath PA, Rafii A, Buckingham B. The validation of visual analogue scales as ratio scale measures for chronic and experimental pain. Pain. 1983;17(1), 45-56.

Graded Chronic Pain Scale (GCPS)

1. Characteristics

The GCPS was developed to evaluate the severity of pain in patients with chronic pain [1]. It assesses the intensity of pain, its impact on daily life, and disability associated with pain. It classifies the degree of chronic pain into five grades from 0 (no pain) to IV (severe pain), assisting in patient pain management and treatment planning. The GCPS strongly correlates with other pain assessment scales when evaluating the severity of pain and the degree of associated disability. The classification of chronic pain using the GCPS also aids in predicting the effectiveness of pain treatments [2].

2. Procedure and Method

The evaluator asks the patient about the intensity of pain (on a scale of 0-10), the impact of pain on daily life, and disability associated with pain. Based on the patient’s responses, the evaluator classifies the severity of pain into five grades ranging from 0 (no pain) to IV (severe pain).

3 Other Information

There is no fee for using the GCPS. The reliability of the 1-month GCPS is equal to or better than the 6-month version for pain intensity, disability days, pain interference, chronic pain grade, and high-impact pain [3]. However, consistency is lower for measures of disability days and interference and for the derived measures of the chronic pain grade and high-impact pain.

References

[1] Von Korff M, Ormel J, Keefe FJ, Dworkin SF. Grading the severity of chronic pain. Pain. 1992;50(2),133-149.

[2] Smith BH, Penny KI, Purves AM, Munro C. The Chronic pain grade questionnaire: validation and reliability in postal research. Pain. 1997;71(2),141-147.

[3] Sharma S, Kallen MA, Ohrbach R. Graded Chronic Pain Scale: Validation of 1-month reference frame. Clin J Pain 2021;38(2):119-31.

Fatigue Severity Scale (FSS)

1. Characteristics

The FSS was developed to evaluate the severity of chronic fatigue symptoms [1]. It consists of 9 items that allow for a subjective assessment of the impact of fatigue. This scale is used to evaluate fatigue in various diseases and conditions and is widely utilized to assess treatment effects and in research. Its reliability and validity have been confirmed in patients with multiple sclerosis as well as in healthy controls. FSS items adequately assess the severity of fatigue and related factors. The FSS strongly correlates with other fatigue assessment scales, suggesting its appropriateness as a tool for evaluating fatigue [2].

2. Procedure and Method

The evaluator asks a patient questions related to the 9 items. The patient rates each item on a scale of 1 (strongly disagree) to 7 (strongly agree). The average score of all items is calculated to evaluate the severity of fatigue.

3 Other Information

There is no fee for using the FSS. It demonstrated high internal consistency (Cronbach’s α value of 0.96) [3] as well as moderate to strong correlations with patient-reported anchors (vitality question from SF-6D [r = 0.55] and University of California, San Diego, Shortness of Breath Questionnaire total score [r = 0.70]) and weak correlations with physiological measures (FVC [r = -0.24], percentage predicted DLCO [r = -0.23], and 6-minute-walk distance [r = -0.29]) in an interstitial lung disease cohort.

References

[1] Krupp LB, LaRocca NG, Muir-Nash J, Steinberg AD. The fatigue severity scale: application to patients with multiple sclerosis and systemic lupus erythematosus. Archives of Neurology. 1989;46(10), 1121-1123.

[2] Lerdal A, Wahl A, Rustoen T, Hanestad BR, Moum T. Fatigue in the general population: A translation and test of the psychometric properties of the Norwegian version of the fatigue severity scale. Scandinavian Journal of Public Health. 2005;33(2),123-130.

[3] Aronson KI, Martin-Schwarze AM, Swigris JJ, Kolenic G, Krishnan JK, Podolanczuk AJ, Kaner RJ, Martinez FJ, Safford MM, Pinheiro LC. Validity and reliability of the Fatigue Severity Scale in a real-world interstitial lung disease cohort. Am J Respir Crit Care Med 2023;208(2):188-95.

Fatigue Assessment Scale (FAS)

1. Characteristics

The FAS comprises 10 items, each of which is evaluated using a 5-point Likert scale (1 = does not apply at all, 5 = applies very much). Since the FAS assesses both physical and mental fatigue, it is applicable to a wide range of research and clinical settings. The FAS has high internal consistency (Cronbach’s α value of 0.90), and structural equation modeling showed that the FAS adequately assessed both physical and mental fatigue [1].

2. Procedure and Method

The following steps are performed to administer the FAS: the FAS questionnaire is distributed to participants, who rate the 10 items on a scale of 1 to 5. The scores for each item are summed to calculate the total score. The total score is used to evaluate the level of fatigue.

3 Other Information

There is no fee for using the FAS. Reliability between total scores was good (ICC = 0.73). FAS correlated with the SF-36 subscale for vitality (rs = -0.73) and GDS-15 (rs = 0.62).

References

[1] Michielsen HJ, De Vries J, Van Heck GL. Psychometric qualities of a brief self-rated fatigue measure: The fatigue assessment scale. Journal of Psychosomatic Research. 2003;54(4),345-352.

[2] Bråndal A, Eriksson M, Wester P, Lundin-Olsson L. Reliability and validity of the Swedish Fatigue Assessment Scale when self-administrered by persons with mild to moderate stroke. Top Stroke Rehabil 2016;23(2):90-7.

Measure of Current Status part A (MOCS-A)

1. Characteristics

The MOCS-A is a self-reported questionnaire that was developed to assess the psychosocial health status of cancer patients [1]. It consists of four subscales: psychological health, social health, mental health, and overall health. Each item is evaluated using a 5-point Likert scale.

2. Procedure and Method

The MOCS-A is administered to a patient who completes the questionnaire. The questionnaire contains items for patients to evaluate their current psychological, social, mental, and overall health status, with each item being assessed on a 5-point Likert scale. Healthcare professionals or researchers typically assist with its administration, and after the answers are completed, they calculate scores and analyze the findings.

3 Other Information

There is no fee for using the MOCS-A. It has been validated in medically-ill populations [2].

References

[1] Antoni MH, Lechner SC, Kazi A, Wimberly SR. How stress management improves quality of life after treatment for breast cancer. Journal of Consulting and Clinical Psychology. 2006;74,1143-52.

[2] Bannon S, Lester EG, Gates MV, McCurley J, Lin A, Rosand J, Vranceanu A-M. Recovering together: building resiliency in dyads of stroke patients and their caregivers at risk for chronic emotional distress; a feasibility study. Pilot and Feasibility Studies 2020;6(1):75.

General Self-Efficacy (GSE) Scale

1. Characteristics

The GSE was designed to assess an individual’s sense of self-efficacy, and its reliability and validity have been confirmed [1]. It is a 10-item Likert scale with responses ranging from 1 (no confidence at all) to 4 (very confident).

2. Procedure and Method

Participants are given the GSE scale and are asked to respond to the 10 items using a scale of 1 to 4. The scores for each item are then totaled to derive an aggregate score. The total score is used to assess the degree of self-efficacy.

3 Other Information

There is no fee for using the GSE. It may be applied across diverse cultures and age groups and allows for the measurement of self-efficacy that transcends specific conditions. It has been shown to correlate with stress resilience and coping skills and is widely recommended for measuring self-efficacy [2].

References

[1] Schwarzer R, Jerusalem M. Generalized Self-Efficacy scale. In J. Weinman, S. Wright, M. Johnston, Measures in health psychology: A user's portfolio. Causal and control beliefs (pp. 35-37). Windsor, UK: NFER-NELSON; 1995

[2] Leganger A, Kraft P, Røysamb E. Perceived self-efficacy in health behaviour research: Conceptualisation, measurement and correlates. Psychology & Health. 2000;15(1),51-69.

Family Satisfaction in the ICU (FS-ICU)

1. Characteristics

FS-ICU is a questionnaire that was designed to evaluate family satisfaction with patient care and communication/support provided to the family in the Intensive Care Unit (ICU) [1]. The original FS-ICU was developed by Heyland, and a shortened 24-item version (FS-ICU-24) was subsequently developed by Wall, enhancing its ease of use and practicality [2]. FS-ICU-24 consists of two subscales: satisfaction with care and satisfaction with decision-making/communication. It has demonstrated very high internal consistency, and factor analyses confirmed the construct validity of FS-ICU 24. Based on these findings, FS-ICU 24 is recommended as a reliable and valid method for assessing family satisfaction in the ICU.

2. Procedure and Method

To administer FS-ICU 24, participants (family members of the patient) are given the FS-ICU-24 questionnaire and asked to respond to the 24 items using a 5-point scale (1 = very dissatisfied, 5 = very satisfied). Scores for each item are totaled to derive an aggregate score for the two subscales: satisfaction with care and satisfaction with decision-making/communication. The total score is used to evaluate the satisfaction of the family in the ICU.

3 Other Information

There is no fee for using FS-ICU. Internal consistency was high (Cronbach’s α value of 0.96; satisfaction with care, 0.94; satisfaction with decision-making, 0.93) [3].

References

[1] Heyland DK, Rocker GM, Dodek PM, Kutsogiannis DJ. Family satisfaction with care in the intensive care unit: Results of a multiple center study. Critical Care Medicine. 2002;30(7), 1413-8.

[2] Wall RJ, Engelberg RA, Downey L, Heyland DK, Curtis JR. Refinement, scoring, and validation of the family satisfaction in the intensive care unit (FS-ICU) survey. Critical Care Medicine. 2007;35(1),271-9.

[3] Harrison DA, Ferrando-Vivas P, Wright SE, McColl E, Heyland DK, Rowan KM. Psychometric assessment of the Family Satisfaction in the Intensive Care Unit questionnaire in the United Kingdom. J Crit Care 2017;38:346-50.

Quality of Death and Dying (QODD)

1. Characteristics

QODD is a scale that was designed to evaluate the quality of death and the dying process. It was developed by Curtis et al. in 2000. This scale is used to assess the quality of palliative care and the response of healthcare professionals to the needs of patients and their families [1]. QODD is used to assess the quality of death in various settings, such as hospitals and hospices. It consists of 10 items rated on a 10-point Likert scale.

2. Procedure and Method

Participants (the family members of a patient or care staff) are given the QODD questionnaire and asked to rate the 10 items on a scale of 1 to 10 (1=worst, 10=best). The scores for each item are then summed to derive a total score. The total score is used to assess the participant’s evaluation of the quality of death and the dying process.

3 Other Information

There is no fee for using QODD. Its internal consistency was high (Cronbach’s α value of 0.81) and construct validity was confirmed. Therefore, QODD is recommended as a reliable and valid measure for assessing the quality of death and the dying process [2].

References

[1] Curtis JR, Patrick DL, Engelberg RA, Norris K. A measure of the quality of dying and death: initial validation using after-death interviews with family members. Journal of Pain and Symptom Management. 2000;20(1),17-31.

[2] Downey L, Curtis JR, Lafferty WE, Herting JR. The quality of dying and death questionnaire (QODD): Empirical domains and theoretical perspectives. Journal of Pain and Symptom Management. 2009;39(1),9-22.

Zarit Burden Interview 12 items (Zarit-12)

1. Characteristics

While the original measure consisted of 22 items, the shortened version, Zarit-12, comprises 12 items and assesses the psychological, social, and temporal burdens of caregivers [1]. This scale is used for family caregivers as well as non-family caregivers and is widely employed to measure the sense of burden among caregivers in various diseases and conditions. Due to its strong correlation with the original 22-item version, Zarit-12 has been suggested to provide similar information to the original scale.

2. Procedure and Method

Participants (caregivers) are provided with the Zarit-12 questionnaire and asked to rate 12 items on a 5-point scale (1=do not feel at all, 5=feel very strongly). Scores for each item are then summed to derive the total score. The total score is used to assess the sense of burden of the caregiver.

3 Other Information

There is no fee for using Zarit-12. The construct validity of Zarit-12 and its validity with other caregiver stress scales have been confirmed [2].

References

[1] Zarit SH, Reever KE, Bach-Peterson J. Relatives of the impaired elderly: correlates of feelings of burden. Gerontologist. 1980;20(6):649-55.

[2] Bédard M, Molloy DW, Squire L, Dubois S. The Zarit burden interview: A new short version and screening version. The Gerontologist. 2001;41(5),652-657.

**3. The study included in scoping review**

| Year | Journal | Title |
| --- | --- | --- |
| 2014 | Am J Respir Crit Care Med | Risk factors for physical impairment after acute lung injury in a national, multicenter study |
| 2014 | Am J Respir Crit Care Med | Smoking cessation after surviving critical illness-is it feasible? Authors experience with implementation and recruitment |
| 2014 | Ann Am Thorac Soc | Development and preliminary evaluation of a telephone-based mindfulness training intervention for survivors of critical illness |
| 2014 | Archives of Physical Medicine & Rehabilitation | Hospital Delirium and Psychological Distress at 1 Year and Health-Related Quality of Life After Moderate-to-Severe Traumatic Injury Without Intracranial Hemorrhage |
| 2014 | Aust Crit Care | Posttraumatic stress disorder in close Relatives of Intensive Care unit patients' Evaluation (PRICE) study |
| 2014 | British journal of anaesthesia | Effect of supervised aerobic exercise rehabilitation on physical fitness and quality-of-life in survivors of critical illness: an exploratory minimized controlled trial (PIX study) |
| 2014 | Cmaj | Association between frailty and short- and long-term outcomes among critically ill patients: a multicentre prospective cohort study |
| 2014 | Crit Care | Early prediction of new-onset physical disability after intensive care unit stay: a preliminary instrument |
| 2014 | Crit Care | Long-term outcome of delirium during intensive care unit stay in survivors of critical illness: a prospective cohort study |
| 2014 | Crit Care Med | Psychiatric symptoms and acute care service utilization over the course of the year following medical-surgical ICU admission: a longitudinal investigation* |
| 2014 | Crit Care Med | Short- and long-term outcome in elderly patients after out-of-hospital cardiac arrest: a cohort study |
| 2014 | Crit Care Med | Awakening and withdrawal of life-sustaining treatment in cardiac arrest survivors treated with therapeutic hypothermia* |
| 2014 | Crit Care Med | Delirium in the ICU and subsequent long-term disability among survivors of mechanical ventilation |
| 2014 | Crit Care Med | Physical complications in acute lung injury survivors: a two-year longitudinal prospective study |
| 2014 | European archives of oto-rhino-laryngology | Acute and long-term dysphagia in critically ill patients with severe sepsis: results of a prospective controlled observational study |
| 2014 | Health Psychol Behav Med | Posttraumatic stress in intensive care unit survivors - a prospective study |
| 2014 | Int Nurs Rev | Jordanian survivors' experiences of recovery from critical illness: a qualitative study |
| 2014 | Intensive Care Med | Outcome measures report different aspects of patient function three months following critical care |
| 2014 | Intensive Crit Care Nurs | Psychological wellbeing, health related quality of life and memories of intensive care and a specialised weaning centre reported by survivors of prolonged mechanical ventilation |
| 2014 | Issues Ment Health Nurs | The prevalence of major depression-PTSD comorbidity among ICU survivors in five general hospitals of Athens: a cross-sectional study |
| 2014 | J Crit Care | The feasibility of measuring frailty to predict disability and mortality in older medical intensive care unit survivors |
| 2014 | J Crit Care | A pilot investigation of the association of genetic polymorphisms regulating corticotrophin-releasing hormone with posttraumatic stress and depressive symptoms in medical-surgical intensive care unit survivors |
| 2014 | J Crit Care | Critically ill elderly patients in a developing world--mortality and functional outcome at 1 year: a prospective single-center study |
| 2014 | J Crit Care | Risk factors for prolonged duration of mechanical ventilation in acute traumatic tetraplegic patients--a retrospective cohort study |
| 2014 | J Pain Symptom Manage | Self-reported physical symptoms in intensive care unit (ICU) survivors: pilot exploration over four months post-ICU discharge |
| 2014 | J Pain Symptom Manage | Fatigue in family caregivers of adult intensive care unit survivors |
| 2014 | J Peripher Nerv Syst | Post-traumatic stress symptoms in Guillain-Barré syndrome patients after prolonged mechanical ventilation in ICU: a preliminary report |
| 2014 | J Surg Res | Moving beyond survival as a measure of success: understanding the patient experience of necrotizing soft-tissue infections |
| 2014 | J Trauma Acute Care Surg | Persistent inflammation-immunosuppression catabolism syndrome, a common manifestation of patients with enterocutaneous fistula in intensive care unit |
| 2014 | Kardiol Pol | Neuropsychological and neurological sequelae of out-of-hospital cardiac arrest and the estimated need for neurorehabilitation: a prospective pilot study |
| 2014 | Lancet Respir Med | Depression, post-traumatic stress disorder, and functional disability in survivors of critical illness in the BRAIN-ICU study: a longitudinal cohort study |
| 2014 | Nursing in critical care | A post cardiac surgery intervention to manage delirium involving families: a randomized pilot study |
| 2014 | Physical therapy | The physical function intensive care test: implementation in survivors of critical illness |
| 2014 | PLoS Med | Long-term survival and dialysis dependency following acute kidney injury in intensive care: extended follow-up of a randomized controlled trial |
| 2014 | PLoS One | Long term health-related quality of life in survivors of sepsis in South West Wales: an epidemiological study |
| 2014 | Qjm | Psychological burden in inpatient relatives: the forgotten side of medical management |
| 2014 | Resuscitation | Cardiac arrest and hypothermia treatment--function and life satisfaction among survivors in the first 6 months |
| 2014 | Resuscitation | Prevalence and risk factors for post-traumatic stress disorder in relatives of out-of-hospital cardiac arrest patients |
| 2014 | Scand J Trauma Resusc Emerg Med | Cardiac arrest survivors treated with or without mild therapeutic hypothermia: performance status and quality of life assessment |
| 2014 | West Indian Med J | Outcomes and Health-related Quality of Life following Intensive Care Unit Stay in Barbados |
| 2015 | Am J Crit Care | Physical recovery in intensive care unit survivors: a cohort analysis |
| 2015 | Anaesth Crit Care Pain Med | Decompression surgery for severe traumatic brain injury (TBI): A long-term, single-centre experience |
| 2015 | Anaesth Intensive Care | Functional and quality-of-life outcomes in older survivors of an Australian intensive care unit |
| 2015 | Ann Am Thorac Soc | Volume Overload: Prevalence, Risk Factors, and Functional Outcome in Survivors of Septic Shock |
| 2015 | Ann Am Thorac Soc | Health-Related Quality of Life in Australasian Survivors of H1N1 Influenza Undergoing Mechanical Ventilation. A Multicenter Cohort Study |
| 2015 | Ann Intensive Care | Long-term survival and quality of life after intensive care for patients 80 years of age or older |
| 2015 | Annals of the American Thoracic Society | The influence of hospitalization or intensive care unit admission on declines in health-related quality of life |
| 2015 | Annals of Thoracic Medicine | Original Article. Determinants of functional status among survivors of severe sepsis and septic shock: One-year follow-up |
| 2015 | Australian Critical Care | The nature of death, coping response and intensity of bereavement following death in the critical care environment |
| 2015 | BMC Anesthesiol | Long-term treated intensive care patients outcomes: the one-year mortality rate, quality of life, health care use and long-term complications as reported by general practitioners |
| 2015 | BMJ open | Evaluating the feasibility and effectiveness of a critical care discharge information pack for patients and their families: a pilot cluster randomised controlled trial |
| 2015 | BMJ open | Costs and quality of life associated with acute upper gastrointestinal bleeding in the UK: cohort analysis of patients in a cluster randomised trial |
| 2015 | Burns | The responsiveness of the Chelsea Critical Care Physical Assessment tool in measuring functional recovery in the burns critical care population: an observational study |
| 2015 | Crit Care | Long-term quality of life in critically ill patients with acute kidney injury treated with renal replacement therapy: a matched cohort study |
| 2015 | Crit Care | Cognitive screening among acute respiratory failure survivors: a cross-sectional evaluation of the Mini-Mental State Examination |
| 2015 | Crit Care | Impact of ICU-acquired weakness on post-ICU physical functioning: a follow-up study |
| 2015 | Crit Care Med | Long-term association between frailty and health-related quality of life among survivors of critical illness: a prospective multicenter cohort study |
| 2015 | Crit Care Med | Cooccurrence of and remission from general anxiety, depression, and posttraumatic stress disorder symptoms after acute lung injury: a 2-year longitudinal study |
| 2015 | Crit Care Med | Dichotomous "Good Outcome" Indicates Mobility More Than Cognitive or Social Quality of Life |
| 2015 | Crit Care Med | Recall of ICU Stay in Patients Managed With a Sedation Protocol or a Sedation Protocol With Daily Interruption |
| 2015 | Crit Care Med | Coping Strategies and Posttraumatic Stress Symptoms in Post-ICU Family Decision Makers |
| 2015 | Crit Care Med | Stress disorders following prolonged critical illness in survivors of severe sepsis |
| 2015 | Dan Med J | Experience from multidisciplinary follow-up on critically ill patients treated in an intensive care unit |
| 2015 | Disabil Rehabil | Joint contractures in the intensive care unit: quality of life and function 3.3 years after hospital discharge |
| 2015 | Heart Lung | Insomnia is associated with quality of life impairment in medical-surgical intensive care unit survivors |
| 2015 | Intensive Care Med | ICU survivors show no decline in health-related quality of life after 5 years |
| 2015 | Intensive Care Med | Physical activity, muscle strength, and exercise capacity 3 months after severe sepsis and septic shock |
| 2015 | Intensive Care Med | Recovery after critical illness in patients aged 80 years or older: a multi-center prospective observational cohort study |
| 2015 | Intensive Care Med | Early physical rehabilitation in intensive care patients with sepsis syndromes: a pilot randomised controlled trial |
| 2015 | Intensive Crit Care Nurs | Severity of delirium in the ICU is associated with short term cognitive impairment. A prospective cohort study |
| 2015 | Intensive Crit Care Nurs | The influence of social support on patients' quality of life after an intensive care unit discharge: A cross-sectional survey |
| 2015 | J Crit Care | Posttraumatic stress disorder symptoms in close relatives of intensive care unit patients: Prevalence data resemble that of earthquake survivors in Chile |
| 2015 | J Crit Care | Psychological experience of patients 3 months after a stay in the intensive care unit: A descriptive and qualitative study |
| 2015 | J Crit Care | Symptom burden and health-related quality of life among intensive care unit survivors in Argentina: A prospective cohort study |
| 2015 | J Crit Care | Functional brain imaging in survivors of critical illness: A prospective feasibility study and exploration of the association between delirium and brain activation patterns |
| 2015 | J Crit Care | Psychometric evaluation of the Hospital Anxiety and Depression Scale 3 months after acute lung injury |
| 2015 | J Crit Care | Basal functional status predicts functional recovery in critically ill patients with multiple-organ failure |
| 2015 | J Crit Care | Single-center large-cohort study into quality of life in Dutch intensive care unit subgroups, 1 year after admission, using EuroQoL EQ-6D-3L |
| 2015 | J Crit Care | Exercise-based rehabilitation after hospital discharge for survivors of critical illness with intensive care unit-acquired weakness: a pilot feasibility trial |
| 2015 | J Intensive Care Soc | Depression following critical illness: Analysis of incidence and risk factors |
| 2015 | J Intensive Care Soc | Employment, social dependency and return to work after intensive care |
| 2015 | J Neurosci Res | White matter microstructure in chronic moderate-to-severe traumatic brain injury: Impact of acute-phase injury-related variables and associations with outcome measures |
| 2015 | J Trauma Nurs | Posttraumatic Stress Disorder After Discharge From Intensive Care Units in Greater Athens Area |
| 2015 | JAMA neurology | Neurologic Function and Health-Related Quality of Life in Patients Following Targeted Temperature Management at 33ﾃ・ﾂｰC vs 36ﾃ・ﾂｰC After Out-of-Hospital Cardiac Arrest: a Randomized Clinical Trial |
| 2015 | Minerva Anestesiol | Feasibility of Post-Intensive Care Unit Clinics: an observational cohort study of two different approaches |
| 2015 | Minerva Anestesiol | Functional status and medium-term prognosis of very elderly patients after an ICU stay: a prospective observational study |
| 2015 | Muscle Nerve | Small-nerve-fiber pathology in critical illness documented by serial skin biopsies |
| 2015 | Palliat Med | End-of-life care in the intensive care unit: a patient-based questionnaire of intensive care unit staff perception and relatives' psychological response |
| 2015 | Patient Prefer Adherence | Needs of family caregivers of stroke patients: a longitudinal study of caregivers' perspectives |
| 2015 | PLoS One | Emotional disorders in pairs of patients and their family members during and after ICU stay |
| 2015 | Resuscitation | Cognitive function and health-related quality of life four years after cardiac arrest |
| 2015 | Resuscitation | Greater temperature variability is not associated with a worse neurological outcome after cardiac arrest |
| 2015 | Resuscitation | Neurocognitive outcomes following successful resuscitation from cardiac arrest |
| 2015 | Shock | Long-term outcomes after severe shock |
| 2016 | Acta Anaesthesiol Scand | Perceived cognitive impairments after critical illness: a longitudinal study in survivors and family member controls |
| 2016 | Am J Crit Care | Health-Related Quality of Life and Associated Factors in Intensive Care Unit Survivors 6 Months After Discharge |
| 2016 | Am J Respir Crit Care Med | A Randomized Trial of an Intensive Physical Therapy Program for Patients with Acute Respiratory Failure |
| 2016 | Am J Respir Crit Care Med | Incidence and Risk Factors for Intensive Care Unit-related Post-traumatic Stress Disorder in Veterans and Civilians |
| 2016 | Am J Respir Crit Care Med | Randomized Trial of Communication Facilitators to Reduce Family Distress and Intensity of End-of-Life Care |
| 2016 | American journal of physical medicine & rehabilitation | Does 6-Month Home Caregiver-Supervised Physiotherapy Improve Post-Critical Care Outcomes?: a Randomized Controlled Trial |
| 2016 | Anaesth Intensive Care | A feasibility study of functional status and follow-up clinic preferences of patients at high risk of post intensive care syndrome |
| 2016 | Anaesth Intensive Care | The effect of augmenting early nutritional energy delivery on quality of life and employment status one year after ICU admission |
| 2016 | Ann Am Thorac Soc | A Novel Picture Guide to Improve Spiritual Care and Reduce Anxiety in Mechanically Ventilated Adults in the Intensive Care Unit |
| 2016 | Ann Am Thorac Soc | Resilience in Survivors of Critical Illness in the Context of the Survivors' Experience and Recovery |
| 2016 | Ann Intensive Care | Assessment and predictors of physical functioning post-hospital discharge in survivors of critical illness |
| 2016 | Ann Intensive Care | Predictors of posttraumatic stress and quality of life in family members of chronically critically ill patients after intensive care |
| 2016 | Aust Crit Care | In ICU state anxiety is not associated with posttraumatic stress symptoms over six months after ICU discharge: A prospective study |
| 2016 | Aust Crit Care | Challenges during long-term follow-up of ICU patients with and without chronic disease |
| 2016 | Biomed Res Int | Public versus Private Healthcare Systems following Discharge from the ICU: A Propensity Score-Matched Comparison of Outcomes |
| 2016 | BMC Pulm Med | Long-term outcome and health-related quality of life in difficult-to-wean patients with and without ventilator dependency at ICU discharge: a retrospective cohort study |
| 2016 | Br J Neurosurg | An evaluation of the clinical and cost-effectiveness of alternative care locations for critically ill adult patients with acute traumatic brain injury |
| 2016 | Brain Injury | Disability and health-related quality-of-life 4 years after a severe traumatic brain injury: A structural equation modelling analysis |
| 2016 | Clin Neurophysiol | Slowed peak resting frequency and MEG overactivation in survivors of severe sepsis and septic shock |
| 2016 | Crit Care | The predictive value of early acute kidney injury for long-term survival and quality of life of critically ill patients |
| 2016 | Crit Care Med | Prevalence and Characteristics of Chronic Intensive Care-Related Pain: The Role of Severe Sepsis and Septic Shock |
| 2016 | Crit Care Med | Trait Anxiety But Not State Anxiety During Critical Illness Was Associated With Anxiety and Depression Over 6 Months After ICU |
| 2016 | Crit Care Med | Psychiatric Symptoms in Acute Respiratory Distress Syndrome Survivors: A 1-Year National Multicenter Study |
| 2016 | Crit Care Med | An Exploratory Study of Long-Term Outcome Measures in Critical Illness Survivors: Construct Validity of Physical Activity, Frailty, and Health-Related Quality of Life Measures |
| 2016 | Crit Care Med | Coping as a Multifaceted Construct: Associations With Psychological Outcomes Among Family Members of Mechanical Ventilation Survivors |
| 2016 | Crit Care Med | Employment Outcomes After Critical Illness: An Analysis of the Bringing to Light the Risk Factors and Incidence of Neuropsychological Dysfunction in ICU Survivors Cohort |
| 2016 | Crit Care Med | Functional Status in ICU Survivors and Out of Hospital Outcomes: A Cohort Study |
| 2016 | Crit Care Med | Impact of Proactive Nurse Participation in ICU Family Conferences: a Mixed-Method Study |
| 2016 | Crit Care Med | Long-Term Mental Health Problems After Delirium in the ICU |
| 2016 | Crit Care Med | Long-Term Quality of Life Among Survivors of Severe Sepsis: analyses of Two International Trials |
| 2016 | Crit Care Resusc | Long-term outcomes after severe drug overdose |
| 2016 | Growth hormone & IGF research | Effects of sustained release growth hormone treatment during the rehabilitation of adult severe burn survivors |
| 2016 | Health Qual Life Outcomes | Fatigue in intensive care survivors one year after discharge |
| 2016 | Heart Lung | Depressive symptoms and anxiety in intensive care unit (ICU) survivors after ICU discharge |
| 2016 | Injury | Return to work after specialised burn care: A two-year prospective follow-up study of the prevalence, predictors and related costs |
| 2016 | Intensive & Critical Care Nursing | Sleep in family caregivers of ICU survivors for two months post-ICU discharge |
| 2016 | Intensive Care Med | The ENCOURAGE mortality risk score and analysis of long-term outcomes after VA-ECMO for acute myocardial infarction with cardiogenic shock |
| 2016 | Intensive Care Med | Physical declines occurring after hospital discharge in ARDS survivors: a 5-year longitudinal study |
| 2016 | Intensive Care Med | Prospective study of a proactive palliative care rounding intervention in a medical ICU |
| 2016 | Intensive Care Med | A recovery program to improve quality of life, sense of coherence and psychological health in ICU survivors: a multicenter randomized controlled trial, the RAPIT study |
| 2016 | Intensive Care Med | CAESAR: a new tool to assess relatives' experience of dying and death in the ICU |
| 2016 | J Am Geriatr Soc | Functional Status and Quality of Life in Elderly Intensive Care Unit Survivors |
| 2016 | J Intensive Care | Posttraumatic stress symptoms in families of cancer patients admitted to the intensive care unit: a longitudinal study |
| 2016 | J Intensive Care | One-year resource utilisation, costs and quality of life in patients with acute respiratory distress syndrome (ARDS): secondary analysis of a randomised controlled trial |
| 2016 | J Palliat Med | ICU versus Non-ICU Hospital Death: Family Member Complicated Grief, Posttraumatic Stress, and Depressive Symptoms |
| 2016 | J Phys Ther Sci | Quality of life of critically ill patients in a developing country: a prospective longitudinal study |
| 2016 | JAMA | Effect of Palliative Care-Led Meetings for Families of Patients With Chronic Critical Illness: a Randomized Clinical Trial |
| 2016 | JAMA | Effect of a Primary Care Management Intervention on Mental Health-Related Quality of Life Among Survivors of Sepsis: a Randomized Clinical Trial |
| 2016 | Journal of Advanced Nursing (John Wiley & Sons, Inc.) | Health status and quality of life of survivors of extra corporeal membrane oxygenation: a cross-sectional study |
| 2016 | Lancet respiratory medicine | Rosuvastatin versus placebo for delirium in intensive care and subsequent cognitive impairment in patients with sepsis-associated acute respiratory distress syndrome: an ancillary study to a randomised controlled trial |
| 2016 | Med Intensiva | Mortality and functional status at one-year of follow-up in elderly patients with prolonged ICU stay |
| 2016 | Minerva Anestesiol | Defining needs and goals of post-ICU care for trauma patients: preliminary study |
| 2016 | New England Journal of Medicine | One-Year Outcomes in Caregivers of Critically Ill Patients |
| 2016 | Open Orthop J | Open Pelvic Fractures: Review of 30 Cases |
| 2016 | Patient Relat Outcome Meas | The role of memories on health-related quality of life after intensive care unit care: an unforgettable controversy? |
| 2016 | Rehabil Psychol | Caregiver strain and posttraumatic stress symptoms of informal caregivers of intensive care unit survivors |
| 2016 | Rehabil Psychol | The intensive care unit experience: Psychological impact on family members of patients with and without traumatic brain injury |
| 2016 | Rehabilitation psychology | Coping style and quality of life in Dutch intensive care unit survivors |
| 2016 | Respir Care | Impact of Clinical and Quality of Life Outcomes of Long-Stay ICU Survivors Recovering From Rehabilitation on Caregivers' Burden |
| 2016 | Respir Med | Assessment of sleep quality post-hospital discharge in survivors of critical illness |
| 2016 | Turk J Anaesthesiol Reanim | Predictive Value of Brain Arrest Neurological Outcome Scale (BrANOS) on Mortality and Morbidity After Cardiac Arrest |
| 2016 | World Neurosurg | Outcome and Refractory Factor of Intensive Treatment for Geriatric Traumatic Brain Injury: Analysis of 1165 Cases Registered in the Japan Neurotrauma Data Bank |
| 2017 | Acta Clin Belg | Cerebral perfusion alterations and cognitive decline in critically ill sepsis survivors |
| 2017 | Am J Crit Care | Patients' Outcomes After Acute Respiratory Failure: A Qualitative Study With the PROMIS Framework |
| 2017 | Am J Phys Med Rehabil | Functional Recovery in Patients With and Without Intensive Care Unit-Acquired Weakness |
| 2017 | Am J Respir Crit Care Med | One-year outcomes in patients with acute respiratory distress syndrome enrolled in a trial of helmet versus facemask noninvasive ventilation |
| 2017 | Ann Am Thorac Soc | Six-Month Morbidity and Mortality among Intensive Care Unit Patients Receiving Life-Sustaining Therapy. A Prospective Cohort Study |
| 2017 | Ann Am Thorac Soc | Assessing the Usefulness and Validity of Frailty Markers in Critically Ill Adults |
| 2017 | Ann Intensive Care | Admission of tetanus patients to the ICU: a retrospective multicentre study |
| 2017 | Ann Surg | Surgery and Anesthesia Exposure Is Not a Risk Factor for Cognitive Impairment After Major Noncardiac Surgery and Critical Illness |
| 2017 | Annals of the American Thoracic Society | The Experience of Patients with Alcohol Misuse after Surviving a Critical Illness. A Qualitative Study |
| 2017 | Biomed Res Int | Self-Rated Health as a Predictor of Death after Two Years: The Importance of Physical and Mental Wellbeing Postintensive Care |
| 2017 | Chin Med J (Engl) | Assessment of 1-year Outcomes in Survivors of Severe Acute Respiratory Distress Syndrome Receiving Extracorporeal Membrane Oxygenation or Mechanical Ventilation: A Prospective Observational Study |
| 2017 | Clin Nurs Res | Development and Effects of a Transition Nursing Program for Patients and Family Caregivers at a Neurological ICU in Korea |
| 2017 | Clinics (Sao Paulo) | Characteristics and Outcomes of Intensive Care Unit Survivors: Experience of a Multidisciplinary Outpatient Clinic in a Teaching Hospital |
| 2017 | Crit Care | Early psychological screening of intensive care unit survivors: a prospective cohort study |
| 2017 | Crit Care Med | Somatosensory Functions in Survivors of Critical Illness |
| 2017 | Crit Care Med | Healthcare Resource Use and Costs in Long-Term Survivors of Acute Respiratory Distress Syndrome: A 5-Year Longitudinal Cohort Study |
| 2017 | Crit Care Res Pract | A Retrospective, Pilot Study of De Novo Antidepressant Medication Initiation in Intensive Care Unit Patients and Post-ICU Depression |
| 2017 | Eur J Phys Rehabil Med | The potential role of pain-related SSEPs in the early prognostication of long-term functional outcome in post-anoxic coma |
| 2017 | Gen Hosp Psychiatry | Acute psychological trauma in the critically ill: Patient and family perspectives |
| 2017 | Geriatr Nurs | Changes of geriatric syndromes in older adults survived from Intensive Care Unit |
| 2017 | Indian journal of critical care medicine | Quality of life improves with individualized home-based exercises in critical care survivors |
| 2017 | Injury | Psychological distress and physical disability in patients sustaining severe injuries in road traffic crashes: Results from a one-year cohort study from three European countries |
| 2017 | Intensive Care Med | Effect of a condolence letter on grief symptoms among relatives of patients who died in the ICU: a randomized clinical trial |
| 2017 | Intensive Care Med | Healthcare utilization and costs in ARDS survivors: a 1-year longitudinal national US multicenter study |
| 2017 | Intensive Care Med | The impact of disability in survivors of critical illness |
| 2017 | Intensive Care Med | Terminal weaning or immediate extubation for withdrawing mechanical ventilation in critically ill patients (the ARREVE observational study) |
| 2017 | Intensive Care Med Exp | Factors associated with non-response at quality of life follow-up among survivors of septic shock. A registry-based post-hoc analysis of the TRISS randomised trial |
| 2017 | Intensive Crit Care Nurs | Impact of prior ICU experience on ICU patient family members' psychological distress: A descriptive study |
| 2017 | Intensive Crit Care Nurs | Evaluating the past to improve the future - A qualitative study of ICU patients' experiences |
| 2017 | J Am Geriatr Soc | The Frailty Phenotype and Palliative Care Needs of Older Survivors of Critical Illness |
| 2017 | J Am Geriatr Soc | Long-Term Self-Reported Cognitive Problems After Delirium in the Intensive Care Unit and the Effect of Systemic Inflammation |
| 2017 | J Crit Care | Perspectives of patients and family members regarding psychological support using intensive care diaries: An exploratory mixed methods study |
| 2017 | J Crit Care | Derivation and validation of the automated search algorithms to identify cognitive impairment and dementia in electronic health records |
| 2017 | J Crit Care | The relationship between executive dysfunction, depression, and mental health-related quality of life in survivors of critical illness: Results from the BRAIN-ICU investigation |
| 2017 | J Crit Care | Relatives' perception of stressors and psychological outcomes - Results from a survey study |
| 2017 | J Crit Care | Cost-effectiveness of hospital treatment and outcomes of acute methanol poisoning during the Czech Republic mass poisoning outbreak |
| 2017 | J Crit Care | The long-term quality of life in patients with persistent inflammation-immunosuppression and catabolism syndrome after severe acute pancreatitis: A retrospective cohort study |
| 2017 | J Crit Care | Effect of neuromuscular stimulation and individualized rehabilitation on muscle strength in Intensive Care Unit survivors: a randomized trial |
| 2017 | J Hosp Med | Antidepressant Use and Depressive Symptoms in Intensive Care Unit Survivors |
| 2017 | J Hosp Med | Post-Intensive Care Unit Psychiatric Comorbidity and Quality of Life |
| 2017 | J Neurosurg Anesthesiol | Acute Kidney Injury After Subarachnoid Hemorrhage |
| 2017 | JAMA Neurol | Neurologic Complications Associated With the Zika Virus in Brazilian Adults |
| 2017 | Journal of clinical nursing | Intensive care unit survivorship' - a constructivist grounded theory of surviving critical illness |
| 2017 | Med Intensiva | The impact of the patient post-intensive care syndrome components upon caregiver burden |
| 2017 | Medicine (Baltimore) | Outcome and risk factors associated with extent of central nervous system injury due to exertional heat stroke |
| 2017 | Minerva Anestesiol | Critically ill octogenarians and nonagenarians: evaluation of long-term outcomes, posthospital trajectories and quality of life one year and seven years after ICU discharge |
| 2017 | Neurocritical care | Palliative Care Needs Assessment in the Neuro-ICU: effect on Family |
| 2017 | Neurocritical Care | Post-traumatic Stress Disorder and Complicated Grief are Common in Caregivers of Neuro-ICU Patients |
| 2017 | Neuropsychological rehabilitation | The effects of cognitive intervention on cognitive impairments after intensive care unit admission |
| 2017 | PLoS One | Intensive Care Syndrome: Promoting Independence and Return to Employment (InS:PIRE). Early evaluation of a complex intervention |
| 2017 | Prostaglandins, leukotrienes, and essential fatty acids | Limited effect of omega-3 fatty acids on the quality of life in survivors of traumatic injury: a randomized, placebo-controlled trial |
| 2017 | Psychiatry | Technology Use, Preferences, and Capacity in Injured Patients at Risk for Posttraumatic Stress Disorder |
| 2017 | Rehabilitation Oncology | A Descriptive Report of Early Mobilization for Critically Ill Ventilated Patients With Cancer |
| 2017 | Resuscitation | Post-admission outcomes of participants in the PARAMEDIC trial: a cluster randomised trial of mechanical or manual chest compressions |
| 2017 | Rural Remote Health | Long-term morbidity and mortality in survivors of critical illness: a 5-year observational follow-up study |
| 2017 | World Neurosurg | Health Care Costs of Spontaneous Aneurysmal Subarachnoid Hemorrhage for Rehabilitation, Home Care, and In-Hospital Treatment for the First Year |
| 2018 | Acta Anaesthesiol Scand | Patients' opinions on outcomes following critical illness |
| 2018 | Acta Anaesthesiologica Scandinavica | Health-related quality of life after general surgical intensive care |
| 2018 | Aging Clin Exp Res | Outcome of older persons admitted to intensive care unit, mortality, prognosis factors, dependency scores and ability trajectory within 1ﾃつyear: a prospective cohort study |
| 2018 | Am J Crit Care | Pilot Study Assessing the Impact of Bereavement Support on Families of Deceased Intensive Care Unit Patients |
| 2018 | Am J Respir Crit Care Med | The Burden of Specific Symptoms Reported by Survivors After Critical Illness |
| 2018 | Am J Respir Crit Care Med | Grief Symptoms in Relatives Who Experienced Organ Donation Requests in the ICU |
| 2018 | Am J Respir Crit Care Med | Effects of a Telephone- and Web-based Coping Skills Training Program Compared with an Education Program for Survivors of Critical Illness and Their Family Members. A Randomized Clinical Trial |
| 2018 | Am J Respir Crit Care Med | Prevalence, risk-factors, and outcomes of financial stress in survivors of critical illness |
| 2018 | Anesth Analg | Pain and Its Long-term Interference of Daily Life After Critical Illness |
| 2018 | Ann Am Thorac Soc | Computerized Cognitive Rehabilitation in Intensive Care Unit Survivors: Returning to Everyday Tasks Using Rehabilitation Networks-Computerized Cognitive Rehabilitation Pilot Investigation |
| 2018 | Arch Cardiol Mex | Early prognostic value of an Algorithm based on spectral Variables of Ventricular fibrillAtion from the EKG of patients with suddEn cardiac death: A multicentre observational study (AWAKE) |
| 2018 | Aust Crit Care | The impact on new-onset stress and PTSD in relatives of critically ill patients explored by diaries study (The "INSPIRED" study) |
| 2018 | BMC Med Res Methodol | Factors associated with missed assessments in a 2-year longitudinal study of acute respiratory distress syndrome survivors |
| 2018 | BMJ Open | Lessons learnt during the implementation of a web-based triage tool for Dutch intensive care follow-up clinics |
| 2018 | Chin J Traumatol | Long-term quality of life after sepsis and predictors of quality of life in survivors with sepsis |
| 2018 | Crit Care | Iron deficiency diagnosed using hepcidin on critical care discharge is an independent risk factor for death and poor quality of life at one year: an observational prospective study on 1161 patients |
| 2018 | Crit Care Med | Acute Kidney Injury and Subsequent Frailty Status in Survivors of Critical Illness: A Secondary Analysis |
| 2018 | Crit Care Med | Acute Physiologic Stress and Subsequent Anxiety Among Family Members of ICU Patients |
| 2018 | Crit Care Med | Evaluating Muscle Mass in Survivors of Acute Respiratory Distress Syndrome: A 1-Year Multicenter Longitudinal Study |
| 2018 | Crit Care Med | Early Risk and Resiliency Factors Predict Chronic Posttraumatic Stress Disorder in Caregivers of Patients Admitted to a Neuroscience ICU |
| 2018 | Crit Care Med | Shoulder Impairment Following Critical Illness: A Prospective Cohort Study |
| 2018 | Crit Care Med | Mental health-related quality of life is related to delirium in intensive care patients |
| 2018 | Crit Care Med | Co-Occurrence of Post-Intensive Care Syndrome Problems Among 406 Survivors of Critical Illness |
| 2018 | Crit Care Med | Temporal Trends in Healthcare Costs and Outcome Following ICU Admission After Traumatic Brain Injury |
| 2018 | Crit Care Med | Development and Validation of an Abbreviated Questionnaire to Easily Measure Cognitive Failure in ICU Survivors: A Multicenter Study |
| 2018 | Crit Care Med | Determinants of Health-Related Quality of Life After ICU: importance of Patient Demographics, Previous Comorbidity, and Severity of Illness |
| 2018 | Crit Care Med | Preventing Posttraumatic Stress in ICU Survivors: a Single-Center Pilot Randomized Controlled Trial of ICU Diaries and Psychoeducation |
| 2018 | Crit Care Res Pract | Outcome in Patients with Isolated Moderate to Severe Traumatic Brain Injury |
| 2018 | Crit Care Res Pract | Clinical Utility of a Structured Program to Reduce the Risk of Health-Related Quality of Life Impairment after Discharge from Intensive Care Unit: A Real-World Experience |
| 2018 | European journal of neurology | Status epilepticus associated with acute encephalitis: long-term follow-up of functional and cognitive outcomes in 72 patients |
| 2018 | Health Qual Life Outcomes | Fatigue in chronically critically ill patients following intensive care - reliability and validity of the multidimensional fatigue inventory (MFI-20) |
| 2018 | Heart Lung | Home discharge following critical illness: A qualitative analysis of family caregiver experience |
| 2018 | Injury | Long-term changes of patient-reported quality of life after major trauma: The importance of the time elapsed after injury |
| 2018 | Injury | Lasting impression of violence: Retained bullets and depressive symptoms |
| 2018 | Int J Hematol | Long-term outcomes in patients treated in the intensive care unit after hematopoietic stem cell transplantation |
| 2018 | Intensive Care Med | Psychiatric symptoms after acute respiratory distress syndrome: a 5-year longitudinal study |
| 2018 | Intensive Care Med | Relationships between markers of neurologic and endothelial injury during critical illness and long-term cognitive impairment and disability |
| 2018 | Intensive Care Med | Development of an ICU discharge instrument predicting psychological morbidity: a multinational study |
| 2018 | Intensive Care Med | Health-related outcomes of critically ill patients with and without sepsis |
| 2018 | Intensive Care Med | Association of social deprivation with 1-year outcome of ICU survivors: results from the FROG-ICU study |
| 2018 | Intensive Care Med | Premorbid functional status as a predictor of 1-year mortality and functional status in intensive care patients aged 80ﾃつyears or older |
| 2018 | Interact Cardiovasc Thorac Surg | Health-related quality of life of patients after surgery for acute Type A aortic dissection |
| 2018 | J Crit Care | Physical function impairment in survivors of critical illness in an ICU Recovery Clinic |
| 2018 | J Crit Care | Patients' perceptions and ICU clinicians predictions of quality of life following critical illness |
| 2018 | J Crit Care | Predictors of return to work in survivors of critical illness |
| 2018 | J Crit Care | Development of a prediction model for long-term quality of life in critically ill patients |
| 2018 | J Crit Care | Comprehensive care of ICU survivors: Development and implementation of an ICU recovery center |
| 2018 | J Crit Care | The ability of intensive care unit physicians to estimate long-term prognosis in survivors of critical illness |
| 2018 | J Crit Care | Robotic technology provides objective and quantifiable metrics of neurocognitive functioning in survivors of critical illness:A feasibility study |
| 2018 | J Crit Care | A multimodal rehabilitation program for patients with ICU acquired weakness improves ventilator weaning and discharge home |
| 2018 | J Int Med Res | Long-term sequelae of acute respiratory distress syndrome caused by severe community-acquired pneumonia: Delirium-associated cognitive impairment and post-traumatic stress disorder |
| 2018 | J Intensive Care | The impact of sepsis, delirium, and psychological distress on self-rated cognitive function in ICU survivors-a prospective cohort study |
| 2018 | J Intensive Care | Long-term recovery following critical illness in an Australian cohort |
| 2018 | J Neurosurg | Long-term and delayed functional recovery in patients with severe cerebrovascular and traumatic brain injury requiring tracheostomy |
| 2018 | Journal of the intensive care society | Supervised exercise rehabilitation in survivors of critical illness: a randomised controlled trial |
| 2018 | Lancet Respir Med | Clinical phenotypes of delirium during critical illness and severity of subsequent long-term cognitive impairment: a prospective cohort study |
| 2018 | Mayo Clin Proc Innov Qual Outcomes | Improving Quality of Life in Patients at Risk for Post-Intensive Care Syndrome |
| 2018 | Neurocrit Care | Critical West Nile Neuroinvasive Disease |
| 2018 | New England Journal of Medicine | A Randomized Trial of a Family-Support Intervention in Intensive Care Units |
| 2018 | Nurs Crit Care | Intensive care survivor-reported symptoms: a longitudinal study of survivors' symptoms |
| 2018 | Pain Manag Nurs | Prevalence, Location, and Characteristics of Chronic Pain in Intensive Care Survivors |
| 2018 | Palliat Support Care | Post-intensive care syndrome symptoms and health-related quality of life in family decision-makers of critically ill patients |
| 2018 | Pilot and feasibility studies | A feasibility study of a randomised controlled trial to examine the impact of the ABCDE bundle on quality of life in ICU survivors |
| 2018 | PloS one | Effects of early, combined endurance and resistance training in mechanically ventilated, critically ill patients: a randomised controlled trial |
| 2018 | PLoS One | Health-related quality of life in intensive care survivors: Associations with social support, comorbidity, and pain interference |
| 2018 | Respir Care | Physical Function in Subjects Requiring Extracorporeal Membrane Oxygenation Before or After Lung Transplantation |
| 2018 | Resuscitation | Surviving out-of-hospital cardiac arrest: The neurological and functional outcome and health-related quality of life one year later |
| 2018 | Rev Bras Ter Intensiva | Long-term psychological outcome after discharge from intensive care |
| 2018 | S Afr J Physiother | Changes in biopsychosocial outcomes for a mixed cohort of ICU survivors |
| 2018 | Vascular | Long-term outcome and quality of life after ruptured abdominal aortic aneurysm repair |
| 2019 | Acta Anaesthesiol Scand | Physical function and actigraphy in intensive care survivors-A prospective 3-month follow-up cohort study |
| 2019 | Acute Crit Care | The quality of dying and death for patients in intensive care units: a single center pilot study |
| 2019 | Am J Crit Care | Long-term Outcomes of Critically Ill Patients With Stroke Requiring Mechanical Ventilation |
| 2019 | Am J Crit Care | Exploring Patients' Goals Within the Intensive Care Unit Rehabilitation Setting |
| 2019 | Am J Crit Care | Validation of a New Clinical Tool for Post-Intensive Care Syndrome |
| 2019 | Am J Crit Care | Hospital Readmission and Subsequent Decline in Long-Term Survivors of Acute Respiratory Distress Syndrome |
| 2019 | Am J Crit Care | Stress Management Intervention to Prevent Post-Intensive Care Syndrome-Family in Patients' Spouses |
| 2019 | Am J Respir Crit Care Med | The Combined Effects of Frailty and Cognitive Impairment on Post-ICU Disability among Older ICU Survivors |
| 2019 | Am J Respir Crit Care Med | Long-Term Outcome after Prolonged Mechanical Ventilation. A Long-Term Acute-Care Hospital Study |
| 2019 | American Surgeon | Emergency Trauma Providers as Equal Partners: From "Proof of Concept" to "Outcome Parity" |
| 2019 | Ann Intensive Care | Incidence and impact of sepsis on long-term outcomes after subarachnoid hemorrhage: a prospective observational study |
| 2019 | Ann Intensive Care | Long-term neurocognitive outcome is not worsened by of the use of venovenous ECMO in severe ARDS patients |
| 2019 | Annals of surgery | Impact of Dexmedetomidine on Long-term Outcomes After Noncardiac Surgery in Elderly: 3-Year Follow-up of a Randomized Controlled Trial |
| 2019 | Annals of the American Thoracic Society | Physical Function Trajectories in Survivors of Acute Respiratory Failure |
| 2019 | Aust Crit Care | Quality of life in family caregivers of patients in the intensive care unit: A longitudinal study |
| 2019 | Can J Anaesth | I SURVIVE: inter-rater reliability of three physical functional outcome measures in intensive care unit survivors |
| 2019 | Chest | Determinants of Depressive Symptoms at 1 Year Following ICU Discharge in Survivors of ≥ 7 Days of Mechanical Ventilation: Results From the RECOVER Program, a Secondary Analysis of a Prospective Multicenter Cohort Study |
| 2019 | Clin J Am Soc Nephrol | Selection and Receipt of Kidney Replacement in Critically Ill Older Patients with AKI |
| 2019 | Clin Nurs Res | Functional Outcomes 6 Months After Severe Traumatic Brain Injury Following Admission Into Intensive Care Unit: A Cohort Study in Two Tertiary Hospitals |
| 2019 | Clinical Infectious Diseases | Long-term Quality of Life in Adult Patients Surviving Purpura Fulminans: An Exposed-Unexposed Multicenter Cohort Study |
| 2019 | Crit Care | Lack of clinically relevant correlation between subjective and objective cognitive function in ICU survivors: a prospective 12-month follow-up study |
| 2019 | Crit Care | Screening for posttraumatic stress disorder in ARDS survivors: validation of the Impact of Event Scale-6 (IES-6) |
| 2019 | Crit Care | Impact of post-traumatic stress symptoms on the health-related quality of life in a cohort study with chronically critically ill patients and their partners: age matters |
| 2019 | Crit Care Med | Cardiac Arrest and Subsequent Hospitalization-Induced Posttraumatic Stress Is Associated With 1-Year Risk of Major Adverse Cardiovascular Events and All-Cause Mortality |
| 2019 | Crit Care Med | The Development of Chronic Critical Illness Determines Physical Function, Quality of Life, and Long-Term Survival Among Early Survivors of Sepsis in Surgical ICUs |
| 2019 | Crit Care Med | Novel Risk Factors for Posttraumatic Stress Disorder Symptoms in Family Members of Acute Respiratory Distress Syndrome Survivors |
| 2019 | Crit Care Med | Caring for Critically Ill Patients with the ABCDEF Bundle: Results of the ICU Liberation Collaborative in Over 15,000 Adults |
| 2019 | Crit Care Med | Quality of Life and 1-Year Survival in Patients With Early Septic Shock: long-Term Follow-Up of the Australasian Resuscitation in Sepsis Evaluation Trial |
| 2019 | Crit Care Med | Fresh Red Cells for Transfusion in Critically Ill Adults: An Economic Evaluation of the Standard Issue Transfusion Versus Fresher Red-Cell Use in Intensive Care (TRANSFUSE) Clinical Trial |
| 2019 | Health Qual Life Outcomes | Quality of life reported by survivors after hospitalization for Middle East respiratory syndrome (MERS) |
| 2019 | Heart Lung | Exploring positive aspects of caregiving in family caregivers of adult ICU survivors from ICU to four months post-ICU discharge |
| 2019 | Heart, Lung & Circulation | Long-Term Survival and Health-Related Quality of Life in Adults After Extra Corporeal Membrane Oxygenation |
| 2019 | Indian J Crit Care Med | Quality of Life After Intensive Care Unit Discharge in a Tertiary Care Hospital in India: Cost Effectiveness Analyis |
| 2019 | Int J Gen Med | Psychiatric symptoms and their association with sleep disturbances in intensive care unit survivors |
| 2019 | Intensive & critical care nursing | Reprint of Recovery programme for ICU survivors has no effect on relatives' quality of life: secondary analysis of the RAPIT-study |
| 2019 | Intensive Care Med | Healthcare provider compassion is associated with lower PTSD symptoms among patients with life-threatening medical emergencies: a prospective cohort study |
| 2019 | Intensive Care Med | Mental illness after admission to an intensive care unit |
| 2019 | Intensive Care Med | Tailored multicomponent program for discomfort reduction in critically ill patients may decrease post-traumatic stress disorder in general ICU survivors at 1ﾃ・ﾂyear |
| 2019 | Intensive Care Med | Modifiable elements of ICU supportive care and communication are associated with surrogates' PTSD symptoms |
| 2019 | Intensive Crit Care Nurs | The health promoting conversations intervention for families with a critically ill relative: A pilot study |
| 2019 | Intensive Crit Care Nurs | Post-traumatic stress symptoms in family caregivers of intensive care unit patients: A longitudinal study |
| 2019 | J Crit Care | Incidence and risk factors for alopecia in survivors of critical illness: A multi-centre observational study |
| 2019 | J Crit Care | Psychocognitive sequelae of critical illness and correlation with 3 months follow up |
| 2019 | J Crit Care | Quality of life assessment following amputation for septic shock: a long-term descriptive survey after symmetric peripheral gangrene |
| 2019 | J Crit Care | Long term follow-up of quality of life and functional ability in patients with ICU acquired Weakness - A post hoc analysis |
| 2019 | J Intensive Care Med | Comorbidities Might Condition the Recovery of Quality of Life in Survivors of Sepsis |
| 2019 | J Rehabil Med | Chronic pain in intensive care unit survivors: incidence, characteristics and side-effects up to one-year post-discharge |
| 2019 | JAMA | Effect of an ICU Diary on Posttraumatic Stress Disorder Symptoms Among Patients Receiving Mechanical Ventilation: a Randomized Clinical Trial |
| 2019 | JAMA | Effect of a Nurse-Led Preventive Psychological Intervention on Symptoms of Posttraumatic Stress Disorder Among Critically Ill Patients: a Randomized Clinical Trial |
| 2019 | Journal of rehabilitation medicine | Can in-reach multidisciplinary rehabilitation in the acute ward improve outcomes for critical care survivors? A pilot randomized controlled trial |
| 2019 | Journal of the American Geriatrics Society | Early Post-Intensive Care Syndrome among Older Adult Sepsis Survivors Receiving Home Care |
| 2019 | Minerva Anestesiol | Long-term functional and psychological recovery in a population of acute respiratory distress syndrome patients treated with VV-ECMO and in their caregivers |
| 2019 | Mol Neurobiol | Predicting Long-term Cognitive Dysfunction in Survivors of Critical Illness with Plasma Inflammatory Markers: a Retrospective Cohort Study |
| 2019 | Perfusion | Long-term pulmonary function and quality of life in adults after extracorporeal membrane oxygenation for respiratory failure |
| 2019 | PLoS One | The prognostic value of neurofilament levels in patients with sepsis-associated encephalopathy - A prospective, pilot observational study |
| 2019 | PLoS One | Mental health and quality of life outcomes in family members of patients with chronic critical illness admitted to the intensive care units of two Brazilian hospitals serving the extremes of the socioeconomic spectrum |
| 2019 | PLoS One | Simple functional assessment at hospital discharge can predict long-term outcomes of ICU survivors |
| 2019 | Psychosomatics | Deficits in Self-Reported Initiation Are AssociatedWith Subsequent Disability in ICU Survivors |
| 2019 | Rev Bras Ter Intensiva | Adequacy of enteral nutritional support in intensive care units does not affect the short- and long-term prognosis of mechanically ventilated patients: a pilot study |
| 2019 | Semin Thorac Cardiovasc Surg | Patients With a Prolonged Intensive Care Unit Length of Stay Have Decreased Health-Related Quality of Life After Cardiac Surgery |
| 2019 | Sleep Breath | Sleep quality in survivors of critical illness |
| 2019 | Thorax | Effects of mindfulness training programmes delivered by a self-directed mobile app and by telephone compared with an education programme for survivors of critical illness: a pilot randomised clinical trial |
| 2019 | Thorax | Five-year mortality and morbidity impact of prolonged versus brief ICU stay: a propensity score matched cohort study |
| 2020 | Acta Anaesthesiol Scand | Predictive variables for poor long-term physical recovery after intensive care unit stay: An exploratory study |
| 2020 | Acta Anaesthesiol Scand | Cognitive function and health-related quality of life 1 year after acute brain injury: An observational study |
| 2020 | Acta Anaesthesiol Scand | Non-sedation-Does it improve health-related quality of life after critical illness? A 3-month follow-up sub-study of the NONSEDA trial |
| 2020 | Acta Anaesthesiologica Scandinavica | ICU discharge screening for prediction of new-onset physical disability-A multinational cohort study |
| 2020 | Acta anaesthesiologica Scandinavica | Effect of non-sedation on post-traumatic stress and psychological health in survivors of critical illness-A substudy of the NONSEDA randomized trial |
| 2020 | Acute Med Surg | Activities of daily living status and psychiatric symptoms after discharge from an intensive care unit: a single-center 12-month longitudinal prospective study |
| 2020 | Am J Crit Care | Depression and Change in Caregiver Burden Among Family Members of Intensive Care Unit Survivors |
| 2020 | Am J Nurs | Original Research: Exploring the Effects of a Nurse-Initiated Diary Intervention on Post-Critical Care Posttraumatic Stress Disorder |
| 2020 | Am J Phys Med Rehabil | Feasibility and Efficacy of Cardiopulmonary Rehabilitation After COVID-19 |
| 2020 | Am J Respir Crit Care Med | Feasibility and acceptability of a self-managed exercise to rhythmic music intervention for ICU survivors |
| 2020 | Am J Surg | Abdominal sepsis patients have a high incidence of chronic critical illness with dismal long-term outcomes |
| 2020 | American Journal of Medicine | Long-Term Courses of Sepsis Survivors: Effects of a Primary Care Management Intervention |
| 2020 | Anaesth Crit Care Pain Med | Three-month quality of life in survivors of ARDS due to COVID-19: A preliminary report from a French academic centre |
| 2020 | Ann Intensive Care | Tracheostomy in patients with acute respiratory distress syndrome is not related to quality of life, symptoms of psychiatric disorders or return-to-work: the prospective DACAPO cohort study |
| 2020 | Ann Intensive Care | Cognitive and psychosocial outcomes of mechanically ventilated intensive care patients with and without delirium |
| 2020 | Ann Ist Super Sanita | Long-term consequences in survivors of critical illness. Analysis of incidence and risk factors |
| 2020 | Ann Surg | Socioeconomic Factors and Intensive Care Unit-Related Cognitive Impairment |
| 2020 | Asaio j | Long-Term Health-Related Quality of Life After Venovenous Extracorporeal Membrane Oxygenation |
| 2020 | Aust Crit Care | The sedentary behaviour and physical activity patterns of survivors of a critical illness over their acute hospitalisation: An observational study |
| 2020 | Aust Crit Care | Patient experience of necrotising soft-tissue infection from diagnosis to six months after intensive care unit stay: A qualitative content analysis |
| 2020 | Aust Crit Care | An observational study investigating the use of patient-owned technology to quantify physical activity in survivors of critical illness |
| 2020 | Aust Crit Care | The fear and risk of community falls in patients following an intensive care admission: An exploratory cohort study |
| 2020 | Australian Critical Care | Feasibility and acceptability of conducting a partially randomised controlled trial examining interventions to improve psychological health after discharge from the intensive care unit |
| 2020 | Australian Critical Care | The needs of patients with post-intensive care syndrome: A prospective, observational study |
| 2020 | Australian critical care | The effect of family-authored diaries on posttraumatic stress disorder in intensive care unit patients and their relatives: a randomised controlled trial (DRIP-study) |
| 2020 | BJGP Open | Health problems among family caregivers of former intensive care unit (ICU) patients: an interview study |
| 2020 | BMC Public Health | Influence of quality of intensive care on quality of life/return to work in survivors of the acute respiratory distress syndrome: prospective observational patient cohort study (DACAPO) |
| 2020 | Br J Dermatol | Health-related quality of life and long-term sequelae in survivors of epidermal necrolysis: an observational study of 57 patients |
| 2020 | Can J Anaesth | A qualitative study of bereaved family members with complicated grief following a death in the intensive care unit |
| 2020 | Chest | Emotional Experiences and Coping Strategies of Family Members of Critically Ill Patients |
| 2020 | Clin Nurs Res | Functional Status in Older Intensive Care Unit Survivors |
| 2020 | Crit Care | Cognitive phenotypes 1 month after ICU discharge in mechanically ventilated patients: a prospective observational cohort study |
| 2020 | Crit Care | Implications for post critical illness trial design: sub-phenotyping trajectories of functional recovery among sepsis survivors |
| 2020 | Crit Care | Effect of an ICU diary on psychiatric disorders, quality of life, and sleep quality among adult cardiac surgical ICU survivors: a randomized controlled trial |
| 2020 | Crit Care Explor | ICU Recovery Clinic Attendance, Attrition, and Patient Outcomes: The Impact of Severity of Illness, Gender, and Rurality |
| 2020 | Crit Care Med | Family Care Rituals in the ICU to Reduce Symptoms of Post-Traumatic Stress Disorder in Family Members-A Multicenter, Multinational, Before-and-After Intervention Trial |
| 2020 | Crit Care Med | Prevalence and Course of Frailty in Survivors of Critical Illness |
| 2020 | Crit Care Med | Association of Hypoactive and Hyperactive Delirium With Cognitive Function After Critical Illness |
| 2020 | Crit Care Med | National Trends and Variation of Functional Status Deterioration in the Medically Critically Ill |
| 2020 | Crit Care Med | Occurrence and Risk Factors of Chronic Pain After Critical Illness |
| 2020 | Crit Care Med | Evaluating Physical Functioning in Survivors of Critical Illness: Development of a New Continuum Measure for Acute Care |
| 2020 | Crit Care Nurse | Feasibility of Nurse-Led Multidimensional Outcome Assessments in the Neuroscience Intensive Care Unit |
| 2020 | Crit Care Resusc | Autonomic function, postprandial hypotension and falls in older adults at one year after critical illness |
| 2020 | Front Med (Lausanne) | Acute Functional Outcomes in Critically Ill COVID-19 Patients |
| 2020 | Front Neurosci | The Association Between Brain Volumes and Posttraumatic Stress Disorder in Intensive Care Unit Survivors: A Preliminary Study |
| 2020 | General Hospital Psychiatry | Baseline resilience and depression symptoms predict trajectory of depression in dyads of patients and their informal caregivers following discharge from the Neuro-ICU |
| 2020 | General hospital psychiatry | Implementing an intensive care unit (ICU) diary program at a large academic medical center: results from a randomized control trial evaluating psychological morbidity associated with critical illness |
| 2020 | Ger Med Sci | The quality of acute intensive care and the incidence of critical events have an impact on health-related quality of life in survivors of the acute respiratory distress syndrome - a nationwide prospective multicenter observational study |
| 2020 | Heart Vessels | Cognitive function in post-cardiac intensive care: patient characteristics and impact of multidisciplinary cardiac rehabilitation |
| 2020 | Indian J Crit Care Med | Neurocognitive and Quality-of-life Outcomes Following Intensive Care Admission: A Prospective 6-month Follow-up Study |
| 2020 | Indian J Crit Care Med | Caloric Adequacy in the First Week of Mechanically Ventilated Patients has No Impact on Long-term Daily Life Activities |
| 2020 | Indian J Crit Care Med | Health-related Quality of Life Evaluated by MOS SF-36 in the Elderly Patients 1 Month before ICU Admission and 3 Months after ICU Discharge |
| 2020 | Intensive Care Med | Extracorporeal membrane oxygenation for refractory cardiac arrest: a retrospective multicenter study |
| 2020 | Intensive Care Med | Five-year impact of ICU-acquired neuromuscular complications: a prospective, observational study |
| 2020 | Intensive Care Med | Health-related quality of life in survivors of septic shock: 6-month follow-up from the ADRENAL trial |
| 2020 | Intensive Care Med Exp | Intensive Care specific Virtual Reality (ICU-VR) improves Post-Intensive Care Syndrome-related psychological sequelae in survivors of critical illness |
| 2020 | Intensive Crit Care Nurs | Support needs and health-related quality of life of family caregivers of patients requiring prolonged mechanical ventilation and admission to a specialised weaning centre: A qualitative longitudinal interview study |
| 2020 | International journal of clinical and experimental medicine | The effect of family empowerment nursing on severe neurosurgical patients |
| 2020 | International Psychogeriatrics | Critical Care Recovery Center: a model of agile implementation in intensive care unit (ICU) survivors |
| 2020 | J Am Geriatr Soc | Older Sepsis Survivors Suffer Persistent Disability Burden and Poor Long-Term Survival |
| 2020 | J Clin Neurosci | Six-month mortality and functional outcomes in aneurysmal sub-arachnoid haemorrhage patients admitted to intensive care units in Australia and New Zealand: A prospective cohort study |
| 2020 | J Crit Care | Neuropsychiatric outcome in subgroups of Intensive Care Unit survivors: Implications for after-care |
| 2020 | J Crit Care | Psychotropic medication use in former ICU patients with mental health problems: A prospective observational follow-up study |
| 2020 | J Crit Care | Defining patient-centered recovery after critical illness - A qualitative study |
| 2020 | J Head Trauma Rehabil | The Experience of Caregivers Following a Moderate to Severe Traumatic Brain Injury Requiring ICU Admission |
| 2020 | J Intensive Care | Patients suffering from psychological impairments following critical illness are in need of information |
| 2020 | J Intensive Care Med | Frailty, Acute Organ Dysfunction, and Increased Disability After Hospitalization in Older Adults Who Survive Critical Illness: A Prospective Cohort Study |
| 2020 | J Nurs Scholarsh | Gender Differences Regarding the Impact of Change in Cognitive Function on the Functional Status of Intensive Care Unit Survivors: A Prospective Cohort Study |
| 2020 | J Spinal Cord Med | Caregiver expectations of recovery among persons with spinal cord injury at three and six months post-injury: A brief report |
| 2020 | J Trauma Acute Care Surg | The Center for Trauma Survivorship: Addressing the great unmet need for posttrauma center care |
| 2020 | JAMA Network Open | Risk Factors for Long-term Mortality and Patterns of End-of-Life Care Among Medicare Sepsis Survivors Discharged to Home Health Care |
| 2020 | JAMA Network Open | Feasibility and Efficacy of a Resiliency Intervention for the Prevention of Chronic Emotional Distress Among Survivor-Caregiver Dyads Admitted to the Neuroscience Intensive Care Unit: A Randomized Clinical Trial |
| 2020 | JAMA Surg | Long-term Functional, Psychological, Emotional, and Social Outcomes in Survivors of Firearm Injuries |
| 2020 | Jpn J Nurs Sci | Post-intensive care unit depression among critical care survivors: A nationwide population-based study |
| 2020 | Med Klin Intensivmed Notfmed | Quality improvement of end‑of‑life decision-making and communication in the ICU |
| 2020 | Medicine (Baltimore) | The association between depression and length of stay in the intensive care unit |
| 2020 | Neurocrit Care | Gender Differences in Longitudinal Associations Between Intimate Care, Resiliency, and Depression Among Informal Caregivers of Patients Surviving the Neuroscience Intensive Care Unit |
| 2020 | Neurocrit Care | The Impact of Resilience Factors and Anxiety During Hospital Admission on Longitudinal Anxiety Among Dyads of Neurocritical Care Patients Without Major Cognitive Impairment and Their Family Caregivers |
| 2020 | Nitric oxide : biology and chemistry | A randomized pilot study of nitrate supplementation with beetroot juice in acute respiratory failure |
| 2020 | Physiotherapy | Patient and family experience of physical rehabilitation on the intensive care unit: a qualitative exploration |
| 2020 | Pilot and feasibility studies | Recovering together: building resiliency in dyads of stroke patients and their caregivers at risk for chronic emotional distress; A feasibility study |
| 2020 | PLoS One | Patient-reported physical functioning is limited in almost half of critical illness survivors 1-year after ICU-admission: A retrospective single-centre study |
| 2020 | PloS one | Functional ability and quality of life in critical illness survivors with intensive care unit acquired weakness: a secondary analysis of a randomised controlled trial |
| 2020 | PLoS One | One year after ICU admission for severe community-acquired pneumonia of bacterial, viral or unidentified etiology. What are the outcomes? |
| 2020 | Psychosomatics | Baseline Resilience and Posttraumatic Symptoms in Dyads of Neurocritical Patients and Their Informal Caregivers: A Prospective Dyadic Analysis |
| 2020 | Resuscitation | One-year outcome of patients admitted after cardiac arrest compared to other causes of ICU admission. An ancillary analysis of the observational prospective and multicentric FROG-ICU study |
| 2020 | West J Emerg Med | Post-traumatic Stress Disorder in Family-witnessed Resuscitation of Emergency Department Patients |
| 2020 | World J Emerg Surg | Long-term outcomes in major trauma patients and correlations with the acute phase |
| 2021 | Acta Anaesthesiol Scand | Risk factors for long-term cognitive impairment in ICU survivors: A multicenter, prospective cohort study |
| 2021 | Acta Anaesthesiol Scand | Two-months quality of life of COVID-19 invasively ventilated survivors; an Italian single-center study |
| 2021 | Acta Anaesthesiol Scand | Long-term consequences in critically ill COVID-19 patients: A prospective cohort study |
| 2021 | Acta Med Port | Health-Related Quality of Life in Survivors of Severe COVID-19 of a University Hospital in Northern Portugal |
| 2021 | Am J Crit Care | What Matters to Patients and Their Families During and After Critical Illness: A Qualitative Study |
| 2021 | Am J Crit Care | Feasibility of a Home-Based Palliative Care Intervention for Elderly Multimorbid Survivors of Critical Illness |
| 2021 | Am J Crit Care | Benefits of Peer Support for Intensive Care Unit Survivors: Sharing Experiences, Care Debriefing, and Altruism |
| 2021 | Am J Med Sci | Muscle Power is Related to Physical Function in Patients Surviving Acute Respiratory Failure: A Prospective Observational Study |
| 2021 | Am J Respir Crit Care Med | High Prevalence of Pulmonary Sequelae at 3 Months after Hospital Discharge in Mechanically Ventilated Survivors of COVID-19 |
| 2021 | Ann Am Thorac Soc | Functional Outcomes, Goals, and Goal Attainment among Chronically Critically Ill Long-Term Acute Care Hospital Patients |
| 2021 | Ann Am Thorac Soc | Detection of Cognitive Impairment after Critical Illness with the Medicare Annual Wellness Visit: A Cohort Study |
| 2021 | Ann Intensive Care | Short-term health-related quality of life, physical function and psychological consequences of severe COVID-19 |
| 2021 | Ann Intensive Care | Determinants of hospital and one-year mortality among older patients admitted to intensive care units: results from the multicentric SENIOREA cohort |
| 2021 | Ann Intensive Care | Health-related quality of life in critically ill survivors: specific impact of cardiac arrest in non-shockable rhythm |
| 2021 | Ann Intensive Care | Lipid and lipoprotein predictors of functional outcomes and long-term mortality after surgical sepsis |
| 2021 | Ann Intensive Care | Post-intensive care syndrome after a critical COVID-19: cohort study from a Belgian follow-up clinic |
| 2021 | Ann Intensive Care | Long-term quality of life in necrotizing soft-tissue infection survivors: a monocentric prospective cohort study |
| 2021 | Ann Palliat Med | Neurocognitive improvement after angioplasty in patients with chronic middle cerebral artery stenosis and cerebral ischemia |
| 2021 | Aust Crit Care | The effect of postintensive care syndrome on the quality of life of intensive care unit survivors: A secondary analysis |
| 2021 | Aust Crit Care | Impact of a nurse-led family support intervention on family members' satisfaction with intensive care and psychological wellbeing: A mixed-methods evaluation |
| 2021 | Australian Critical Care | Quadriceps strength in intensive care unit survivors: Variability and influence of preadmission physical activity |
| 2021 | Biopsychosoc Med | Factors influencing post-ICU psychological distress in family members of critically ill patients: a linear mixed-effects model |
| 2021 | BMC Anesthesiology | Muscle weakness, functional capacities and recovery for COVID-19 ICU survivors |
| 2021 | BMC medicine | Nocturnal dexmedetomidine alleviates post-intensive care syndrome following cardiac surgery: a prospective randomized controlled clinical trial |
| 2021 | BMC Nephrol | Epidemiology and outcomes of elderly patients requiring renal replacement therapy in the intensive care unit: an observational study |
| 2021 | BMC Psychiatry | Symptoms of post-traumatic stress disorder (PTSD) in next of kin during suspension of ICU visits during the COVID-19 pandemic: a prospective observational study |
| 2021 | Bmj | Suicide and self-harm in adult survivors of critical illness: population based cohort study |
| 2021 | BMJ Open | Delirium and neuropsychological outcomes in critically Ill patients with COVID-19: a cohort study |
| 2021 | BMJ Open Respir Res | Long-term outcomes following severe COVID-19 infection: a propensity matched cohort study |
| 2021 | Brain Behav | Long-term mortality associated with depression among South Korean survivors of extracorporeal membrane oxygenation |
| 2021 | Burns | The initial validation of a novel outcome measure in severe burns- the Persistent Organ Dysfunction +Death: Results from a multicenter evaluation |
| 2021 | Chest | Pulmonary Function and Radiologic Features in Survivors of Critical COVID-19: A 3-Month Prospective Cohort |
| 2021 | Chest | Long-Term Disabilities of Survivors of Out-of-Hospital Cardiac Arrest: The Hanox Study |
| 2021 | Chest | Association of Job Characteristics and Functional Impairments on Return to Work After ARDS |
| 2021 | Chest | The Burden of Mental Illness Among Survivors of Critical Care-Risk Factors and Impact on Quality of Life: A Multicenter Prospective Cohort Study |
| 2021 | Chest | Sleep Fragmentation and Cognitive Trajectories After Critical Illness |
| 2021 | Clin Nutr | Impact of COVID-19 in nutritional and functional status of survivors admitted in intensive care units during the first outbreak. Preliminary results of the NUTRICOVID study |
| 2021 | Clin Nutr | Nutritional Risk at intensive care unit admission and outcomes in survivors of critical illness |
| 2021 | Crit Care | The impact of COVID-19 critical illness on new disability, functional outcomes and return to work at 6 months: a prospective cohort study |
| 2021 | Crit Care | Prevalence of post-intensive care syndrome among Japanese intensive care unit patients: a prospective, multicenter, observational J-PICS study |
| 2021 | Crit Care | Feasibility of a home-based interdisciplinary rehabilitation program for patients with Post-Intensive Care Syndrome: the REACH study |
| 2021 | Crit Care | Long-term survival and health-related quality of life in patients with severe acute respiratory distress syndrome and veno-venous extracorporeal membrane oxygenation support |
| 2021 | Crit Care | Long-term health-related quality of life and burden of disease after intensive care: developmentﾃつof a patient-reported outcome measure |
| 2021 | Crit Care | Course and predictors of posttraumatic stress-related symptoms among family members of deceased ICU patients during the first year of bereavement |
| 2021 | Crit Care | Trajectories of depression in sepsis survivors: an observational cohort study |
| 2021 | Crit Care Explor | One-Year Outcomes of Postintensive Care Syndrome in Critically Ill Coronavirus Disease 2019 Patients: A Single Institutional Study |
| 2021 | Crit Care Explor | Long-Term Cognitive Outcomes and Sleep in Adults After Extracorporeal Life Support |
| 2021 | Crit Care Explor | Psychological Symptoms in Relatives of Critically Ill Patients: A Longitudinal Cohort Study |
| 2021 | Crit Care Explor | Safety and Feasibility of an Interdisciplinary Treatment Approach to Optimize Recovery From Critical Coronavirus Disease 2019 |
| 2021 | Crit Care Explor | One-Year Functional, Cognitive, and Psychological Outcomes Following the Use of Extracorporeal Membrane Oxygenation in Coronavirus Disease 2019: A Prospective Study |
| 2021 | Crit Care Explor | Impact of a Visual Support Dedicated to Prognosis on Symptoms of Stress of ICU Family Members: A Before-and-After Implementation Study |
| 2021 | Crit Care Explor | Psychologic Distress and Quality of Life After ICU Treatment for Coronavirus Disease 2019: A Multicenter, Observational Cohort Study |
| 2021 | Crit Care Explor | Virtual Reality to Improve Sequelae of the Postintensive Care Syndrome: A Multicenter, Randomized Controlled Feasibility Study |
| 2021 | Crit Care Explor | Posttraumatic Stress Disorder Symptom Trajectories in ICU Family Caregivers |
| 2021 | Crit Care Med | Return to Work After Coronavirus Disease 2019 Acute Respiratory Distress Syndrome and Intensive Care Admission: Prospective, Case Series at 6 Months From Hospital Discharge |
| 2021 | Crit Care Med | Postintensive Care Syndrome in Survivors of Critical Illness Related to Coronavirus Disease 2019: Cohort Study From a New York City Critical Care Recovery Clinic |
| 2021 | Crit Care Med | Mobilization During Critical Illness: A Higher Level of Mobilization Improves Health Status at 6 Months, a Secondary Analysis of a Prospective Cohort Study |
| 2021 | Crit Care Med | Motoric Subtypes of Delirium and Long-Term Functional and Mental Health Outcomes in Adults After Critical Illness |
| 2021 | Crit Care Med | ICU Memories and Patient Outcomes in a Low Middle-Income Country: A Longitudinal Cohort Study |
| 2021 | Crit Care Med | Functional Outcomes and Their Association With Physical Performance in Mechanically Ventilated Coronavirus Disease 2019 Survivors at 3 Months Following Hospital Discharge: A Cohort Study |
| 2021 | Crit Care Med | End-of-Life-Care Quality in ICUs Is Associated With Family Surrogates' Severe Anxiety and Depressive Symptoms During Their First 6 Months of Bereavement |
| 2021 | Crit Care Med | Adapting to a New Normal After Severe Acute Brain Injury: An Observational Cohort Using a Sequential Explanatory Design |
| 2021 | Cureus | Pulmonary Function, Mental and Physical Health in Recovered COVID-19 Patients Requiring Invasive Versus Non-invasive Oxygen Therapy: A Prospective Follow-Up Study Post-ICU Discharge |
| 2021 | Cureus | Feasibility of Contrasting Brain Connectivity Patterns in Cognitive and Motor Cerebral Networks to Clinical Outcomes in Patients Surviving Acute Respiratory Failure: A Pilot Study |
| 2021 | ERJ Open Res | Chest radiography is a poor predictor of respiratory symptoms and functional impairment in survivors of severe COVID-19 pneumonia |
| 2021 | Eur J Neurol | Permanent loss of independence in adult febrile-infection-related epilepsy syndrome survivors: an underestimated and unsolved challenge |
| 2021 | Eur J Phys Rehabil Med | Midterm functional sequelae and implications in rehabilitation after COVID-19: a cross-sectional study |
| 2021 | Eur J Phys Rehabil Med | Comprehensive rehabilitation treatment for sub-acute COVID-19 patients: an observational study |
| 2021 | Eur J Phys Rehabil Med | Physical rehabilitation in Intensive Care Unit in acute respiratory distress syndrome patients with COVID-19 |
| 2021 | Eur Respir J | Dyspnoea, lung function and CT findings 3 months after hospital admission for COVID-19 |
| 2021 | Fam Syst Health | Family health conversations versus support group conversations when a family member has been critically ill: A mixed methods study |
| 2021 | Front Med (Lausanne) | The Functional Trajectory in Frail Compared With Non-frail Critically Ill Patients During the Hospital Stay |
| 2021 | Front Psychiatry | Association Between Psychological Distress, Cognitive Complaints, and Neuropsychological Status After a Severe COVID-19 Episode: A Cross-Sectional Study |
| 2021 | Healthcare (Basel) | Six-Month Outcomes in COVID-19 ICU Patients and Their Family Members: A Prospective Cohort Study |
| 2021 | Healthcare (Basel) | Quality of Life, Depression, and Anxiety in Survivors of Critical Illness from a Greek ICU. A Prospective Observational Study |
| 2021 | Heart Lung | Post-traumatic stress disorder symptoms after veno-arterial extracorporeal membrane oxygenator support |
| 2021 | Int J Environ Res Public Health | Six Months Follow-Up of Patients with Invasive Mechanical Ventilation due to COVID-19 Related ARDS |
| 2021 | Int J Environ Res Public Health | Men's Positive and Negative Experiences Following Acute Myocardial Infarction |
| 2021 | Int J Environ Res Public Health | Implementation of a Follow-Up Program for Intensive Care Unit Survivors |
| 2021 | Intensive & Critical Care Nursing | Older patients' recovery following intensive care: A follow-up study with the RAIN questionnaire |
| 2021 | Intensive & critical care nursing | VidaTalk™ patient communication application "opened up" communication between nonvocal ICU patients and their family |
| 2021 | Intensive Care Med | High prevalence of acute stress disorder and persisting symptoms in ICU survivors after COVID-19 |
| 2021 | Intensive Care Med | The 5-year pre- and post-hospitalization treated prevalence of mental disorders and psychotropic medication use in critically ill patients: a Canadian population-based study |
| 2021 | Intensive Care Med Exp | Intensive Care Unit-specific Virtual Reality for COVID-19 ICU survivors |
| 2021 | Intensive Crit Care Nurs | Effect of relatives' intensive care unit diaries on post traumatic stress in patients and relatives (DRIP-study): A mixed methods study |
| 2021 | Intensive Crit Care Nurs | Incidence and influencing factors of post-intensive care cognitive impairment |
| 2021 | J Adv Nurs | Patients' experiences of recovery: Beyond the intensive care unit and into the community |
| 2021 | J Cachexia Sarcopenia Muscle | The impact of sarcopenia and acute muscle mass loss on long-term outcomes in critically ill patients with intra-abdominal sepsis |
| 2021 | J Clin Med | Effectiveness of a Primary Care Telerehabilitation Program for Post-COVID-19 Patients: A Feasibility Study |
| 2021 | J Clin Med | Longitudinal Assessment of Health and Quality of Life of COVID-19 Patients Requiring Intensive Care-An Observational Study |
| 2021 | J Clin Med | A Cognitive Behavioral Therapy-Informed Self-Management Program for Acute Respiratory Failure Survivors: A Feasibility Study |
| 2021 | J Clin Med | Roles of Early Mobilization Program in Preventing Muscle Weakness and Decreasing Psychiatric Disorders in Patients with Coronavirus Disease 2019 Pneumonia: A Retrospective Observational Cohort Study |
| 2021 | J Clin Med | Grip Strength Correlates with Mental Health and Quality of Life after Critical Care: A Retrospective Study in a Post-Intensive Care Syndrome Clinic |
| 2021 | J Clin Med | Effect of Early Rehabilitation on Physical Function in Patients Undergoing Coronary Artery Bypass Grafting: A Nationwide Inpatient Database Study |
| 2021 | J Clin Nurs | Psychological responses and coping behaviour of visiting family members during and following unplanned hospital admission |
| 2021 | J Crit Care | Combination of delirium and coma predicts psychiatric symptoms at twelve months in critically ill patients: A longitudinal cohort study |
| 2021 | J Crit Care | Development of a practically usable prediction model for quality of life of ICU survivors: A sub-analysis of the MONITOR-IC prospective cohort study |
| 2021 | J Crit Care | Frequency and risk factors of post-intensive care syndrome components in a multicenter randomized controlled trial of German sepsis survivors |
| 2021 | J Crit Care | Effect of non-sedation on physical function in survivors of critical illness - A substudy of the NONSEDA randomized trial |
| 2021 | J Hosp Palliat Nurs | Feasibility of an Intervention Study to Support Families When Their Loved One Has Life-sustaining Therapy Withdrawn |
| 2021 | J Int Med Res | Heart rate variability and subsequent psychological distress among family members of intensive care unit patients |
| 2021 | J Intensive Care | Association between intensive care unit admission of a patient and mental disorders in the spouse: a retrospective matched-pair cohort study |
| 2021 | J Intensive Care | Functional status of mechanically ventilated COVID-19 survivors at ICU and hospital discharge |
| 2021 | J Intensive Care Med | Postseptic Cognitive Impairment and Expression of APOE in Peripheral Blood: The Cognition After SepsiS (CASS) Observational Pilot Study |
| 2021 | J Intensive Care Med | Incidence, Time Course and Influence on Quality of Life of Intensive Care Unit-Acquired Weakness Symptoms in Long-Term Intensive Care Survivors |
| 2021 | J Intensive Care Med | Anemia in Critically Ill Patients With Acute Respiratory Distress Syndrome and Posthospitalization Physical Outcomes |
| 2021 | J Med Virol | Postdischarge symptoms and rehabilitation needs in survivors of COVID-19 infection: A cross-sectional evaluation |
| 2021 | J Nerv Ment Dis | COVID-19 and Cognitive, Emotional Aspects of Post-Intensive Care Syndrome |
| 2021 | J Psychiatr Res | Depression and mortality among survivors of acute respiratory distress syndrome in South Korea: A nationwide cohort study conducted from 2010 to 2018 |
| 2021 | J Rehabil Med | Post-intensive care syndrome following cardiothoracic critical care: Feasibility of a complex intervention |
| 2021 | J Thorac Dis | Functional disability and post-traumatic stress disorder in survivors of mechanical ventilation: a cross-sectional study in Guangzhou, China |
| 2021 | J Trauma Acute Care Surg | High occurrence of postintensive care syndrome identified in surgical ICU survivors after implementation of a multidisciplinary clinic |
| 2021 | J Trauma Acute Care Surg | Depression predicts long-term cognitive impairment in survivors of critical illness |
| 2021 | JAMA | Four-Month Clinical Status of a Cohort of Patients After Hospitalization for COVID-19 |
| 2021 | JMIR Aging | Using Consumer-Grade Physical Activity Trackers to Measure Frailty Transitions in Older Critical Care Survivors: Exploratory Observational Study |
| 2021 | Journal of Advanced Nursing (John Wiley & Sons, Inc.) | Exploring adult critical illness survivors' experiences of fatigue: A qualitative study |
| 2021 | Journal of Hospice & Palliative Nursing | Post-Intensive Care Syndrome in Covid-19 Patients Discharged From the Intensive Care Unit |
| 2021 | Journal of personalized medicine | Virtual Reality-Based Early Neurocognitive Stimulation in Critically Ill Patients: a Pilot Randomized Clinical Trial |
| 2021 | Journal of Psychosomatic Research | Bodily pain in survivors of acute respiratory distress syndrome: A 1-year longitudinal follow-up study |
| 2021 | Lancet Psychiatry | 6-month neurological and psychiatric outcomes in 236 379 survivors of COVID-19: a retrospective cohort study using electronic health records |
| 2021 | Lancet Respir Med | Nebulised heparin for patients with or at risk of acute respiratory distress syndrome: a multicentre, randomised, double-blind, placebo-controlled phase 3 trial |
| 2021 | Medical-Surgical Nursing Journal | Effect of Home-based Pulmonary Rehabilitation on Fatigue, Dyspnea, and Activities of Daily Living of COVID-19 Patients |
| 2021 | Neurocrit Care | Can a Dyadic Resiliency Program Improve Quality of Life in Cognitively Intact Dyads of Neuro-ICU Survivors and Informal Caregivers? Results from a Pilot RCT |
| 2021 | Neurol Res | Functional outcome after critical illness in older patients: a population-based study |
| 2021 | Neurol Sci | Propofol shows less negative effects on cognitive performances than dexmedetomidine in elderly intensive care unit patients |
| 2021 | Nurs Crit Care | Psychological symptoms in difficult-to-sedate critical care survivors |
| 2021 | Nurs Crit Care | Pilot exploration of post-traumatic stress symptoms in intensive care unit survivors in Cyprus |
| 2021 | Nursing in Critical Care | Postﾂ・E・・ｬﾂ・Etraumatic stress disorder in critical illness survivors and its relation to memories of ICU |
| 2021 | Phys Ther | Cardiopulmonary Exercise Testing to Assess Persistent Symptoms at 6 Months in People With COVID-19 Who Survived Hospitalization: A Pilot Study |
| 2021 | Phys Ther | Activity Levels in Survivors of the Intensive Care Unit |
| 2021 | Phys Ther | Safety, Feasibility, and Outcomes of Frequent, Long-Duration Rehabilitation in an Inpatient Rehabilitation Facility After Prolonged Hospitalization for Severe COVID-19: An Observational Study |
| 2021 | PLoS One | Prevalence of and risk factors for post-intensive care syndrome: Multicenter study of patients living at home after treatment in 12 Japanese intensive care units, SMAP-HoPe study |
| 2021 | PLoS One | Post-intensive care syndrome as a predictor of mortality in patients with critical illness: A cohort study |
| 2021 | Qual Life Res | Quality of life of COVID-19 critically ill survivors after ICU discharge: 90 days follow-up |
| 2021 | Respir Med | Integrative respiratory follow-up of severe COVID-19 reveals common functional and lung imaging sequelae |
| 2021 | Respir Med | Health-related quality of life profiles, trajectories, persistent symptoms and pulmonary function one year after ICU discharge in invasively ventilated COVID-19 patients, a prospective follow-up study |
| 2021 | Respir Med | Long-term cognitive and psychiatric outcomes of acute respiratory distress syndrome managed with Extracorporeal Membrane Oxygenation |
| 2021 | Respir Med | Clinical outcomes and quality of life of COVID-19 survivors: A follow-up of 3 months post hospital discharge |
| 2021 | Resuscitation | Biomarker prognostication of cognitive impairment may be feasible even in out-of hospital cardical arrest survivors with good neurological outcome |
| 2021 | Rev Bras Ter Intensiva | The 6-Minute Walk Test predicts long-term physical improvement among intensive care unit survivors: a prospective cohort study |
| 2021 | Sci Rep | Health-related quality of life in ICU survivors-10 years later |
| 2021 | Sci Rep | Characterizing non-critically ill COVID-19 survivors with and without in-hospital rehabilitation |
| 2021 | Thorax | Frailty subtypes and recovery in older survivors of acute respiratory failure: a pilot study |
| 2021 | Thorax | Six-month and 12-month patient outcomes based on inflammatory subphenotypes in sepsis-associated ARDS: secondary analysis of SAILS-ALTOS trial |
| 2021 | Thorax | Five-year outcome of respiratory muscle weakness at intensive care unit discharge: secondary analysis of a prospective cohort study |
| 2021 | Thorax | Functional electrical stimulation-assisted cycle ergometry-based progressive mobility programme for mechanically ventilated patients: randomised controlled trial with 6 months follow-up |
| 2022 | Acta Anaesthesiol Scand | Health-related quality of life, anxiety and depression and physical recovery after critical illness - A prospective cohort study |
| 2022 | Acta Anaesthesiol Scand | Caregiver burden and emotional wellbeing in informal caregivers to ICU survivors-A prospective cohort study |
| 2022 | Acta Anaesthesiol Scand | Intensive care-treated COVID-19 patients' perception of their illness and remaining symptoms |
| 2022 | Acta Anaesthesiol Scand | Long-term cognitive and functional status in Danish ICU patients with COVID-19 |
| 2022 | Acute Med Surg | Activities of daily living and psychiatric symptoms after intensive care unit discharge among critically ill patients with or without tracheostomy: a single center longitudinal study |
| 2022 | Age & Ageing | Health-related quality of life in older patients surviving ICU treatment for COVID-19: results from an international observational study of patients older than 70ﾃつyears |
| 2022 | Am J Clin Nutr | Relation between nutrition therapy in the acute phase and outcomes of ventilated patients with COVID-19 infection: a multicenter prospective observational study |
| 2022 | Am J Crit Care | Utility of Screening for Cognitive Impairment at Hospital Discharge in Adult Survivors of Critical Illness |
| 2022 | Am J Physiol Heart Circ Physiol | Oxygen uptake kinetics and chronotropic responses to exercise are impaired in survivors of severe COVID-19 |
| 2022 | Am J Respir Crit Care Med | One-Year Mental and Physical Health Assessment in Survivors After ECMO for COVID-19-related ARDS |
| 2022 | Am J Respir Crit Care Med | One-Year Outcomes of Mechanically Ventilated COVID-19 ICU Survivors: A Prospective Cohort Study |
| 2022 | Am Surg | Chronic Critical Illness in Patients With Sepsis is Associated With Persistent Anemia, Inflammation, and Impaired Functional Outcomes |
| 2022 | Ann Am Thorac Soc | Voices from the Pandemic: A Qualitative Study of Family Experiences and Suggestions regarding the Care of Critically Ill Patients |
| 2022 | Ann Am Thorac Soc | Twelve Months and Counting: Following Clinical Outcomes in Critical COVID-19 Survivors |
| 2022 | Ann Am Thorac Soc | Post-Intensive Care Unit Syndrome in a Cohort of COVID-19 Survivors in New York City |
| 2022 | Ann Intensive Care | Result of one-year, prospective follow-up of intensive care unit survivors after SARS-CoV-2 pneumonia |
| 2022 | Ann Intern Med | Association Between Socioeconomic Disadvantage and Decline in Function, Cognition, and Mental Health After Critical Illness Among Older Adults : A Cohort Study |
| 2022 | Ann Thorac Med | Long-term COVID-19 effects on pulmonary function, exercise capacity, and health status |
| 2022 | Arch Rehabil Res Clin Transl | Complex Hallucinations in Hospitalized Rehabilitation Patients With COVID-19 |
| 2022 | Aust Crit Care | The impact of distance on post-ICU disability |
| 2022 | Aust Crit Care | Family experiences and perceptions of intensive care unit care and communication during the COVID-19 pandemic |
| 2022 | Aust Crit Care | Post-intensive care syndrome and health-related quality of life in long-term survivors of intensive care unit |
| 2022 | Aust Crit Care | Correlation of patient-reported outcome measures to performance-based function in critical care survivors: PREDICTABLE |
| 2022 | Aust Crit Care | Physical activity of patients with critical illness undergoing rehabilitation in intensive care and on the acute ward: An observational cohort study |
| 2022 | Aust Crit Care | Impact of hospitalisation on behavioural and physiological stress responses associated with cardiovascular risk in visiting family members |
| 2022 | Aust Crit Care | Interrelationships among workload, illness severity, and function on return to work following acute respiratory distress syndrome |
| 2022 | Aust Crit Care | Incidence of and risk factors for post-intensive care syndrome among Chinese respiratory intensive care unit patients: A cross-sectional, prospective study |
| 2022 | Australian Critical Care | Mixed-mode versus paper surveys for patient-reported outcomes after critical illness: A randomised controlled trial |
| 2022 | BMC Geriatr | Premorbid functional status as an outcome predictor in intensive care patients aged over 85ﾃつyears |
| 2022 | BMC Nephrol | Acute kidney injury contributes to worse physical and quality of life outcomes in survivors of critical illness |
| 2022 | BMC nursing | The role of the ICU liaison nurse services on anxiety in family caregivers of patients after ICU discharge during COVID-19 pandemic: a randomized controlled trial |
| 2022 | BMC Public Health | Modelling the potential acute and post-acute burden of COVID-19 under the Australian border re-opening plan |
| 2022 | BMC Pulm Med | Long-term dyspnea, regional ventilation distribution and peripheral lung function in COVID-19 survivors: a 1 year follow up study |
| 2022 | BMJ Open | Safety, feasibility and initial efficacy of an app-facilitated telerehabilitation (AFTER) programme for COVID-19 survivors: a pilot randomised study |
| 2022 | BMJ Open | Does a screening checklist for complex health and social care needs have potential clinical usefulness for predicting unplanned hospital readmissions in intensive care survivors: development and prospective cohort study |
| 2022 | BMJ Open | Life after COVID-19: the road from intensive care back to living - a prospective cohort study |
| 2022 | BMJ Open | Fighting COVID-19: a qualitative study into the lives of intensive care unit survivors in Wuhan, China |
| 2022 | Brain Behav Immun Health | Neuropsychological functioning in post-ICU patients after severe COVID-19 infection: The role of cognitive reserve |
| 2022 | Brain Behav Immun Health | Long-term cognitive performance and its relation to anti-inflammatory therapy in a cohort of survivors of severe COVID-19 |
| 2022 | Burns | Post Intensive Care Syndrome (PICS) physical, cognitive, and mental health outcomes 6-months to 7 years after a major burn injury: A cross-sectional study |
| 2022 | Burns (03054179) | Quantification of changes in functional capacity and muscle strength in patients: a burn intensive care unit cohort study |
| 2022 | Can J Anaesth | Outcomes of prolonged mechanical ventilation and tracheostomy in critically ill elderly patients: a historical cohort study |
| 2022 | Chest | Psychiatric Outcomes in ICU Patients With Family Visitation: A Population-Based Retrospective Cohort Study |
| 2022 | Chest | Understanding Patients' Perceived Health After Critical Illness: Analysis of Two Prospective, Longitudinal Studies of ARDS Survivors |
| 2022 | Chron Respir Dis | Pulmonary function and Quality of Life in a prospective cohort of (non-) hospitalized COVID-19 pneumonia survivors up to six months |
| 2022 | Clin EEG Neurosci | Quantitative EEG During Critical Illness Correlates with Patterns of Long-Term Cognitive Impairment |
| 2022 | Clin Epidemiol Glob Health | Post-COVID-19 mental health and its associated factors at 3-months after discharge: A case-control study |
| 2022 | Clin Neurophysiol | High-density EEG sleep correlates of cognitive and affective impairment at 12-month follow-up after COVID-19 |
| 2022 | Clin Nutr ESPEN | Association between postextubation dysphagia and physical function in survivors of critical illness: A retrospective study |
| 2022 | Clin Respir J | The role of ventilatory support for long-term outcomes after critical infection with COVID-19: A prospective cohort study |
| 2022 | Crit Care | Amniotic fluid embolism rescued by venoarterial extracorporeal membrane oxygenation |
| 2022 | Crit Care | Comparison of 6-month outcomes of sepsis versus non-sepsis critically ill patients receiving mechanical ventilation |
| 2022 | Crit Care | A multicentre evaluation exploring the impact of an integrated health and social care intervention for the caregivers of ICU survivors |
| 2022 | Crit Care | The sit-to-stand test as a patient-centered functional outcome for critical care research: a pooled analysis of five international rehabilitation studies |
| 2022 | Crit Care | ICU bereaved surrogates' comorbid psychological-distress states and their associations with prolonged grief disorder |
| 2022 | Crit Care | How symptoms of prolonged grief disorder, posttraumatic stress disorder, and depression relate to each other for grieving ICU families during the first two years of bereavement |
| 2022 | Crit Care Explor | The Prevalence of Spiritual and Social Support Needs and Their Association With Postintensive Care Syndrome Symptoms Among Critical Illness Survivors Seen in a Post-ICU Follow-Up Clinic |
| 2022 | Crit Care Explor | Post-Intensive Care COVID Survivorship Clinic: A Single-Center Experience |
| 2022 | Crit Care Explor | Six-Month Impairment in Cognition, Mental Health, and Physical Function Following COVID-19-Associated Respiratory Failure |
| 2022 | Crit Care Explor | Association Between Tracheostomy and Functional, Neuropsychological, and Healthcare Utilization Outcomes in the RECOVER Cohort |
| 2022 | Crit Care Explor | Postintensive Care Syndrome-Family Associated With COVID-19 Infection |
| 2022 | Crit Care Explor | Timing of Exposure to ICU Diaries and Its Impact on Mental Health, Memories, and Quality of Life: A Double-Blind Randomized Control Trial |
| 2022 | Crit Care Explor | Receipt of Recovery-Oriented Care Practices During Hospitalization for Sepsis |
| 2022 | Crit Care Explor | Posttraumatic Stress Disorder Symptom Clusters in Surrogate Decision Makers of Patients Experiencing Chronic Critical Illness |
| 2022 | Crit Care Explor | Psychological Attachment Orientation and Long-Term Posttraumatic Stress Symptoms Among Family Members of ICU Patients |
| 2022 | Crit Care Med | Long-Term Outcome of Severe Metabolic Acidemia in ICU Patients, a BICAR-ICU Trial Post Hoc Analysis |
| 2022 | Crit Care Med | Trajectories of Palliative Care Needs in the ICU and Long-Term Psychological Distress Symptoms |
| 2022 | Crit Care Med | In-Hospital Depressed Level of Consciousness and Long-Term Functional Outcomes in ICU Survivors |
| 2022 | Crit Care Med | Cardiorespiratory Fitness and Neuromuscular Function of Mechanically Ventilated ICU COVID-19 Patients |
| 2022 | Crit Care Med | Functional Recovery Groups in Critically Ill COVID-19 Patients and Their Associated Factors: From ICU to Hospital Discharge |
| 2022 | Crit Care Med | Patient-Centered Outcomes Following COVID-19: Frailty and Disability Transitions in Critical Care Survivors |
| 2022 | Crit Care Med | Sleep and Circadian Health of Critical COVID-19 Survivors 3 Months After Hospital Discharge |
| 2022 | Crit Care Med | Neurologic Outcomes of Survivors of COVID-19-Associated Acute Respiratory Distress Syndrome Requiring Intubation |
| 2022 | ERJ Open Res | Respiratory symptoms and radiological findings in post-acute COVID-19 syndrome |
| 2022 | Eur J Gen Pract | The impact of an intensive care unit admission on the health status of relatives of intensive care survivors: A prospective cohort study in primary care |
| 2022 | Eur J Pain | Characteristics and influence on quality of life of new-onset pain in critical COVID-19 survivors |
| 2022 | Eur J Trauma Emerg Surg | Impact of severe necrotizing fasciitis on quality of life in the Netherlands |
| 2022 | Eur Stroke J | One-year healthcare costs of patients with spontaneous intracerebral hemorrhage treated in the intensive care unit |
| 2022 | European journal of internal medicine | Durable functional limitation in patients with coronavirus disease-2019 admitted to intensive care and the effect of intermediate-dose vs standard-dose anticoagulation on functional outcomes |
| 2022 | Front Med (Lausanne) | Feasibility of Extracting Meaningful Patient Centered Outcomes From the Electronic Health Record Following Critical Illness in the Elderly |
| 2022 | Front Med (Lausanne) | Impact of Obstructive Sleep Apnea (OSA) in COVID-19 Survivors, Symptoms Changes Between 4-Months and 1 Year After the COVID-19 Infection |
| 2022 | Front Physiol | Body composition and cardiorespiratory fitness in overweight or obese people post COVID-19: A comparative study |
| 2022 | Health & Quality of Life Outcomes | Post-intensive care screening: French translation and validation of the Healthy Aging Brain Care-Monitor, hybrid version |
| 2022 | Health Psychology | Emotional distress in neuro-ICU survivor-caregiver dyads: The recovering together randomized clinical trial |
| 2022 | Health Sci Rep | Psychological consequences and the related factors among COVID-19 survivors in southeastern Iran |
| 2022 | Healthcare (Basel) | One-Year Functional Decline in COVID-19 and Non-COVID-19 Critically Ill Survivors: A Prospective Study Incorporating a Pre-ICU Status Assessment |
| 2022 | Healthcare (Basel) | The Stressful Memory Assessment Checklist for the Intensive Care Unit (SMAC-ICU): Development and Testing |
| 2022 | Heart Lung | Coping strategies, anxiety and depressive symptoms in family members of patients treated with extracorporeal membrane oxygenation: A prospective cohort study |
| 2022 | Hum Vaccin Immunother | Care pathways in invasive meningococcal disease: a retrospective analysis of the French national public health insurance database |
| 2022 | Indian J Crit Care Med | Physical Function in Critically Ill Patients during the Duration of ICU and Hospital Admission |
| 2022 | Infect Drug Resist | Health-Related Quality of Life and Associated Factors Among Covid-19 Survivors. Experience from Ethiopian Treatment Centers |
| 2022 | Infection | Clustering analysis reveals different profiles associating long-term post-COVID symptoms, COVID-19 symptoms at hospital admission and previous medical co-morbidities in previously hospitalized COVID-19 survivors |
| 2022 | Infection | Comparison of fatigue, cognitive dysfunction and psychological disorders in post-COVID patients and patients after sepsis: is there a specific constellation? |
| 2022 | Int J Environ Res Public Health | A Quality Improvement Project to Support Post-Intensive Care Unit Patients with COVID-19: Structured Telephone Support |
| 2022 | Int J Environ Res Public Health | Experiences, Emotions, and Health Consequences among COVID-19 Survivors after Intensive Care Unit Hospitalization |
| 2022 | Int J Nurs Knowl | Psychometric properties of the healthy aging brain care monitor self-report tool in patients discharged from the intensive care unit |
| 2022 | Intensive Care Med | Mental health-related quality of life is related to delirium in intensive care patients |
| 2022 | Intensive Care Med | Development and validation of early prediction models for new-onset functional impairment at hospital discharge of ICU admission |
| 2022 | Intensive Care Med | Systemic glucocorticoid use during ICU admission and symptoms of posttraumatic stress disorder in intensive care unit survivors |
| 2022 | Intensive Care Med | Long-term mortality and health-related quality of life of lower versus higher oxygenation targets in ICU patients with severe hypoxaemia |
| 2022 | Intensive Care Med Exp | Physiologic responses to exercise in survivors of critical illness: an exploratory pilot study |
| 2022 | Intensive Care Med Exp | The frailty, outcomes, recovery and care steps of critically ill patients (FORECAST) study: pilot study results |
| 2022 | Intensive Crit Care Nurs | Health-related quality of life measured with the EQ-5D-5L in critical care survivors: A cross-sectional study |
| 2022 | International journal of clinical practice | Influence on Depression, Anxiety, and Satisfaction of the Relatives' Visit to Intensive Care Units prior to Hospital Admission for Elective Cardiac Surgery: a Randomized Clinical Trial |
| 2022 | International Journal of Mental Health | Mild cognitive impairment in COVID-19 survivors: Measuring the brain fog |
| 2022 | Ir J Med Sci | Subjective symptoms and their association with psychiatric symptoms 3 months after ICU discharge: sub-analysis of a single-center prospective observational study |
| 2022 | J Acad Consult Liaison Psychiatry | Cognitive Dysfunction, Psychiatric Distress, and Functional Decline After COVID-19 |
| 2022 | J Adv Nurs | Actionable processes of care important to patients and family who experienced a prolonged intensive care unit stay: Qualitative interview study |
| 2022 | J Am Geriatr Soc | Development and validation of a prediction model for persistent functional impairment among older ICU survivors |
| 2022 | J Anesth | Quality of life and mortality among survivors of acute respiratory distress syndrome in South Korea: a nationwide cohort study |
| 2022 | J Assoc Physicians India | A Study of ICU Outcome and Long-Term Quality of Life in ICU Survivors in Central India |
| 2022 | J Cachexia Sarcopenia Muscle | MicroRNA regulatory networks associated with abnormal muscle repair in survivors of critical illness |
| 2022 | J Cardiothorac Vasc Anesth | Six-Month Quality of Life in COVID-19 Intensive Care Unit Survivors |
| 2022 | J Cardiothorac Vasc Anesth | One-Year Multidisciplinary Follow-Up of Patients With COVID-19 Requiring Invasive Mechanical Ventilation |
| 2022 | J Clin Med | Prevalence and Risk Factor Analysis of Post-Intensive Care Syndrome in Patients with COVID-19 Requiring Mechanical Ventilation: A Multicenter Prospective Observational Study |
| 2022 | J Clin Med | Prevalence and Long-Term Prognosis of Post-Intensive Care Syndrome after Sepsis: A Single-Center Prospective Observational Study |
| 2022 | J Clin Med | Post-Intensive Care Syndrome in Non-COVID-19 ICU Survivors during the COVID-19 Pandemic in South Korea: A Multicenter Prospective Cohort Study |
| 2022 | J Clin Med | Early Intensive Physical Rehabilitation Combined with a Protocolized Decannulation Process in Tracheostomized Survivors from Severe COVID-19 Pneumonia with Chronic Critical Illness |
| 2022 | J Clin Med | Incidence and Risk Factors of Worsened Activities of Daily Living Status Three Months after Intensive Care Unit Discharge among Critically Ill Patients: A Prospective Cohort Study |
| 2022 | J Clin Med | A Modified Physical Disability Screening Model after Treatment in the Intensive Care Unit: A Nationwide Derivation-Validation Study |
| 2022 | J Clin Nurs | The effect of structured virtual patient visits (sVPVs) on COVID-19 patients and relatives' anxiety levels in intensive care unit |
| 2022 | J Crit Care | Health-related quality of life, one-year costs and economic evaluation in extracorporeal membrane oxygenation in critically ill adults |
| 2022 | J Crit Care | Association between out-of-bed mobilization during the ICU stay of elderly patients and long-term autonomy: A cohort study |
| 2022 | J Intensive Care | Association between the presence of delirium during intensive care unit admission and cognitive impairment or psychiatric problems: the Korean ICU National Data Study |
| 2022 | J Intensive Care Med | Long-Term Follow up of Renal and Other Acute Organ Failure in Survivors of Critical Illness Due to Covid-19 |
| 2022 | J Intensive Care Med | Self-perceived recovery and quality of life in elderly patients surviving ICU-admission for abdominal sepsis |
| 2022 | J Intensive Care Med | Survivors of Acute Lung Injury Have Greater Impairments in Strength and Exercise Capacity Than Survivors of Other Critical Illnesses as Measured Shortly After ICU Discharge |
| 2022 | J Intensive Care Med | Patients Surviving Critical COVID-19 have Impairments in Dual-task Performance Related to Post-intensive Care Syndrome |
| 2022 | J Intensive Care Med | Intensive Care Unit- Acquired Weakness and Hospital Functional Mobility Outcomes Following Invasive Mechanical Ventilation in Patients with COVID-19: A Single-Centre Prospective Cohort Study |
| 2022 | J Korean Med Sci | Comprehensive Rehabilitation in Severely Ill Inpatients With COVID-19: A Cohort Study in a Tertiary Hospital |
| 2022 | J Nephrol | Long-lasting clinical symptoms 6ﾃ・ﾂmonths after COVID-19 infection in the French national cohort of patients on dialysis |
| 2022 | J Nerv Ment Dis | COVID-19 Survivors' Intensive Care Unit Experiences and Their Possible Effects on Mental Health: A Qualitative Study |
| 2022 | J Pers Med | Perceived Symptoms, Mental Health and Quality of Life after Hospitalization in COVID-19 Patients |
| 2022 | J Pers Med | Depression, Insomnia and Post-Traumatic Stress Disorder in COVID-19 Survivors: Role of Gender and Impact on Quality of Life |
| 2022 | J Rehabil Med | Post-Intensive Care Syndrome Prevalence Six Months after Critical Covid-19: Comparison between First and Second Waves |
| 2022 | J Thorac Cardiovasc Surg | Early posthospitalization recovery after extracorporeal membrane oxygenation in survivors of COVID-19 |
| 2022 | J Trauma Acute Care Surg | Patient-reported outcomes 6 to 12 months after isolated rib fractures: A nontrivial injury pattern |
| 2022 | JAMA | Association of COVID-19 Acute Respiratory Distress Syndrome With Symptoms of Posttraumatic Stress Disorder in Family Members After ICU Discharge |
| 2022 | JAMA Internal Medicine | Stress-Related Disorders of Family Members of Patients Admitted to the Intensive Care Unit With COVID-19 |
| 2022 | JAMA Netw Open | Two-Year Health Outcomes in Hospitalized COVID-19 Survivors in China |
| 2022 | JAMA Psychiatry | Neuropsychiatric and Cognitive Outcomes in Patients 6 Months After COVID-19 Requiring Hospitalization Compared With Matched Control Patients Hospitalized for Non-COVID-19 Illness |
| 2022 | JAMA: Journal of the American Medical Association | Association of Extracorporeal Membrane Oxygenation With New Mental Health Diagnoses in Adult Survivors of Critical Illness |
| 2022 | Journal of medical Internet research | Intensive Care Unit-Specific Virtual Reality for Critically Ill Patients With COVID-19: multicenter Randomized Controlled Trial |
| 2022 | Journal of stroke and cerebrovascular diseases | Collaborative Integration of Palliative Care in Critically Ill Stroke Patients in the Neurocritical Care Unit: a Single Center Pilot Study |
| 2022 | JPEN Journal of Parenteral & Enteral Nutrition | Physical recovery of COVID-19 pneumosepsis intensive care survivors compared with non-COVID pneumosepsis intensive care survivors during post-intensive care hospitalization: The RECOVID retrospective cohort study |
| 2022 | Med Intensiva (Engl Ed) | Post-intensive care syndrome one month after discharge in surviving critically ill COVID-19 patients |
| 2022 | N Engl J Med | Early Active Mobilization during Mechanical Ventilation in the ICU |
| 2022 | New England journal of medicine | Blood-Pressure Targets in Comatose Survivors of Cardiac Arrest |
| 2022 | Nursing in Critical Care | Patients' and relatives' experiences of post-ICU everyday life: A qualitative study |
| 2022 | P R Health Sci J | Pain, Anxiety, and the Continuous Use of Opioids and Benzodiazepines in Trauma Intensive Care Unit Survivors: An Exploratory Study |
| 2022 | Palliative & Supportive Care | Development and preliminary evaluation of EMPOWER for surrogate decision-makers of critically ill patients |
| 2022 | Physiother Theory Pract | Extended physiotherapy after Intensive Care Unit (ICU) stay: A prospective pilot study with a before and after design |
| 2022 | PLoS One | Long-term health-related quality of life, healthcare utilisation and back-to-work activities in intensive care unit survivors: Prospective confirmatory study from the Frisian aftercare cohort |
| 2022 | PLoS One | Association of analgosedation with psychiatric symptoms and health-related quality of life in ARDS survivors: Post hoc analyses of the DACAPO study |
| 2022 | PLoS One | Comparison between the persistence of post COVID-19 symptoms on critical patients requiring invasive mechanical ventilation and non-critical patients |
| 2022 | PLoS One | Employment status and its associated factors for patients 12 months after intensive care: Secondary analysis of the SMAP-HoPe study |
| 2022 | PLoS One | Post-intensive care syndrome in out-of-hospital cardiac arrest patients: A prospective observational cohort study |
| 2022 | Professioni Infermieristiche | Chronic pain in in survivors of critical illness: prevalence and associated psychological disorders |
| 2022 | Psychiatr Q | Depressive and Anxiety Symptoms in Severe COVID-19 Survivors: A Prospective Cohort Study |
| 2022 | Respir Med | Functional limitations 12 months after SARS-CoV-2 infection correlate with initial disease severity: An observational study of cardiopulmonary exercise capacity testing in COVID-19 convalescents |
| 2022 | Respir Med Res | Fibrotic-like abnormalities notably prevalent one year after hospitalization with COVID-19 |
| 2022 | Respiration | Fatigue and Dyspnoea as Main Persistent Post-COVID-19 Symptoms in Previously Hospitalized Patients: Related Functional Limitations and Disability |
| 2022 | Rev Bras Ter Intensiva | The impact of severe COVID-19 on health-related quality of life and disability: an early follow-up perspective |
| 2022 | Rev Neurol (Paris) | Prevalence and prospective evaluation of cognitive dysfunctions after SARS due to SARS-CoV-2 virus. The COgnitiVID study |
| 2022 | S Afr Med J | Long-stay medical-surgical intensive care unit patients in South Africa: Quality of life and mortality 1 year after discharge |
| 2022 | Sci Rep | Prevalence of acute neurological complications and pathological neuroimaging findings in critically ill COVID-19 patients with and without VV-ECMO treatment |
| 2022 | Sci Rep | Prevalence of self-reported fatigue in intensive care unit survivors 6 months-5 years after discharge |
| 2022 | Sci Rep | Prevalence of post-intensive care syndrome in mechanically ventilated patients with COVID-19 |
| 2022 | Scientific reports | Assessment of 5-year outcomes of life satisfaction in survivors after rehabilitation programs: a multicenter clinical trial |
| 2022 | Sleep Medicine | Undiagnosed sleep disorder breathing as a risk factor for critical COVID-19 and pulmonary consequences at the midterm follow-up |
| 2022 | Thorax | Evaluation of a health and social care programme to improve outcomes following critical illness: a multicentre study |
| 2022 | Thorax | Physical, cognitive and mental health outcomes in 1-year survivors of COVID-19-associated ARDS |
| 2022 | Thorax | Association of imbalance between job workload and functional ability with return to work in ARDS survivors |
| 2022 | Turkish Thoracic Journal / Turk Toraks Dergisi | Six-Week Hospital-Based Pulmonary Rehabilitation in Covid Pneumonia ICU Survivors: Experience from a Tertiary Care Center in Central India |
| 2022 | Value Health Reg Issues | Quality of Life of Family Caregivers of Critically Ill Patients With Cancer Before and After Intensive Care Unit Admission Measured by EQ-5D 3-Level: A Longitudinal Prospective Cohort Study |

**Table S1. Reference list of 754 included studies in scoping review**

**4. Results of the scoping review at hospital discharge**

| **Physical function at hospital discharge** | **Frequency** |
| --- | --- |
| MRC (Medical Research Council) score | 22 |
| Grip strength | 20 |
| 6-minute walk test | 11 |
| Physical Function ICU Test (PFIT) | 8 |
| Functional Status Score for the ICU (FSS-ICU) | 4 |
| Chelsea Critical Care Physical Assessment tool (CPAx) | 4 |
| Short Physical Performance Battery (SPPB) | 4 |
| Functional Ambulation Categories (FAC) | 3 |
| Clinical Frailty Scale (CFS) | 3 |
| Manual muscle test (MMT) | 2 |
| Sit-to-stand test (30 s, 1 min) | 2 |
| Medical Research Council Dyspnea (MRCD) Scale | 2 |
| DE Morton Mobility Index (DEMMI) | 2 |
| Pulmonary function including Spirometer, Diffusing capacity of Lung for Carbon monOxide (DLCO) | 2 |
| 2-minute walk test | 1 |
| CFS | 1 |
| Godin Leisure-Time Exercise questionnaire | 1 |
| Continuous Scale-Physical Functional Performance-10 (CS-PFP-10) | 1 |
| Quadriceps muscle strength | 1 |
| Symbol Digit Modalities Test (SDMT) | 1 |
| Brain Arrest Neurological Outcome Scale (BrANOS) | 1 |
| ASIA (American Spinal Cord Injury Association) motor score | 1 |
| Timed Up and Go test (TUG) | 1 |
| Cognitive function at hospital discharge | Frequency |
| Mini-Mental State Examination (MMSE) | 16 |
| Montreal Cognitive Assessment (MoCA) | 11 |
| Mini-Cognitive Assessment Instrument (Mini-Cog) | 2 |
| Short Memory Questionnaire (SMQ) | 2 |
| Informant Questionnaire for Cognitive Decline in the Elderly (IQCODE) | 2 |
| Hayling Sentence Completion Test (HSCT) scaled score | 1 |
| Verbal Fluency Test (VFT) total score | 1 |
| Repeatable Battery for the Assessment of Neuropsychological Status (RBANS) | 1 |
| Trail Making Test A, B | 1 |
| Rey Auditory-Verbal Learning Test (RAVLT) | 1 |
| Wechsler Adult Intelligence Scale (WAIS)-IV | 1 |
| Mental illness at hospital discharge | Frequency |
| Hospital Anxiety and Depression Scale (HADS) | 31 |
| Impact of Event Scale-Revised (IES-R) | 22 |
| Visual Analogue Scale (VAS) | 12 |
| Patient Health Questionnaire-9 (PHQ-9) | 5 |
| PTSD Checklist for DSM-5 (PCL-5) | 5 |
| Depression Anxiety Stress Scale-21 (DASS-21) | 4 |
| Zung Self-Rating Anxiety Scale (SAS) | 2 |
| PHQ-2 | 2 |
| IES-6 | 2 |
| Generalized Anxiety Disorder-7 (GAD-7) | 1 |
| Quick Inventory of Depressive Symptomatology-Self Report 16 (QIDS-SR16) | 1 |
| Activities of Daily Living (ADL) at hospital discharge | Frequency |
| Barthel Index (BI) | 20 |
| Functional Independence Measure (FIM) | 10 |
| Cerebral Performance Category (CPC) | 4 |
| modified Rankin Scale (mRS) | 4 |
| Binary ADL staircases questionnaire | 1 |
| Scores of Independence for Neurologic and Geriatric Rehabilitation (SINGER) | 1 |
| Quality of Life (QOL) at hospital discharge | Frequency |
| Short Form-36 (SF-36) | 42 |
| EuroQol-5Dimension-5Level (EQ-5D-5L) | 15 |
| EuroQol-5Dimension-3Level (EQ-5D-3L) | 8 |
| EuroQol-Visual Analogue Scale (EQ-5D-VAS) | 4 |
| EuroQol-6Dimension-3Level (EQ-6D-3L) | 1 |
| Health-Related QOL (HRQOL) | 1 |
| SF-12 | 1 |
| Pain at hospital discharge | Frequency |
| Numerical Rating Scale (NRS) | 2 |
| Fatigue at hospital discharge | Frequency |
| Functional Assessment of Chronic Illness Therapy-Fatigue (FACIT-F) | 1 |
| Others at hospital discharge | Frequency |
| ICU-Memory Tool (ICU-MT) | 1 |
| Family at hospital discharge | Frequency |
| HADS | 17 |
| Family Satisfaction in the Intensive Care Unit (FS-ICU) | 1 |
| Post-traumatic Stress Scale 10 (PTSS-10) | 1 |
| Spielberger State Anxiety Inventory | 1 |

**Table S2. Extracted PICS assessment at hospital discharge**

**5. Results of the scoping review after hospital discharge**

| Physical function after hospital discharge | Frequency |
| --- | --- |
| 6-minute walk test | 49 |
| Pulmonary function including Spirometer, Diffusing capacity of Lung for Carbon monOxide (DLCO) | 39 |
| MRC (Medical Research Council) score | 34 |
| Grip strength | 34 |
| Clinical Frailty Scale (CFS) | 16 |
| Sit-to-stand test (30 s, 1 min) | 15 |
| Short Physical Performance Battery (SPPB) | 14 |
| 4-m gait speed test | 8 |
| Manual muscle test (MMT) | 6 |
| MRC dyspnea scale | 6 |
| Saint George’s Respiratory Questionnaire (SGRQ) | 6 |
| Isometric Quadriceps Strength | 6 |
| Timed Up and Go test (TUG) | 5 |
| BERG balance test | 4 |
| Physical Functional Status (PFS) | 4 |
| Chelsea critical care Physical Assessment tool (CPAx) | 4 |
| Borg Dyspnea Scale | 4 |
| 10-minute walk test | 3 |
| 2-minute walk test | 3 |
| Fried Frailty Criteria | 3 |
| Four-stage balance test | 2 |
| Patient Reported Outcomes Measurement and Information System-Short Form (PROMIS-SF) | 2 |
| International Physical Activity Questionnaires-Short Form IPAQ-SF () | 2 |
| Modified Given Symptom Assessment Tool | 2 |
| Functional Ambulation Categories (FAC) | 2 |
| Continuous Scale-Physical Functional Performance (CS-PFP-10) | 1 |
| Godin Leisure-Time Exercise questionnaire | 1 |
| Multidimensional Dyspnea Profile | 1 |
| Connor–Davidson Resiliency 10-Item Scale | 1 |
| American Spinal Cord Injury Association (ASIA) motor score | 1 |
| Physical Activity Scale for the Elderly (PASE) | 1 |
| Extra Short Musculoskeletal Function Assessment (XSFMA) | 1 |
| Activity Measure for Post-Acute Care (AMPAC) | 1 |
| Physical with Burns Specific Health Scale-Brief (BSHS-B) | 1 |
| Dependence Nursing Scale | 1 |
| Takahashi test | 1 |
| Incremental Shuttle Walk Test (ISWT) | 1 |
| Murray Lung Injury Score (MLIS) | 1 |
| Behavior Rating Inventory of Executive Function-Adult | 1 |
| Disabilities of Arm, Shoulder, and Hand (DASH) score | 1 |
| Sydney Swallowing Questionnaire (SSQ) | 1 |
| Judgment-based frailty assessment tool | 1 |
| Physical Functional test for the ICU (PFIT) | 1 |
| Canadian Study of Health and Aging Clinical Frailty Scale score | 1 |
| Net Promotor Score (NPS) | 1 |
| Functional Oral Intake Scale ()FOIS | 1 |
| Short Nutritional Assessment Questionnaire 65+ (SNAQ65+) | 1 |
| Physical Self-Maintenance Scale (PSMS) | 1 |
| Physical function shortform 8b | 1 |
| Behavior Rating Inventory of Executive Function Initiation (BRIEF-I) | 1 |
| Cognitive function after hospital discharge | Frequency |
| Montreal Cognitive Assessment (MoCA) | 37 |
| Mini-Mental State Examination (MMSE) | 36 |
| Repeatable Battery for the Assessment of Neuropsychological Status (RBANS) | 21 |
| Trail Making Test A, B | 15 |
| Telephone Interview for Cognitive Status (TICS) including the modified Japanese version | 9 |
| Short Memory Questionnaire (SMQ) | 7 |
| Informant Questionnaire for Cognitive Decline in the Elderly (IQCODE) | 7 |
| Cognitive Failures Questionnaire (CFQ) | 7 |
| Cognitive and Affective Mindfulness Scale (CAMS) revised version | 5 |
| Wechsler Adult Intelligence Scale (WAIS)-IV | 4 |
| Healthy Aging Brain Care Monitor Self Report Tool | 4 |
| Rey Auditory-Verbal Learning Test (RAVLT) | 4 |
| Cambridge Neuropsychological Test Automated Battery (CANTAB) | 3 |
| Clinical Dementia Rating (CDR) | 3 |
| Stroop Color and Word Test (SCWT) | 2 |
| Benton Visual Retention test | 2 |
| Stroop Color and Word test | 2 |
| Mini-Cognitive Assessment Instrument (Mini-Cog) | 1 |
| Brain Arrest Neurological Outcome Scale (BrANOS) | 1 |
| Generalized Anxiety Disorder-7 (GAD-7) | 1 |
| Functional Assessment of Cancer Therapy - Cognitive Function (FACT-Cog) | 1 |
| Rey–Osterreith Complex Figure Test (ROCFT) | 1 |
| D-KEFS Verbal Fluency | 1 |
| 6-month Hayling test of executive function | 1 |
| Cognitive Difficulties Self-Rating Scale (CDS) | 1 |
| Subjective Cognitive Impairment (SCI) | 1 |
| Level of Cognitive Functioning Scale (LCFS) | 1 |
| Revised Coma Recovery Scale (CRS-R) | 1 |
| National Adult Reading Test (NART) | 1 |
| Wechsler Memory Scale (WMS) | 1 |
| Harvard Trauma Questionnaire Part IV | 1 |
| Dementia-related activities of daily living | 1 |
| DEMentia Toolkit for Effective Communication (DEMTEC) | 1 |
| Functional Comorbidity Index | 1 |
| Knaus’ functional classification | 1 |
| Hopkins Verbal Learning Test (HVLT) | 1 |
| Controlled Oral Word Association (COWA) | 1 |
| Cancellation and detection (CAT) test | 1 |
| Psychogeriatric Assessment Scale (PAS) | 1 |
| Consortium to Establish a Registry for Alzheimer’s Disease Neuropsychological Battery (CERAD-NB) | 1 |
| Mental illness after hospital discharge | Frequency |
| Hospital Anxiety and Depression Scale (HADS) | 155 |
| Impact of Event Scale-Revised (IES-R) | 76 |
| Patient Health Questionnaire-9 (PHQ-9) | 26 |
| Post-traumatic Stress Scale 10 (PTSS-10) | 20 |
| PTSD Checklist for DSM-5 (PCL-5) | 18 |
| BDI-II (Beck Depression Inventory-II) | 16 |
| GAD-7 | 13 |
| PTSD Checklist, Civilian version (PCL-C) | 12 |
| IES | 11 |
| PTSS-14 | 11 |
| PCL-Specific (PCL-S) | 9 |
| IES-6 | 8 |
| PHQ-2 | 7 |
| Numerical Rating Scale (NRS) | 5 |
| Depression Anxiety Stress Scale (DASS)-21 | 5 |
| State Trait Anxiety Inventory (STAI) | 5 |
| Centre for Epidemiological Studies-Depression Scale (CES-D) | 5 |
| Visual Analog Scale-Anxiety (VAS-A) | 4 |
| Brief COPE (Coping Orientation to Problems Experienced) Inventory | 4 |
| Trauma Screening Questionnaire | 4 |
| Major Depression Inventory (MDI) | 3 |
| PHQ-8 | 3 |
| PHQ-4 | 3 |
| Davidson Trauma Scale (DTS) | 3 |
| Zung Self-Rating Anxiety Scale (SAS) | 2 |
| Zung Self-Rating Depression Scale (SDS) | 2 |
| Hamilton Anxiety Rating Scale (HARS) | 2 |
| Faces Anxiety Scale | 2 |
| Likert Scale | 2 |
| Brief Symptom Inventory (BSI) | 2 |
| Intensive care Psychological Assessment Tool (IPAT) score | 2 |
| Global Psychotrauma Screen (GPS) | 2 |
| Geriatric Depression scale-30 (GDS-30) | 2 |
| Post-traumatic Diagnostic Scale (PDS) | 2 |
| PHQ-10 | 1 |
| Kessler-10 | 1 |
| Center for Epidemiologic Studies Depression-10 items (CESD-10) | 1 |
| Shortened Profile of Mood States-Anxiety (POMS-A) subscale | 1 |
| Acute Stress Disorder Scale (ASDS) | 1 |
| Quick Inventory of Depressive Symptomatology-Self Report 16 (QIDS-SR16) | 1 |
| Herth Hope Index | 1 |
| Escala de Gravedad de Síntomas del Trastorno de estrés posttraumático (EGS) (Severity of PTSD Symptoms Scale) | 1 |
| Inventory of Complicated Grief-Revised | 1 |
| Coping Style Questionnaire | 1 |
| Sense of Coherence (SOC) scale | 1 |
| Coping Inventory for Stressful Situations (CISS-21) | 1 |
| Clinician-Administered PTSD Scale (CAPS) | 1 |
| Peritraumatic Distress Inventory (PDI) | 1 |
| Brief Experiential Avoidance Questionnaire (BEAQ) | 1 |
| Primary Care PTSD (PC-PTSD) Screen | 1 |
| ADL after hospital discharge | Frequency |
| Barthel Index | 47 |
| Instrumental Activities of Daily Living (IADL) | 25 |
| modified Rankin Scale (mRS) | 19 |
| Katz Activities of Daily Living | 19 |
| Functional Independence Measure (FIM) | 12 |
| 12-item World Health Organization Disability Assessment Schedule (WHODAS 2.0) | 11 |
| Glasgow Outcome Scale (GOS) | 9 |
| GOS-Extended (GOS-E) | 9 |
| Cerebral Performance Category (CPC) | 8 |
| Functional Activities Questionnaire (FAQ) | 8 |
| Functional Performance Inventory (FPI) | 4 |
| Zubrod score | 3 |
| Disability Rating Scale (DRS) | 3 |
| Overall Performance Category (OPC) | 2 |
| Pfeffer Functional Activities Questionnaire | 2 |
| Korean Activities of Daily Living scale | 2 |
| Karnofsky performance status scale | 2 |
| Post Covid-19 Functional Status Scale | 1 |
| General Perceived Self-Efficacy Scale | 1 |
| Sickness Impact Profile 68 (SIP68) | 1 |
| Functional Impairment Checklist (FIC) | 1 |
| Scores of Independence for Neurologic and Geriatric Rehabilitation (SINGER) | 1 |
| QOL after hospital discharge | Frequency |
| Short Form-36 Including RAND-36 (SF-36) | 153 |
| EuroQol-5Dimension-5Level (EQ-5D-5L) | 70 |
| EuroQol-5Dimension-3Level (EQ-5D-3L) | 46 |
| EuroQol-Visual Analogue Scale (EQ-VAS) | 39 |
| SF-12 | 26 |
| World Health Organization Quality of Life-brief questionnaire (WHOQOL) | 5 |
| Quality-Adjusted Life Year (QALY) | 4 |
| Assessment of Quality of Life 4D (AQoL-4D) | 4 |
| 15D instrument | 3 |
| EuroQol-6Dimension-3Level (EQ-6D-3L) | 2 |
| SF-8 | 2 |
| Post-COVID-19 Functional Status (PCFS) Scale | 2 |
| Health-Related QOL (HRQOL) | 1 |
| Quality of Life after Brain Injury (QOLIBRI) | 1 |
| McGill Quality of Life | 1 |
| Blake questionnaire | 1 |
| Recovery After Intensive Care (RAIN) | 1 |
| Nottingham Health Profile (NHP) | 1 |
| Health Utilities Index-3 | 1 |
| Sickness Impact Profile (SIP) | 1 |
| T-QoL questionnaire statement (T-QoL) | 1 |
| Sleep after hospital discharge | Frequency |
| Pittsburgh Sleep Quality Index (PSQI) | 9 |
| Insomnia severity index (ISI) | 8 |
| Actigraphy | 3 |
| Polysomnography | 2 |
| Epworth Sleepiness Scale | 2 |
| STOP-BANG questionnaire | 1 |
| General Sleep Disturbance Scale | 1 |
| Pain after hospital discharge | Frequency |
| Brief Pain Inventory | 13 |
| NRS | 5 |
| VAS | 3 |
| Graded Chronic Pain Scale (GCPS) | 3 |
| Neuropathic Symptoms Score (NSS) | 2 |
| German pain questionnaire | 2 |
| Fatigue after hospital discharge | Frequency |
| Fatigue Severity Scale (FSS) | 3 |
| Fatigue Assessment Scale (FAS) | 3 |
| Functional Assessment of Chronic Illness Therapy-Fatigue (FACIT-F) | 2 |
| Multidimensional Fatigue Inventory (MFI) | 2 |
| Revised-Piper Fatigue Scale | 1 |
| 18 items Lee Fatigue Scale | 1 |
| Others at hospital discharge | Frequency |
| Measure of Current Status part A (MOCS-A) | 4 |
| General Self Efficacy (GSE) scale | 3 |
| Intimate Bond Measure (IBM) | 2 |
| Alcohol Use Disorders Identification Test | 2 |
| ICU-Memory Tool (ICU-MT) | 2 |
| The Life Space Questionnaire | 1 |
| Crisis Support Scale (CSS) | 1 |
| Cut down, Annoyed by criticism, Guilty feeling, Eye-opener (CAGE) | 1 |
| Inventory of Complicated Grief (ICG) | 1 |
| Alcohol Use Disorders Identification Test-C (AUDIT-C) | 1 |
| Work Ability Index | 1 |
| Patient Reported Experience Measure (PREM) | 1 |
| Controlled Oral Word Association test (COWA) | 1 |
| Family after hospital discharge | Frequency |
| SF-36 | 47 |
| HADS | 34 |
| IES-R | 19 |
| Family Satisfaction in the ICU (FS-ICU) | 10 |
| PTSD Checklist-Specific (PCL-S) | 7 |
| IES | 7 |
| PCL-C | 5 |
| PHQ-9 | 5 |
| Center for Epidemiologic Studies-Depression (CES-D) | 5 |
| PSQI | 5 |
| Quality of Death and Dying (QODD) | 3 |
| PHQ-8 | 3 |
| Zarit Burden Interview-12 items (Zarit-12) | 3 |
| General Functioning sub-scale from the McMaster Family Assessment Device (GF sub-scale from the FAD) | 2 |
| Brief COPE Inventory | 2 |
| Shortened version of CES-D (Shortened CES-D) | 2 |
| Caregiver Health Behavior (CHB) | 2 |
| Caregiver Strain Index (CSI) | 2 |
| Prolonged Grief Disorder-13 | 2 |
| IES-6 | 1 |
| DASS-21 | 1 |
| CES-D | 1 |
| Actigraphy | 1 |
| Family Sense of Coherence (F-SOC) | 1 |
| Family Member Experiences at End of Life (FEEL) | 1 |
| Herth Hope Index (HHI) | 1 |
| STAI | 1 |
| Composite International Diagnostic Interview-Short Form (CIDI-SF) | 1 |
| Functional Assessment of Cancer Therapy-Cognitive Function (FACT-Cog) | 1 |
| Core Bereavement Items Questionnaire (CBI-17) | 1 |
| National Institutes of Health Toolbox Emotion Battery (NIHTB-EB) | 1 |
| Positive Affect Scale of the Positive and Negative Affect Schedule (PANAS) | 1 |
| 20-item Medical Outcomes Study Social Support Survey | 1 |
| Pearlin and Schooler’s 7-item Mastery Scale | 1 |
| 17-item Caregiver Assistance Scale | 1 |
| 14-item Caregiving Impact Scale | 1 |
| 4-item Personal Gain Scale | 1 |
| Positive Aspects of Caregiving (PAC) | 1 |
| Family Strain Questionnaire short form | 1 |
| Needs at the End-of-Life Screening Tool (NEST) | 1 |
| GAD-7 | 1 |
| Critical Care Family Needs Inventory (CCFNI) | 1 |
| Caregiver Assistance Scale | 1 |
| Satisfaction of the Relatives of the Inpatient Patients (SASRIP) | 1 |
| Family Decision-Making Self-efficacy Scale | 1 |
| Caregiver version of HABC-M (HABC-MCG) | 1 |

**Table S3. Extracted PICS assessment after hospital discharge**
